# β-Lactamase cleavable antimicrobial peptide–drug conjugates

**DOI:** 10.1039/d5sc06369h

**Published:** 2025-09-19

**Authors:** Tomas Deingruber, Josephine S. Gaynord, Bee Ha Gan, Kristina A. Kostadinova, Thomas J. O'Brien, Yaw Sing Tan, Jeremy S. Parker, Thomas A. Hunt, Jason S. Carroll, Martin Welch, David R. Spring

**Affiliations:** a Yusuf Hamied Department of Chemistry, University of Cambridge Lensfield Road Cambridge UK spring@ch.cam.ac.uk; b Department of Biochemistry, University of Cambridge Hopkins Building Tennis Court Road Cambridge UK mw240@cam.ac.uk; c Bioinformatics Institute, Agency for Science, Technology and Research (A*STAR) Singapore; d Early Chemical Development, Pharmaceutical Development, R&D, AstraZeneca Macclesfield UK; e Oncology R&D, AstraZeneca Cambridge UK; f Cancer Research UK Cambridge Institute Robinson Way Cambridge CB2 ORE UK

## Abstract

Antimicrobial resistance attracts a considerable amount of attention as it threatens the efficiency of current antibacterial treatments. Besides a more considerate use of current antibiotics to slow down the spread of antimicrobial resistance, there is ample need for new therapeutic avenues to treat already resistant strains. Here, we describe the use of a cleavable peptide–drug conjugate to target bacteria with diverse resistance strategies. The conjugate consists of three main components: a β-lactamase cleavable linker, a positively charged stapled antimicrobial peptide, and an antibiotic. The linker ensures selective cleavage and provides the prospect of lowering systemic toxicity of the conjugate. The positively charged peptide targets the negatively charged bacterial membrane, and stapling pre-organises it in a helical structure. Finally, the drug provides another, distinct mode of action to the peptide, which should overall reduce the development of resistance. A series of peptides was prepared and the most promising one was then developed into a stapled conjugate. The factors affecting the activity of this conjugate were investigated, proving cleavage by β-lactamase and superior potency compared to the non-cleavable control, as shown by its minimal inhibitory concentrations.

## Abstract 2

From a series of peptides, the most promising was developed into a stapled conjugate, with a ciprofloxacin prodrug attached to the staple *via* a cephalosporin linker. Factors influencing antibacterial activity and drug release were investigated.

## Graphical abstract

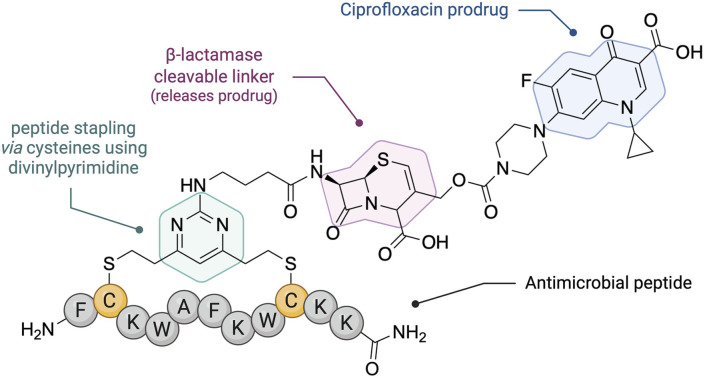

## 1 Introduction

Bacteria, like other living entities, are constantly adapting to their environment. In a healthcare context, this means that they are finding ways to circumvent antibacterial treatments, many of which have been around for over five decades and seen limited advancement.^1^ The rise in antibacterial, and in broader terms antimicrobial, resistance has been troubling health experts. It is estimated that in the future more people could die of resistant infectious diseases than are now dying of cancer.^2^

Two of the many possible methods for combating this resistance are the development of non-traditional antibiotic compounds and identification of new combinations of traditional drugs.^3,4^ Modern antibiotic treatments are typically small molecule-based and only a few are of peptidic nature.^5,6^ Antimicrobial peptides (AMPs) are a promising type of antibacterial treatment. They often show a membrane-targeting mechanism of action, which is novel compared to the standard protein targets of small molecules and which carries an additional benefit of low resistance propensity.^5^ When optimised, AMPs show good selectivity for bacteria due to the distinct membrane composition between bacterial and mammalian cells. However, the chemical composition of AMPs can also limit their activity in biological settings. For example, physiological salt concentrations often give different activity levels compared to *in vitro* assays, or peptides can be rapidly degraded by proteases.^7^ Cyclisation of peptides (found in many of the few clinically-used peptides such as polymyxin B or daptomycin) can alleviate some of these disadvantages. In addition, linear AMPs often need to organise at the surface of the membrane into an active conformation in order to interact with the membrane. In such cases, cyclisation can help to pre-organise the peptide in its active conformation.^5^

**Fig. 1:**
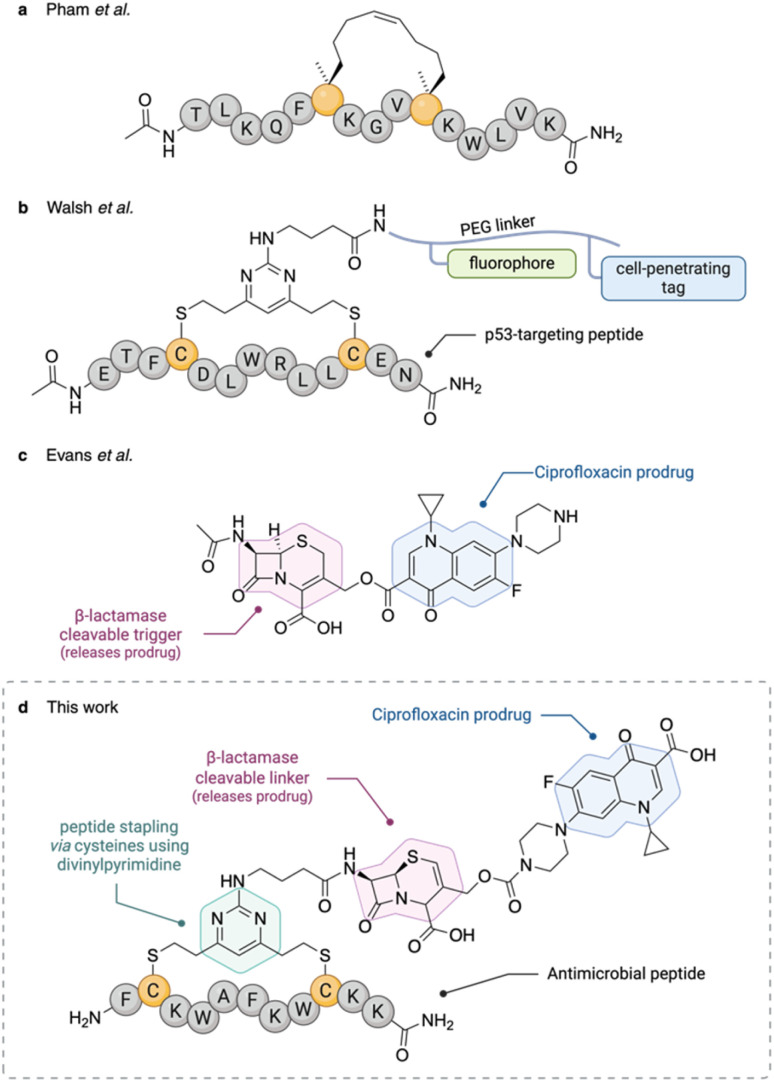
Previous research that this work (d) builds on. It combines the concepts of stapled antimicrobial peptides (*e.g.* work by Pham *et al.*, (a))^11^ with β-lactamase cleavable ciprofloxacin prodrugs (Evans *et al.*, (c)).^15^ In the context of this work, the prodrug will be attached to the peptide *via* a functionalised staple and the divinylpyrimidine methodology previously reported by the Spring group (b) will be used for the stapling.^13^

Peptides can be artificially cyclised by using stapling methodologies. There are several types of peptide stapling, which have been reviewed elsewhere.^8,9^ In the context of antimicrobial peptides, metathesis-based stapling has been used extensively, as reviewed by Migoń *et al.*^10^ For example, Pham *et al.* have prepared derivatives of AMP esculentin-2EM, stapled using metathesis of alkene-bearing unnatural amino acid residues incorporated in the peptide sequence.^11^ The stapled peptides (Fig. 1a) exhibited an improved inhibitory activity against a panel of Gram-positive and Gram-negative bacteria, compared to the unstapled parent peptides. Our work expands the area of stapled AMPs by replacing the one-component metathesis stapling with a two-component stapling methodology mediated by divinylpyridine and divinyltriazine moieties developed in the Spring group.^12,13^ Unlike hydrocarbon-based stapling, which requires introduction of unnatural amino acids into the peptide sequence, the divinyl-heteroarene stapling relies on canonical cysteine residues (Fig. 1b). In addition, this methodology allows for introduction of the functionalised staple after peptide synthesis, which makes the synthesis more convergent, hence reducing the risk of peptide side-reactivity.

To minimise the toxicity to human cells, antibacterial prodrugs can be used to ensure selective targeting of bacterial cells. The concept of antibacterial prodrugs has been previously demonstrated by several research groups. For example, Liu *et al.* joined a siderophore to a derivative of oxazolidinone antibiotic eperezolid *via* a β-lactamase cleavable cephalosporin.^14^ The siderophore directs the conjugate to the periplasm where β-lactamase is localised and hence the oxazolidininone is released. Similarly, Evans *et al.* have used the same β-lactamase cleavable moiety to release ciprofloxacin from a prodrug (Fig. 1c).^15^ Although β-lactamase cleavable pro-moieties are the focus of this paper, other mechanisms of antibacterial prodrug release have been reported, such as in a nitroreductase-cleavable ciprofloxacin prodrug by Ross *et al.*^16^

This project aimed to build on the concepts mentioned above and combine them into a single construct of a cleavable peptide–drug conjugate (Fig. 1d). The peptide component would provide selectivity for the bacteria while also disrupting their cell membrane. The secondary structure of the peptide would be reinforced by a staple to aid the mechanism of action *via* restricting the conformational entropy. The small molecule component, namely ciprofloxacin, would provide an orthogonal antibacterial mode of action, hence reducing the chance of resistance forming. Finally, the peptide and the drug would be connected *via* a cleavable linker using a β-lactam moiety so that the construct would be active only in the presence of bacteria that would otherwise be resistant to treatment with β-lactam antibiotics. The primary target organism used for the evaluation of this study was *P. aeruginosa* due to its abundant resistance pathways and high research priority.^17^

## 2 Results and discussion

### 2.1 Peptide selection

With the bacterial target in mind, we started by looking for a peptide sequence that would provide selectivity for *P. aeruginosa*. Seven peptide sequences were obtained from literature that represent naturally occurring antimicrobial peptides from a variety of species (Table 1). Most of the chosen peptides showed inhibitory activity against *P. aeruginosa* and one of them (P1) had been previously stapled with a hydrocarbon staple. The positions for insertion of cysteine residues were chosen to allow *i*, *i* + 7 stapling and to minimally perturb the cationic and amphipathic character of the peptides.

**Table 1:** Sequences of peptides selected for initial screen. PXa is the parent sequence of peptide X without cysteine residues, PX stands for the sequence of peptide *X* with cysteine residues inserted for stapling and PX-1 is peptide PX stapled with staple 1, represented by bold cysteine residues^a^

Pep	Sequence	Inh^b^	Origin/ref.
P1a	Ac–TLKQFAKGVGKWLVK–NH_2_	—	frog^11,18^
P1-1	Ac–TLKQF**C**KGVGKW**C**VK–NH_2_	✗	
P2a	H–FAKWAFKWLKK–NH_2_	✗	frog^19^
P2	H–FCKWAFKWCKK–NH_2_	✗	
P2-1	H–F**C**KWAFKWCKK–NH_2_	✓	
P3a	Ac–WMLKKFRGMF–NH_2_	—	bact.^20^
P3-1	Ac–W**C**LKKFRG**C**F–NH_2_	✗	
P4a	H–RFRRLRKKWRKRLKKI–NH_2_	✓	pig^21^
P4	H–RFRRLCKKWRKRCKKI–NH_2_	✗	
P4-1	H–RFRRLCKKWRKR**C**KKI–NH_2_	✓	
P5a	C_8_–GLLKFIKKLL–NH_2_	—	wasp^22^
P5-1	C_8_–G**C**LKFIKK**C**L–NH_2_	✗	
P6a	H–KWVQNYMKHLGRKAHTLKT–NH_2_	✓	human^23^
P6	H–KWVQNYCKHLGRKCHTLKT–NH_2_	✗	
P6-1	H–KWVQNY**C**KHLGRK**C**HTLKT–NH_2_	✓	
P7a	H–NLFRKLTHRLFRRNFGYTLR–NH_2_	—	human^24^
P7-1	H–NLFRKL**C**HRLFRRCFGYTLR–NH_2_	✗	

a Abbreviations: *Pep* peptide, *Inh* inhibition, *bact*. bacterium.

b Inhibition measured at 256 μg mL^−1^ of peptide after overnight incubation of *Pseudomonas aeruginosa* strain PAO1. Tick (✓) represents OD_600_ comparable to no-growth control, cross (✗) represents OD_600_ comparable to growth control without any peptide, dash (—) means inhibition was not measured for the peptide.

The peptides were synthesised using solid-phase peptide synthesis and were tested for their initial properties of inhibiting the growth of *P. aeruginosa* in their stapled forms. The peptides were stapled with a simplified staple 1, which had been synthesised using a procedure previously used in the group (Scheme S1a and b). The stapling was performed in a 3 : 1 mixture of DMF and 2-(*N*-morpholino)ethanesulfonic acid (MES) buffer (50 mm, pH 6) as the peptide showed worse solubility when basic buffer was used (sodium phosphate buffer, 50 mm, pH 8) and the staple showed poorer solubility when a low proportion of organic solvent was used.

Of the tested peptides, three (P2, P4 and P6) showed inhibition of cell growth when tested initially at a fixed concentration (256 μg mL^−1^). For these peptides, the unstapled versions and parent sequences without cysteine insertion were subsequently also tested at the fixed concentration. Peptide P2 stood out as its stapled version P2-1 showed improvement in activity and P2 was therefore used for further studies.

**Fig. 2:**
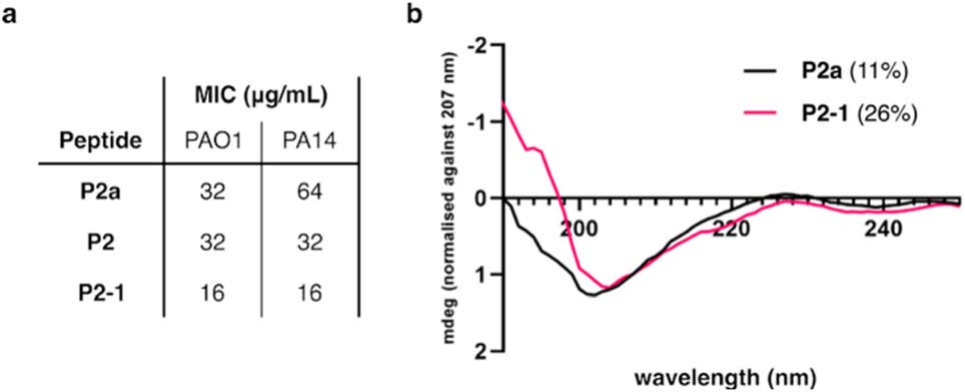
Further analysis of peptide P2. (a) Minimal inhibitory concentration (MIC) for the peptide P2a, its analogue with cysteine residues (P2) and the stapled peptide (P2-1). Measured for *P. aeruginosa* strains PAO1 and PA14. (b) The circular dichroism (CD) spectra of P2-1 and P2a in MeCN/H_2_O (1 : 1). The percent helicity is given in parentheses in the legend.

The minimal inhibitory concentration (MIC) was measured for peptide P2a, its variant with inserted cysteine residues (P2) and P2 stapled with model staple 1 (P2-1). Two strains of *P. aeruginosa* were used for this, reference strain PAO1 and a more virulent strain PA14.^25^ When we introduced a staple into the structure of the peptide, the MIC dropped (Fig. 2a). Indeed, this was expected as membrane-acting peptides often need to organise into a secondary structure before they act on the membrane.^5^ In the context of this project, stapling was intended to pre-organise the peptide in a helical conformation. Circular dichroism (CD) data confirmed increased α-helicity of the stapled peptide (Fig. 2b).

**Fig. 3:**
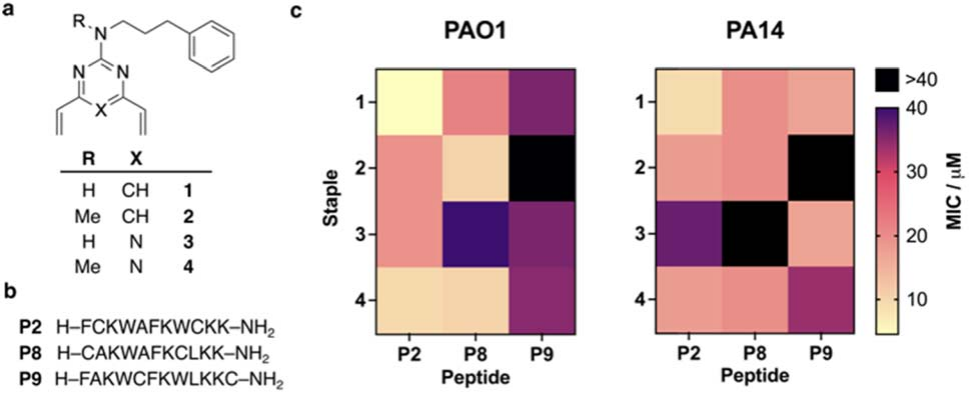
A study to establish the influence of the staple core on the activity of the stapled AMPs. (a) Staples based on triazine or pyrimidine, with or without N–Me were prepared. (b) Two additional peptides with staple on the hydrophobic face of the helix were also prepared. (c) Heat map showing the MIC of the different peptide-staple combinations.

In addition, the effect of staple core on the MIC was investigated. The Spring Group previously reported both divinylpyrimidines^13^ and divinyltriazines^12^ as peptide staples and thus there was interest to ascertain if this subtle chemical change in the staple core can have an effect on the stapled peptide's antibacterial activity. Hence, the use of divinylpyrimidine, divinyl triazine and methylation of the appended amine were tested for their impact on peptide stapling and activity (Fig. 3a). Two more stapling positions on the hydrophobic side of the peptide helix were investigated at the same time in P8 and P9 (Fig. 3b). Of these, the original stapled peptide P2-1 displayed the best activity, confirming that the initial scaffold was most suitable to be a starting point for further elaboration (Fig. 3c). Finally, with the knowledge that the drug released will be ciprofloxacin, peptide P2a was characterised for potential synergy with the small-molecule drug, however only an additive effect was observed (fractional inhibitory concentration index was established to be 0.75, which is above the synergy threshold of 0.5). This suggests that in a mixture the two compounds need to be present at their respective MIC concentrations to be active.

### 2.2 Conjugate synthesis

**Scheme 1:**
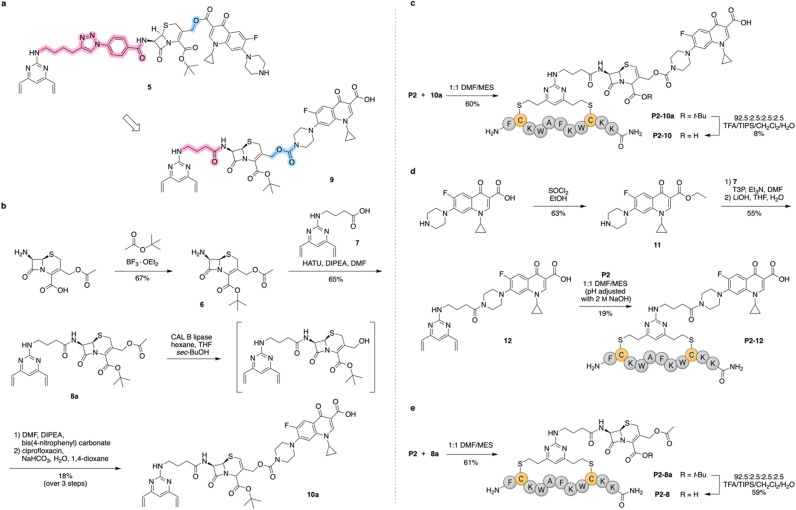
(a) The initial functionalised staple design and the updated design. The changes between the two designs are highlighted. The linker between cephalosporin and divinylpyrimidine is shown in pink and connection between cephalosporin and ciprofloxacin is shown in blue. (b) Synthesis of the second generation staple 10a. Compound 7 was prepared using a previously reported procedure (Scheme S1c).^13^ (c) Stapling of peptide with staple and removal of the *t*-Bu protecting group. (d) Preparation of the non-cleavable stapled peptide–ciprofloxacin conjugate. (e) Preparation of a peptide stapled with cephalosporin only, acting as a control.

Having selected a suitable stapled antimicrobial peptide, we moved on to synthesise the fully functionalised staple bearing the cleavable linker and ciprofloxacin prodrug. The initial design of the functionalised staple (Scheme 1a) was inspired by the work of Evans *et al.*,^15^ relying on the direct attachment of the ciprofloxacin carboxylate to an azide-bearing cephalosporin. The azide would be used for attachment of divinylpyrimidine using click chemistry to make the synthesis more modular. However, synthetic challenges encountered during the attempted synthesis of 5 resulted in a modified design 9. This updated design employed a carbamate moiety to attach ciprofloxacin to the cephalosporin and contained an aliphatic amide on the C-7 amine of cephalosporin in place of the aromatic amide. The latter feature was intended to improve cleavage by AmpC, a type of β-lactamase expressed by *P. aeruginosa*.^15^ In the end, an isomer of the new linker (10a) was synthesised following the pathway in Scheme 1b. This isomer where the cephalosporin double bond migrated is reported to show some activity,^26^ hence the compound was employed for peptide stapling.

Due to the need of acidic conditions to remove the *t*-Bu protecting group and the sensitivity of divinylpyrimidine to acid, the deprotection step was planned to come after the stapling reaction. Therefore, linker 10a was used for the stapling of peptide P2. This was performed in a 1 : 1 mixture of DMF and MES buffer (50 mm, pH 6). The slightly lower proportion of DMF in the mixture resulted in faster stapling completion so these conditions were used for all further stapling reactions. Under these conditions the stapling achieved full conversion (monitored by HPLC) in under two hours.

The subsequent *t*-Bu deprotection was synthetically challenging. Several literature conditions were trialled, including 4 m HCl in 1,4-dioxane,^27^ TMSOTf in dichloromethane,^28^*Bacillus subtilis* esterase^29^ and mixtures of trifluoroacetic acid with dichloromethane and carbocation scavengers such as anisole,^15^ thioanisole,^30^ dimethoxybenzene^31^ and silanes. None gave a clean cleavage product. The most promising set of conditions was the standard peptide deprotection conditions (92.5 : 2.5 : 2.5 : 2.5 TFA/CH_2_Cl_2_/H_2_O/TIPS),^13^ where product formation was observed using liquid chromatography–mass spectrometry (LC–MS), but merely as a minor product. Conjugate cleavage at the carbamate was observed and attributed to the reducing capability of silanes (Scheme S2). Different amounts of TIPS and different silanes were trialled in an attempt to improve the deprotection step (Table S1). However, the standard peptide deprotection conditions were eventually used for the *t*-Bu deprotection since they reproducibly gave the desired product P2-10 albeit at an overall yield for stapling and deprotection of 5% (Scheme 1c). Introduction of other protecting groups (allyl, methyl, benzhydryl) to avoid this issue was attempted, but only with *t*-Bu group were we able to synthesise the full staple (Table S2). The stapled peptide prior to *t*-Bu deprotection (P2-10a) was also tested for its antibacterial properties in an attempt to avoid the low-yielding deprotection, however the activity was four-fold lower than for the deprotected conjugate (Table S3).

In addition to the target conjugate P2-10, two control compounds (P2-12 and P2-8) were also synthesised (Scheme 1d and e). Compound P2-12 acted as a non-cleavable peptide–ciprofloxacin conjugate, aiming to show the benefit of a cleavable linker. Compound P2-8 represented the stapled peptide released after the cleavage of the cephalosporin linker and it was used to understand the contribution of the peptide to the overall activity of the conjugate.

### 2.3 Biological testing

After the synthesis of the desired conjugates, we moved on to the evaluation of their properties. First, their MIC values for *P. aeruginosa* were measured (Table 2). Like in the peptide selection process, *P. aeruginosa* strains PAO1 and PA14 were used. The lack of activity for the non-cleavable conjugate P2-12 was consistent with no release of ciprofloxacin. The stapled peptide control P2-8 disappointingly also showed no activity, which was unexpected as the same peptide showed antimicrobial activity when stapled with a simplified staple 1 (MIC of 8–16 μg mL^−1^, 4.6–9.2 μm; P2-1). This was attributed to be likely due to the different structure of staple 8 compared to staple 1. Cleavable conjugate P2-10 showed an increased level of activity compared to the controls, but the MIC did not reach the expected values, potentially due to limited cleavage of ciprofloxacin from the conjugate. It was envisaged that following complete cleavage of conjugate P2-10 the MIC values (expressed in μm) would reach the same level as ciprofloxacin alone.

**Table 2:** Minimal inhibitory concentration (MIC) for peptide P2-10 and the control compounds

Compound	MIC for PAO1		MIC for PA14	
	μg mL^−1^	μm	μg mL^−1^	μm
P2-12	> 64	> 32	> 64	> 32
P2-8	> 64	> 33	> 64	> 33
P2-10	16	7.0	16	7.0
Ciprofloxacin	0.125	0.38	0.0625	0.19
Polymyxin B	2	1.7	1	0.83

More biological experiments were carried out to rationalise the observed trends. Firstly, the difference in activity between peptide stapled with cephalosporin-based staple (P2-8) and the peptide stapled with the simplified staple (P2-1) was investigated. Due to the presence of polar functional groups such as carboxylic acid and amide, staple 8 is expected to be overall more polar than simplified staple 1. Therefore, the positioning of staple 8 on the hydrophobic surface of the peptide could be disrupting the amphipathic nature of the peptide necessary for activity of the AMP. If this were the case, simplified staple 1, being more hydrophobic, like literature hydrocarbon staples, would be better tolerated on the hydrophobic surface of the peptide. To test this hypothesis, a series of peptides with staple 8 introduced at different positions, including positions on the hydrophilic surface of the peptide, was created but none of the variants showed improvement over the original peptide P2-8 (Fig. S1 and Table S4).

**Fig. 4:**
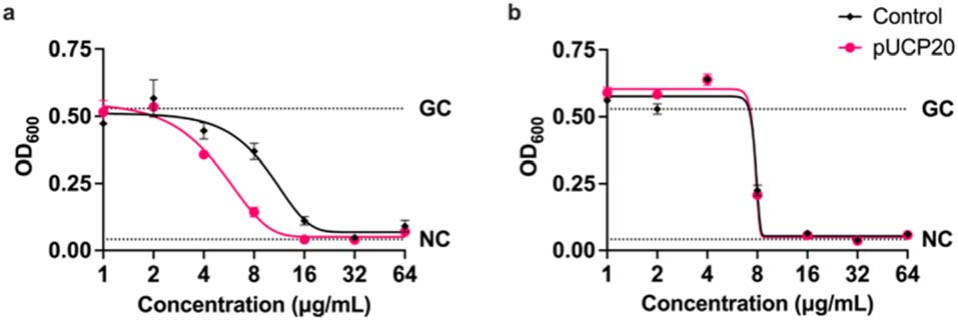
Curve showing growth of bacteria measured by OD_600_ after incubation at different concentrations of P2-10 (a) or P2a (b). Each point was done in triplicate; error bars represent standard deviation. NC stands for negative control (incubated without added bacteria, serves as sterility control); GC stands for growth control (incubated without any drugs). Tested on strain PAO1 with and without plasmid pUCP20.

Next, we focused on the apparent incomplete cleavage of the conjugate. In order to further our understanding, the experiments primarily focused on the availability of β-lactamase for cleavage. *P. aeruginosa* is known to express higher levels of β-lactamase AmpC, whose substrates include cephalosporins, in the presence of β-lactams.^32^ The growth medium was hence spiked with sub-MIC amount of cephalosporin ceftazidime, but this did not cause a reduction in the MIC (Table 3). Following this, an alternative way of increasing β-lactamase levels was attempted by using a PAO1 strain containing plasmid pUCP20, which encodes a β-lactamase. This strain showed a slight, but statistically significant decrease in cell growth around the MIC value, which resulted in a reduction in MIC by one half (Fig. 4 and Table 3). Pleasingly, no difference in cell growth inhibition was observed for a control peptide P2a in the strain containing the plasmid.

**Table 3:** Minimal inhibitory concentration (MIC) for peptide P2-10 under different incubation conditions. Data combined from multiple experiments, each containing ciprofloxacin and polymyxin B controls

Compound	MIC for PAO1		MIC for PA14	
	μg mL^−1^	μm	μg mL^−1^	μm
P2-10	32	14	16	7.0
P2-10 + 0.25 μg mL^−1^ ceftazidime	32	14	32	14
P2-10 + plasmid pUCP20	16	7.0	N/A	N/A
Ceftazidime	1	1.8	2	3.7
Ciprofloxacin	0.125	0.38	0.0625	0.19
Polymyxin B	2	1.7	1	0.83

This differential activity between the two variants of *P. aeruginosa* prompted us to expand the testing to other bacterial species. A mixture of strains with infectious disease relevance were selected, for example including a multi-resistant clinically-derived strain of *Klebsiella pneumoniae*, another high-priority pathogen.^33^ Two general trends appeared in the MIC data (Table 4). Firstly, cleavable conjugate P2-10 usually had a lower MIC than the parent peptide and the cephalosporin-stapled peptide without ciprofloxacin (P2a and P2-8, respectively). Peptide P2-8 also usually had a higher MIC than P2a, similar to the trend observed for *P. aeruginosa*. Secondly, the amount by how much the activity of P2-10 exceeded that of P2-8 matched the relative susceptibility to ciprofloxacin between the different bacteria. The only outlier to this was *Enterococcus faecalis*, which showed improvement of MIC on stapling with the cephalosporin staple, but the MIC worsened on stapling with the cephalosporin-ciprofloxacin staple. To test whether this could have been a result of potential antagonism of P2-8 and free ciprofloxacin, an MIC for a 1 : 1 mixture of the two compounds was measured, but the activity was equivalent to ciprofloxacin alone. Overall, conjugate P2-10 unfortunately did not show a higher activity than ciprofloxacin alone for any of the additional strains. Having said that, the results demonstrated that the conjugate, and the peptide component itself, can have differential activity towards different bacterial strains. To further demonstrate this selectivity, compound P2-10 was tested against mammalian HEK293FT cells and in the range covering the observed MICs against bacteria (1–128 μg mL^−1^), the mammalian cells showed a consistent viability over 90% (Fig. S2).

**Table 4:** MIC values for compounds P2a, P2-8, P2-10 and ciprofloxacin (cipro), measured on six additional bacterial strains

Strain	MIC (μg mL^−1^)			
	P2a	P2-8	P2-10	Cipro
*Escherichia coli* DH5a	4	32	1	0.0078
*Klebsiella pneumoniae* CK1	≥ 64	≥ 64	≥ 64	≥ 1
*Burkholderia thailandensis* E264	≥ 64	≥ 64	≥ 64	1
*Serratia marcescens* ATCC39006	≥ 64	≥ 64	16	0.0625
Methicillin-resistant *Staphylococcus aureus* MRSA15	32	64	8	0.125
*Enterococcus faecalis* ATCC29212	≥ 64	2	16	0.5

To better understand the involvement of β-lactamase in cleavage of the conjugate, we decided to assess the amount of the enzyme produced by the bacteria. The assays were based on cephalosporin-based nitrocefin dye. Initially, bacteria were co-incubated with the dye and the absorption at the nitrocefin-specific wavelength of 482 nm (Abs_482_) was measured throughout the incubation period. By comparing the rate of increase of Abs_482_ (adjusted for light scattering due to growing bacteria) with the rate of growth, as monitored by OD_600_, we were hoping to establish the rate of β-lactamase production. However, the dye itself inhibited growth of some bacteria and for others the curve did not fit to the expected sigmoidal shape (Fig. S3). Additionally, the growth rate and lag time parameters obtained from the growth curves did not seem to correlate with the MIC values (Fig. S4). As an alternative method to assay the production of β-lactamase, samples of liquid culture were taken at several time points throughout the incubation period and the initial rate of hydrolysis of the nitrocefin dye by the cell-free medium measured (Fig. S5). Using the calibration curves for nitrocefin and β-lactamase (Fig. S6), the amount of the recombinant β-lactamase with equivalent activity could be estimated. While the assay did not report any significant change in nitrocefin hydrolysis over the time of incubation for methicillin-resistant *Staphylococcus aureus* (MRSA) and *E. faecalis*, it did show increase in the hydrolysis rate for the other strains with increasing time, suggesting the amount of β-lactamase produced to be proportional to the bacterial population. However, the rise in nitrocefin hydrolysis rate usually started to be noticeable at around 5 h after the start of the incubation, which is about 3 h after the bacteria enter exponential growth phase. The hydrolysis rates from the final reading after 21.5 h of incubation are summarised in Table 5. The values observed for *P. aeruginosa* further support the differential results observed earlier (Fig. 4) as when the bacterium contains plasmid pUCP20, it shows higher β-lactamase production, which can justify the higher susceptibility to P2-10.

**Table 5:** Rate of nitrocefin hydrolysis by β-lactamase in a cell-free medium sample taken from bacterial cultures after 21.5 h of incubation, and equivalent amount of recombinant β-lactamase capable of achieving the same rate

Strain	Rate of hydrolysis [nm s^−1^]	β-lactamase equivalent [pm]
*Pseudomonas aeruginosa* PAO1	4.6	45.7
*Pseudomonas aeruginosa* PAO1 (pUCP20)	44.6	442.9
*Escherichia coli* DH5a	5.8	57.6
*Klebsiella pneumoniae* CK1	155.5	1544.2
*Burkholderia thailandensis* E264	10.1	100.3
*Serratia marcescens* ATCC39006	6.4	63.6

With the knowledge of approximate amounts of β-lactamase that can be observed in the growth medium during bacterial incubation, we moved on to study the cleavage of P2-10 by the recombinant enzyme using an HPLC. Initially, 0.125 nm of the enzyme was chosen as the amount should give similar level of nitrocefin cleavage as was observed at the end of incubation of *P. aeruginosa* supplemented with pUCP20 plasmid and this amount should also give enough time points for the time scale of HPLC measurements. However, at that concentration of β-lactamase the hydrolysis rate of P2-10 was significantly below what was observed for nitrocefin at the same enzyme concentration. Thus, the amount of β-lactamase was increased to 1 nm, resulting in a gradual cleavage of the conjugate to a full release of ciprofloxacin after over 24 h. The rate was still significantly lower than that observed for nitrocefin at the same concentration of the enzyme (full hydrolysis achieved after about 20 min; Fig. S6b), but other studies have also seen a less efficient β-lactam hydrolysis for different cephalosporin derivatives.^26^

**Fig. 5:**
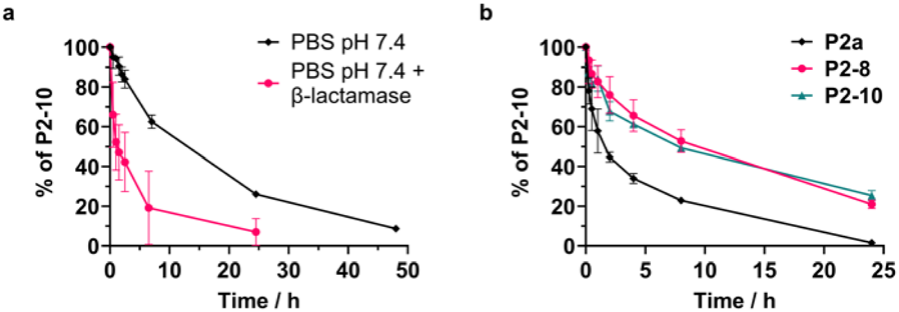
(a) *In vitro* cleavage of P2-10 by 1 nm recombinant β-lactamase compared to the background stability in PBS buffer, pH 7.4. (b) Serum stability of peptides P2a, P2-8 and P2-10.

Background hydrolysis rate mediated by buffer only was measured as a control and was significantly lower than when β-lactamase was present (Fig. 5a). The background rate of hydrolysis was also not too dissimilar to values observed for other cephalosporins. To add to the characterisation of P2-10, serum stability was assessed (Fig. 5b). As expected, stapled peptides (P2-10 as well as cephalosporin-only stapled P2-8) showed an improved stability over unstapled P2a. Pleasingly, the serum stability of the cleavable conjugate P2-10 was similar to that of P2-8, suggesting that there is no decrease in serum stability due to the cleavable linker.

Finally, seeing the small β-lactamase concentrations in the growth media and the slower release kinetics of ciprofloxacin, we wanted to confirm whether the effects of conjugate P2-10 could be enhanced by supplementing the medium with β-lactamase, hence increasing the rate of ciprofloxacin release. We were pleased to observe that after adding 1 nm of the recombinant β-lactamase, the MIC values for P2-10 (2 μg mL^−1^, 0.88 μm for PAO1) were of the same order of magnitude as ciprofloxacin (0.125 μg mL^−1^, 0.38 μm), consistent with full release of ciprofloxacin.

## 3 Conclusions

In this work we exemplified how a more convergent two-component peptide stapling can be used to access an antimicrobial peptide–drug conjugate with a novel mechanism of action. Our optimised construct comprised a stapled peptide (sequence of H–FCKWAFKWCKK–NH_2_), a cleavable linker and ciprofloxacin. Peptide stapling, introducing a handle for linker attachment, was shown to affect the physico-chemical properties of the peptide such as its antimicrobial activity and propensity to adopt a helical conformation. Stapling also improved serum stability of the peptide and it was the peptide's stability that was the limiting factor for the stability of P2-10 in serum as introduction of the cleavable linker did not make the rate of degradation faster. Addition of β-lactamase into the mixture increased the rate of release of ciprofloxacin from P2-10 above the spontaneous hydrolysis/degradation rate. This catalysed rate was still about 10 times lower than what was observed for nitrocefin dye in β-lactamase assessment, which highlights the potential for further optimisations. Simple extension of the linker on the peptide side to relieve potential steric congestion around cephalosporin does not yield an improved MIC (Table S5) and hence a more detailed study is needed. In addition, a further delay in the onset of the effect of P2-10 could be also caused by the slow gradual increase in the β-lactamase concentration that is below the bacterial growth rate, therefore requiring more ciprofloxacin to be released before the bacterial growth is hindered.

Pleasingly, conjugate P2-10 showed differential activity across a panel of bacteria. The results suggest that the overall efficiency of the conjugate is a composite of the efficiency of the peptide, drug and the cleavable linker. When two of the three had similar efficiency against given bacteria, the efficiency of the whole conjugate generally tended to be in line with the efficiency of the third component of the conjugate. For example, the MIC for P2-10 was in line with the rate of hydrolysis between *P. aeruginosa* with and without plasmid pUCP20, with the two strains having similar values of MIC for the peptide and ciprofloxacin alone. Alternatively, the MIC of P2-10 for *Burkholderia thailandensis* and *Serratia marcescens* were in line with the strains' susceptibility to ciprofloxacin, while the two strains shared similar rate of hydrolysis of cephalosporins and MIC of the peptide component. Notably, in the case of *K. pneumoniae*, which showed high MIC towards the peptide and resistance towards ciprofloxacin, a different peptide-drug combination would be more suitable to leverage the high level of cephalosporin hydrolysis of the tested strain. This highlights the need for careful selection of the components in the case of multidrug resistant strains, but a good combination of components can lead to good levels of selectivity. Therefore, whilst further optimisation of P2-10 is necessary, this work provides the basis for the development of antibacterial drug conjugates that would simultaneously allow for selectivity and multimodal mechanism of action and therefore overcome antimicrobial resistance.

## Author contributions

J. S. G. and D. R. S. were involved in conceptualisation of this study. T. D., J. S. G. and B. H. G. were involved in chemical and biological investigation. K. A. K. investigated the mammalian toxicity. T. J. O. contributed to the biological investigation. Y. S. T. performed computational analysis of peptide stapling. T. D. performed visualisation of the results and writing of the original draft. D. R. S. (synthesis), M. W. (bacterial testing) and J. S. C. (mammalian testing) provided resources for the study. D. R. S., M. W., J. S. C, T. A. H. and J. S. P. were involved in supervision. All authors reviewed and edited the manuscript.

## Conflicts of interest

There are no conflicts to declare.

## Supplementary Material

SC-016-D5SC06369H-s001

## Data Availability

The data supporting this article have been included as part of the SI. See DOI: https://doi.org/10.1039/d5sc06369h.

## References

[cit1] Hutchings M. I., Truman A. W., Wilkinson B. (2019). Curr. Opin. Microbiol..

[cit2] O'Neill J. , Tackling drug-resistant infections globally: final report and recommendations, The Review on Antimicrobial Resistance, HM Government and Wellcome Trust, 2016

[cit3] The PEW Charitable Trusts , A Scientific Roadmap for Antibiotic Discovery, The PEW Charitable Trusts, 2016

[cit4] World Health Organization , Global research agenda for antimicrobial resistance in human health, World Health Organization, 2024

[cit5] Gan B. H., Gaynord J., Rowe S. M., Deingruber T., Spring D. R. (2021). Chem. Soc. Rev..

[cit6] Dijksteel G. S., Ulrich M. M. W., Middelkoop E., Boekema B. K. H. L. (2021). Front. Microbiol..

[cit7] Koo H. B., Seo J. (2019). Pept. Sci..

[cit8] Lau Y. H., De Andrade P., Wu Y., Spring D. R. (2015). Chem. Soc. Rev..

[cit9] Iegre J., Gaynord J. S., Robertson N. S., Sore H. F., Hyvönen M., Spring D. R. (2018). Adv. Ther..

[cit10] Migoń D., Neubauer D., Kamysz W. (2018). Protein J..

[cit11] Pham T. K., Kim D.-H., Lee B.-J., Kim Y.-W. (2013). Bioorg. Med. Chem. Lett..

[cit12] Robertson N. S., Walsh S. J., Fowler E., Yoshida M., Rowe S. M., Wu Y., Sore H. F., Parker J. S., Spring D. R. (2019). Chem. Commun..

[cit13] Walsh S. J., Iegre J., Seki H., Bargh J. D., Sore H. F., Parker J. S., Carroll J. S., Spring D. R. (2020). Org. Biomol. Chem..

[cit14] Liu R., Miller P. A., Vakulenko S. B., Stewart N. K., Boggess W. C., Miller M. J. (2018). J. Med. Chem..

[cit15] Evans L. E., Krishna A., Ma Y., Webb T. E., Marshall D. C., Tooke C. L., Spencer J., Clarke T. B., Armstrong A., Edwards A. M. (2019). J. Med. Chem..

[cit16] Ross C. L., Lawer A., Sircombe K. J., Pletzer D., Gamble A. B., Hook S. (2024). J. Med. Chem..

[cit17] World Health Organization , WHO Bacterial Priority Pathogens List 2024: Bacterial Pathogens of Public Health Importance, to Guide Research, Development, and Strategies to Prevent and Control Antimicrobial Resistance, World Health Organization, Geneva, 1st edn, 2024

[cit18] Park J. M., Jung J.-E., Lee B. J. (1994). Biochem. Biophys. Res. Commun..

[cit19] Won H.-S., Kang S.-J., Choi W.-S., Lee B.-J. (2011). Mol. Cells.

[cit20] Saikia K., Sravani Y. D., Ramakrishnan V., Chaudhary N. (2017). Sci. Rep..

[cit21] Zhu X., Shan A., Ma Z., Xu W., Wang J., Chou S., Cheng B. (2015). Antimicrob. Agents Chemother..

[cit22] Chionis K., Krikorian D., Koukkou A.-I., Sakarellos-Daitsiotis M., Panou-Pomonis E. (2016). J. Pept. Sci..

[cit23] Cossio-Ayala M., Domínguez-López M., Mendez-Enriquez E., Portillo-Téllez M. D. C., García-Hernández E. (2017). Peptides.

[cit24] Papareddy P., Kasetty G., Kalle M., Bhongir R. K. V., Mörgelin M., Schmidtchen A., Malmsten M. (2016). J. Antimicrob. Chemother..

[cit25] Mikkelsen H., McMullan R., Filloux A. (2011). PLoS One.

[cit26] Frere J.-M., Kelly J. A., Klein D., Ghuysen J.-M., Claes P., Vanderhaeghe H. (1982). Biochem. J..

[cit27] Yanagisawa H., Amemiya Y., Kanazaki T., Shimoji Y., Fujimoto K., Kitahara Y., Sada T., Mizuno M., Ikeda M., Miyamoto S., Furukawa Y., Koike H. (1996). J. Med. Chem..

[cit28] Sharp L. A., Zard S. Z. (2006). Org. Lett..

[cit29] Schmidt M., Barbayianni E., Fotakopoulou I., Höhne M., Constantinou-Kokotou V., Bornscheuer U. T., Kokotos G. (2005). J. Org. Chem..

[cit30] Koteva K. P., Cantin A. M., Neugebauer W. A., Escher E. (2001). Can. J. Chem..

[cit31] Schmidt U., Lieberknecht A., Boekens H., Griesser H. (1983). J. Org. Chem..

[cit32] Torrens G., Hernández S. B., Ayala J. A., Moya B., Juan C., Cava F., Oliver A. (2019). mSystems.

[cit33] Dinda V., Kimang’a A. N., Kariuki D., Sifuna A. W., O'Brien T. J., Welch M., Reva O. N. (2024). Access Microbiol..

