# Supplementary material for: β-Lactamase cleavable antimicrobial peptide–drug conjugates
Source: Chem Sci. 2025 Sep 19;16(41):19288–95. doi: 10.1039/d5sc06369h (PMC12447755; doi:10.1039/d5sc06369h)
Supplement: SC-016-D5SC06369H-s001 [file SC-016-D5SC06369H-s001.pdf]

Supplementary Information (SI) for Chemical Science.
This journal is © The Royal Society of Chemistry 2025

SUPPLEMENTARY INFORMATION

β-Lactamase cleavable antimicrobial peptide-drug conjugates

Tomas Deingruber,[a] Josephine S. Gaynord,[a] Bee Ha Gan,[a] Kristina A. Kostadinova,[a] Thomas J. O'Brien,[b] Yaw Sing Tan,[c] Jeremy S. Parker,[d] Thomas A. Hunt,[e] Jason S. Carroll,[f] Martin Welch,*[b] David R. Spring*[a]

Contents

1 Supplementary schemes and figures 1
2 Supplementary tables 8
3 General information 13
4 Experimental procedures 16
5 Peptide synthesis and analysis 30
6 Biological assays 40
7 NMR spectra 45
8 HPLC traces 69
9 References 86

* *Corresponding authors, E-mail: mw240@cam.ac.uk, spring@ch.cam.ac.uk*
[a] *Yusuf Hamied Department of Chemistry, University of Cambridge, Lensfield Road, Cambridge, UK.*
[b] *Department of Biochemistry, University of Cambridge, Hopkins Building, Tennis Court Road, Cambridge, UK.*
[c] *Bioinformatics Institute, Agency for Science, Technology and Research (A*STAR), Singapore.*
[d] *Early Chemical Development, Pharmaceutical Development, R&D, AstraZeneca, Macclesfield, UK.*
[e] *Oncology R&D, AstraZeneca, Cambridge, UK.*
[f] *Cancer Research UK Cambridge Institute, Robinson Way, Cambridge, CB2 ORE, UK.*

1 Supplementary schemes and figures

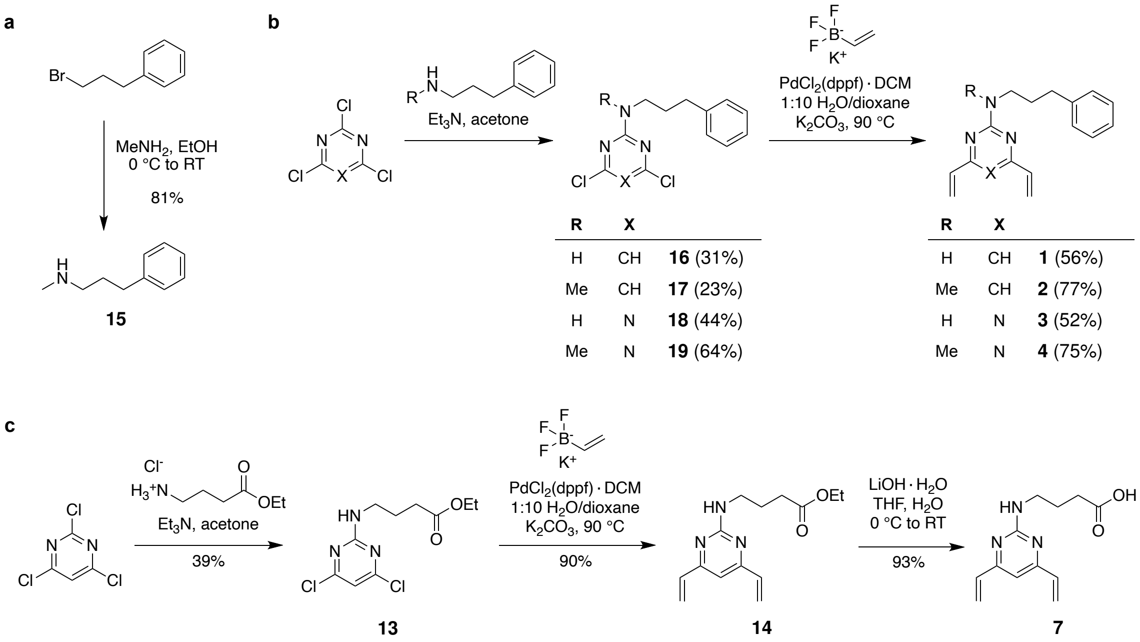

Scheme S1 Synthesis of divinylazines.

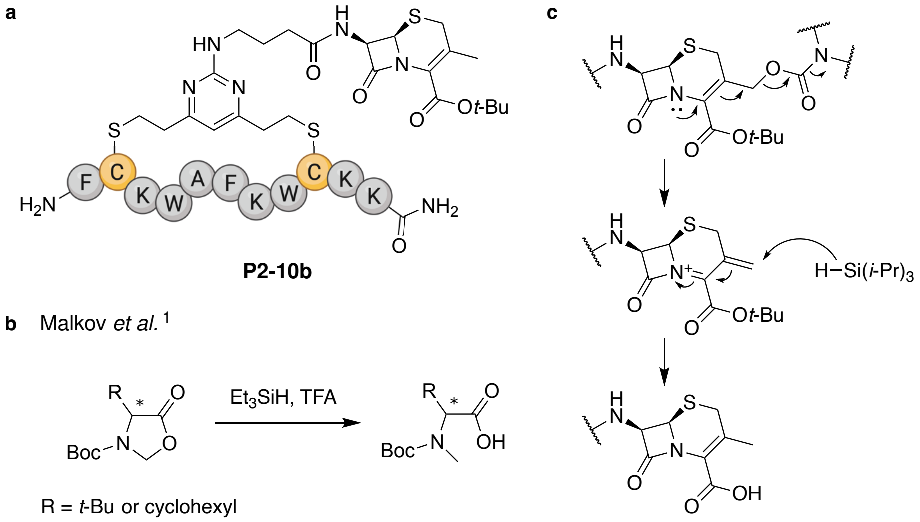

Scheme S2 **a** Silane reduction by-product observed during deprotection using ($TFA/TIPS/H_2O/CH_2Cl_2$, 92.5:2.5:2.5:2.5). **b** Example of a reported silane-mediated reduction resembling the one observed.[1] Chiral centre marked by an asterisk. **c** Potential mechanism of the silane-mediated reduction.

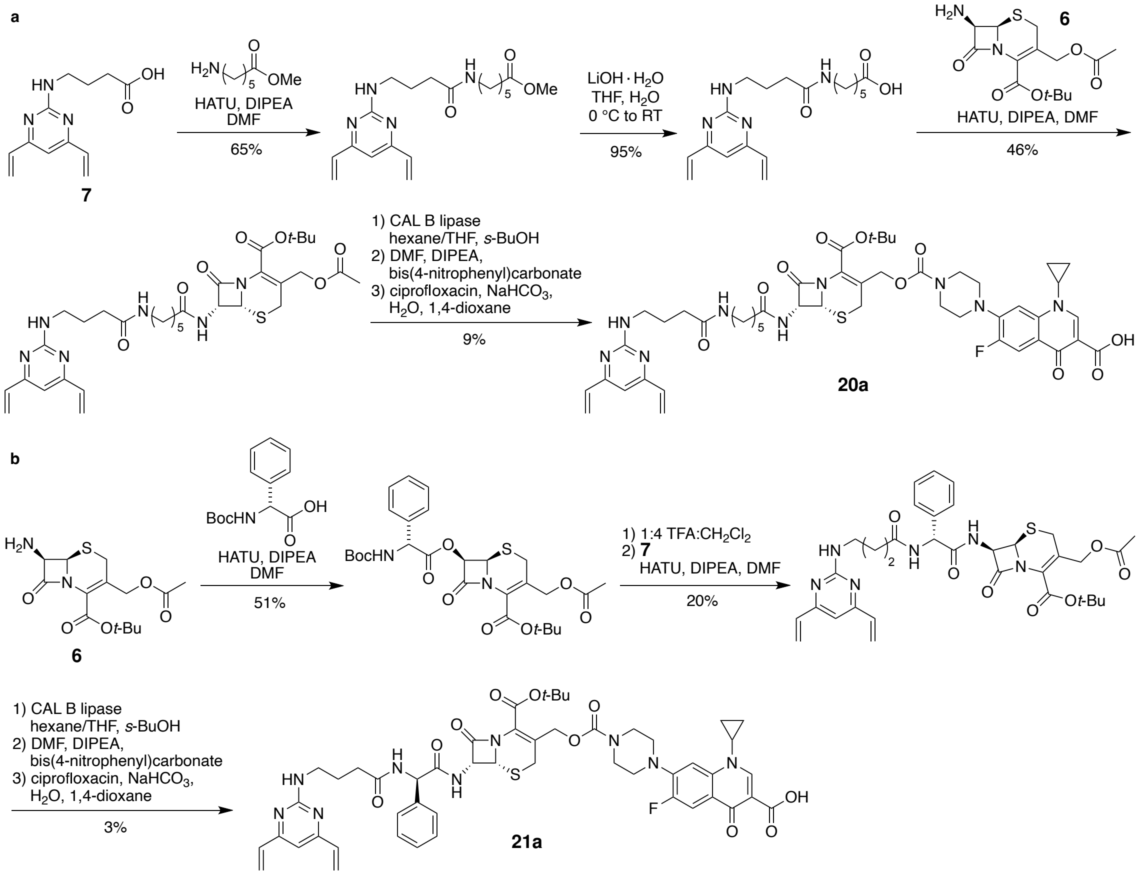

Scheme S3 Synthetic pathways to staples with extended linker between the divinyl pyrimidine moiety and cephalosporin.

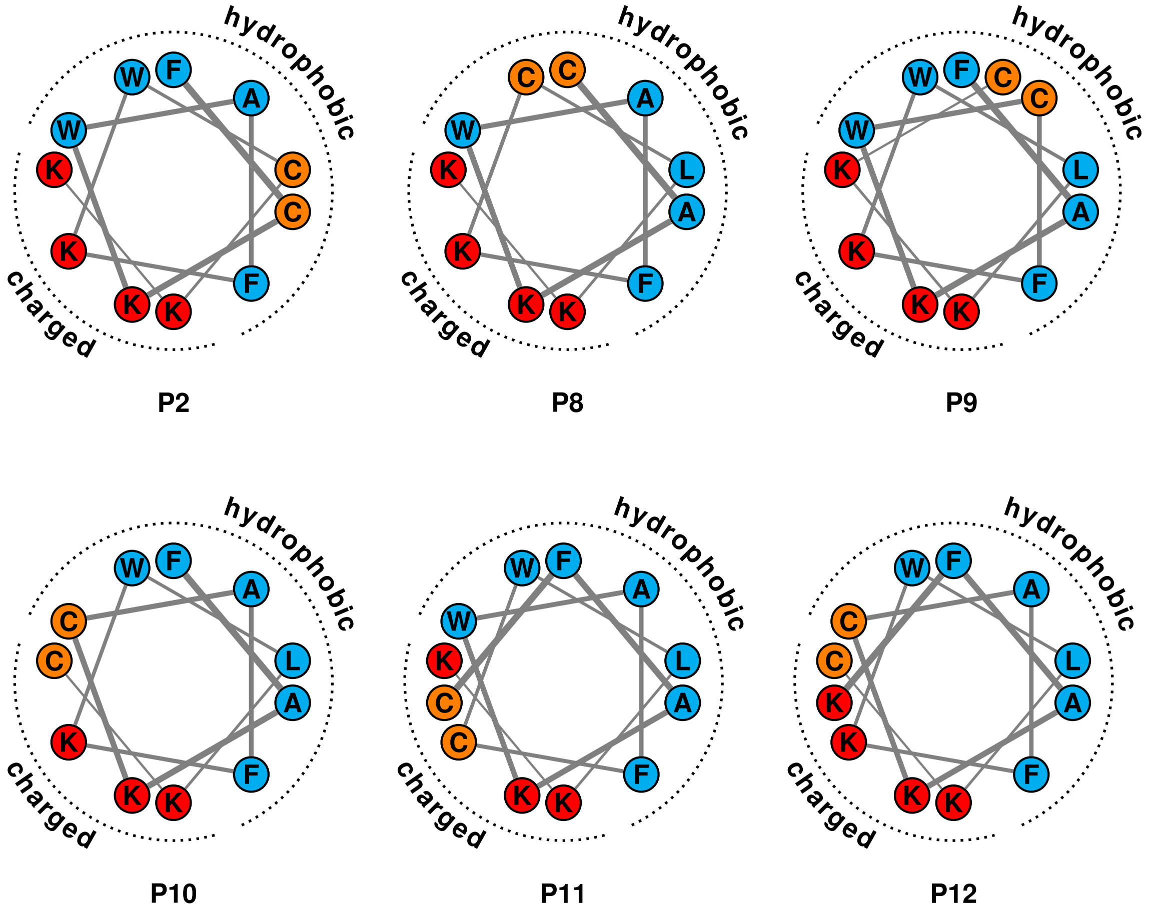

Figure S1 Helix pinwheel representation of the peptides derived from peptide **P2** with varying position of cysteine residues for stapling. Hydrophobic residues are shown in blue and positively-charged, hydrophilic residues in red. Cysteine residues used for stapling are shown in orange.

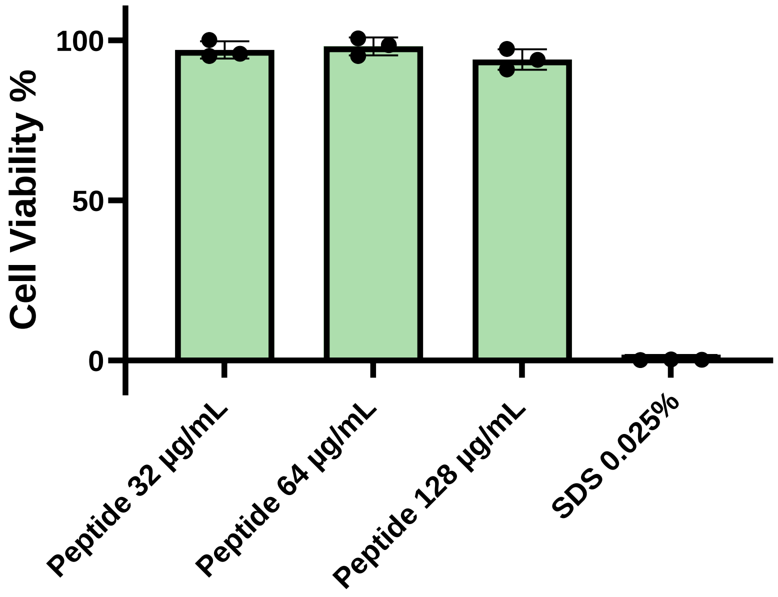

Figure S2 Cytotoxicity of peptide conjugate **P2-10** on mammalian HEK293FT cells at different concentrations. SDS (0.025%) was used as a positive control.

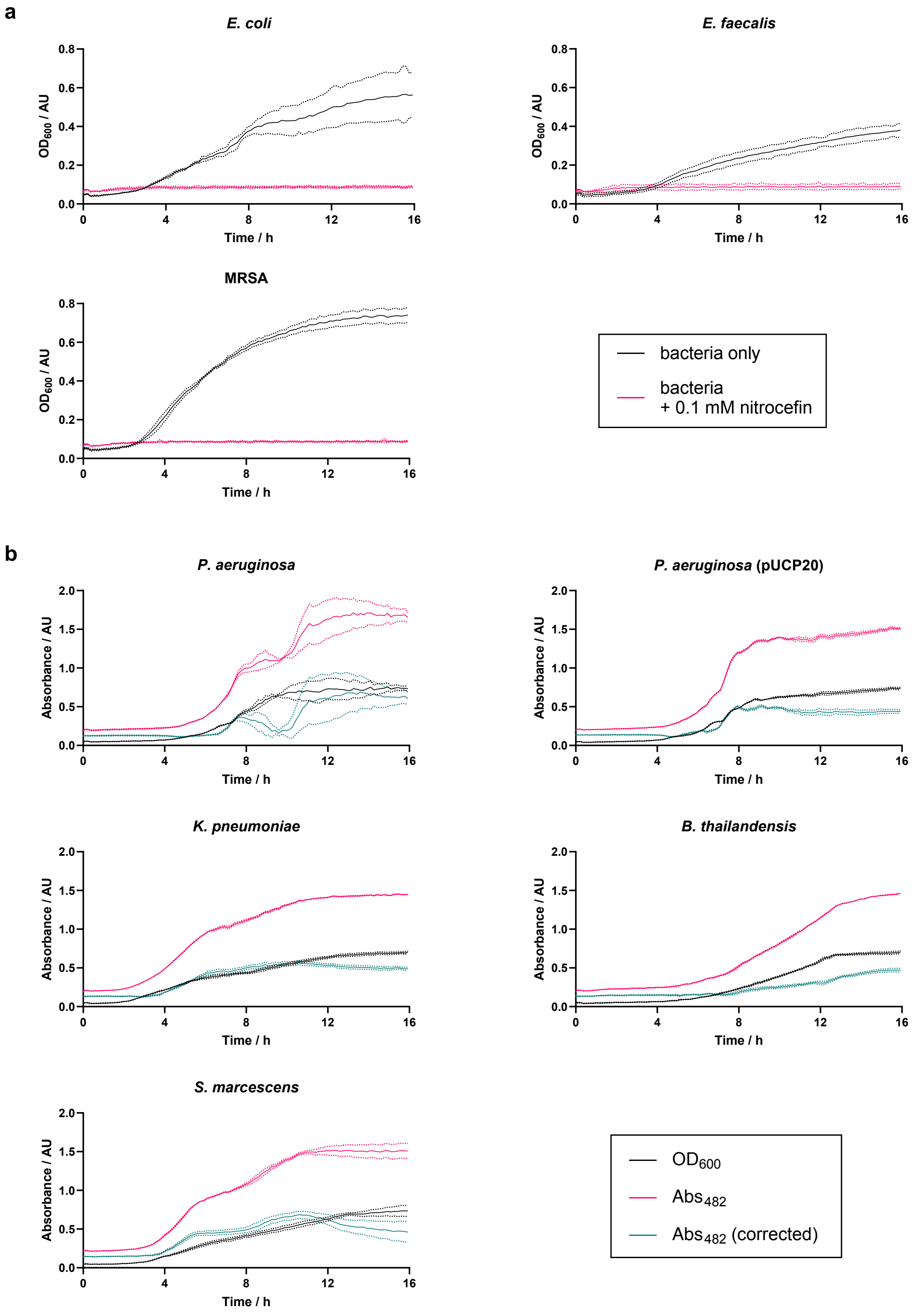

Figure S3 Curves following growth of bacteria (OD_{600}) and hydrolysis of 100 μM nitrocefin dye (Abs_{482}) co-incubated with the bacteria. **a** Three bacteria did not grow when co-incubated with nitrocefin. **b** Five bacteria grew in the presence of nitrocefin and nitrocefin hydrolysis, correlated to presence of β-lactamase, could be measured. To account for the light scattering due to bacteria, Abs_{482} when nitrocefin was present was corrected by subtracting Abs_{482} measured without nitrocefin. Data shown as an average of triplicates with the dotted line representing standard deviation.

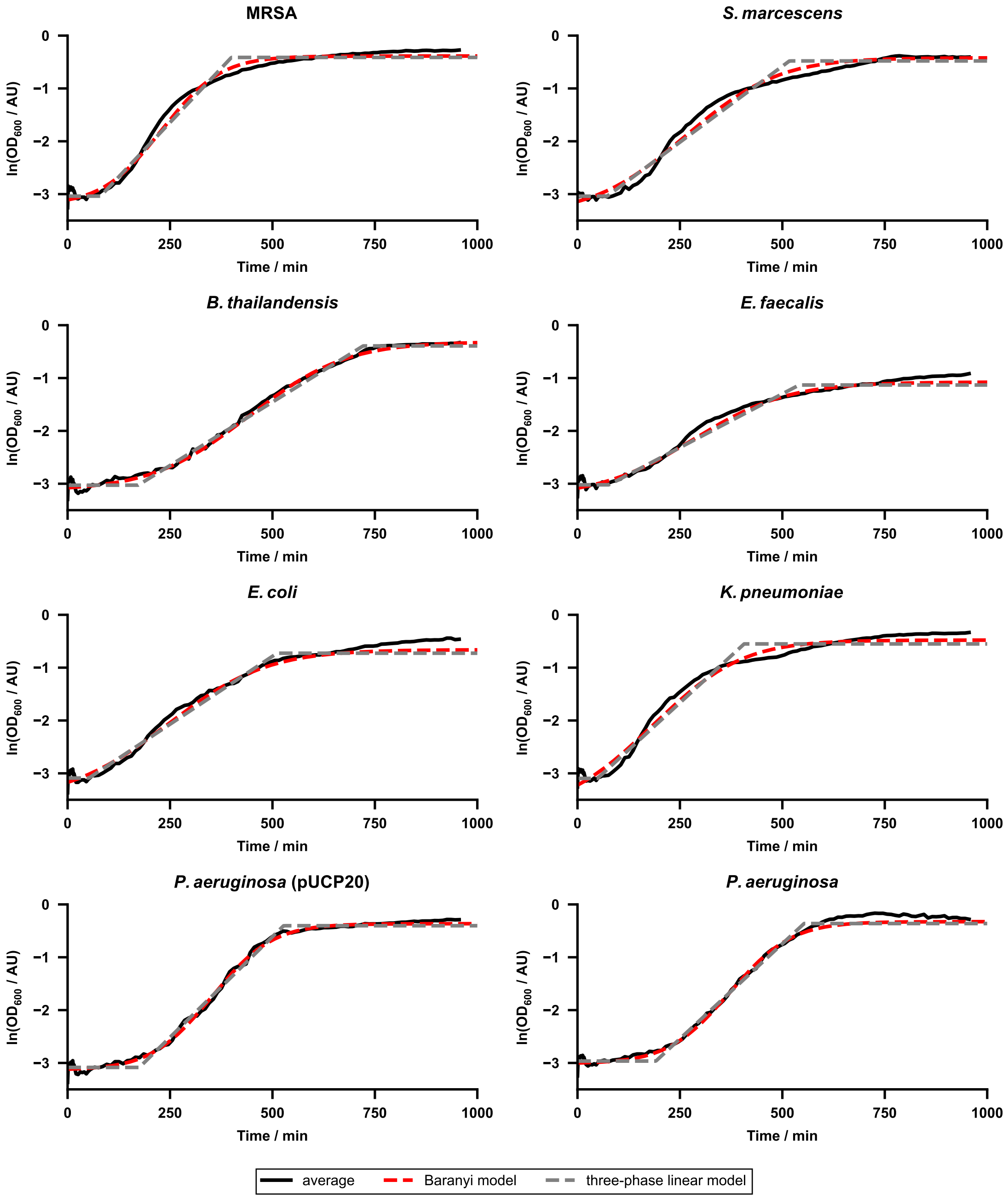

Strain	Baranyi model			Three-phase linear model		
	μ / min-1	λ / min	tD / min	μ / min-1	λ / min	tD / min
MRSA	0.01593	146.7	43.5	0.00822	80.5	84.4
S. marcescens	0.01035	145.3	67.0	0.00575	72.5	120.5
B. thailandensis	0.00974	303.3	71.2	0.00480	172.0	144.3
E. faecalis	0.00946	187.6	73.3	0.00408	76.7	170.0
E. coli	0.00956	132.0	72.5	0.00514	51.9	134.8
K. pneumoniae	0.01104	83.7	62.8	0.00711	48.8	97.5
P. aeruginosa (pUCP20)	0.01555	263.6	44.6	0.00761	175.1	91.0
P. aeruginosa	0.01508	282.8	46.0	0.00720	191.4	92.2

Figure S4 Growth curves from Figure S3 were fitted with curves following Baranyi and three-phase models.[2] The growth rate (μ), lag time (λ) and doubling time (t_D) parameters were obtained from the fitted curves and are summarised in the table.

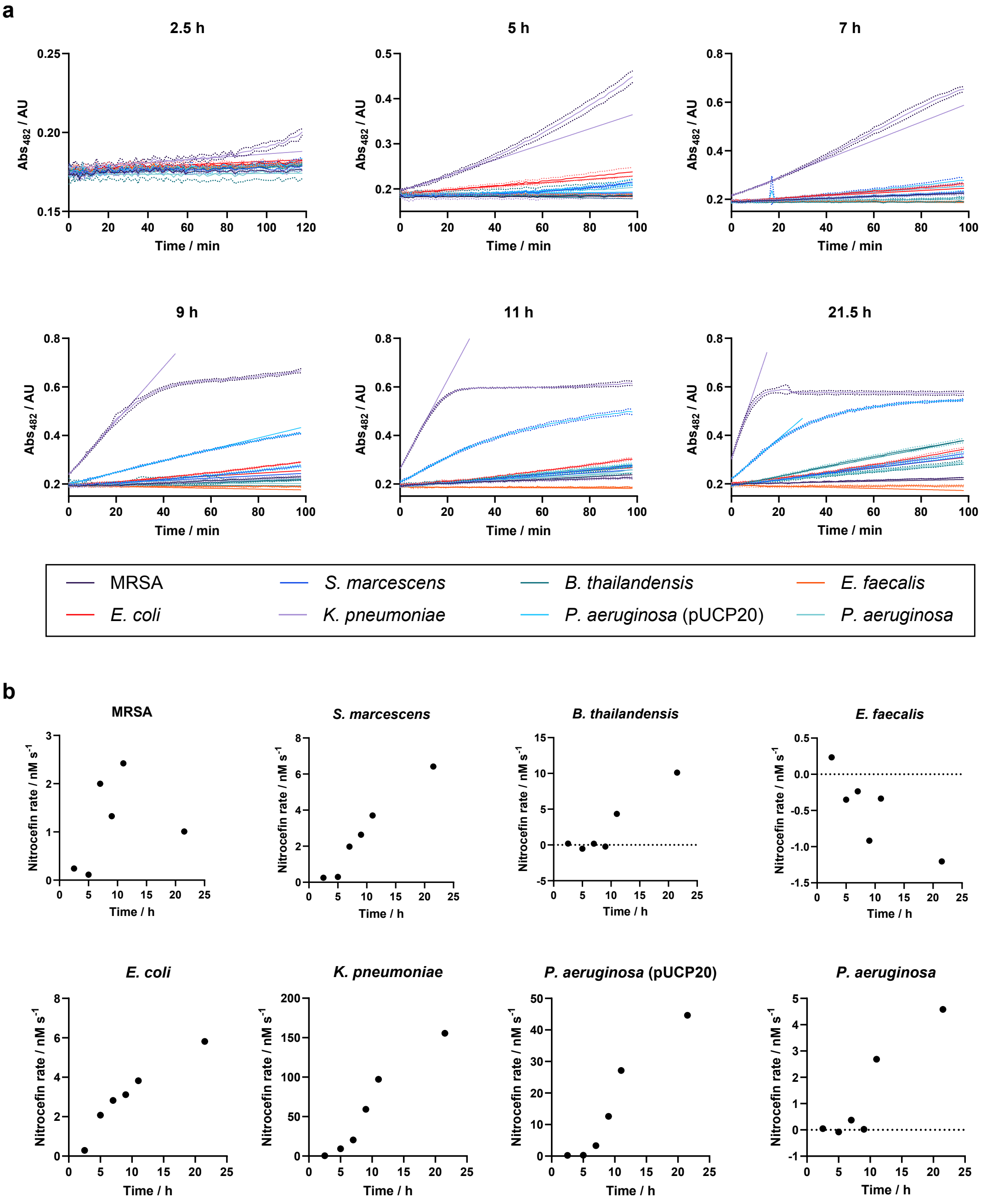

Figure S5 a Samples of bacterial culture were taken at six time points during the incubation and kinetics of the hydrolysis of nitrocefin by each of them measured. Data shown as an average of triplicates with the dotted line representing standard deviation. The initial rate of hydrolysis is highlighted by the straight lines of the corresponding colour. **b** The initial rate of nitrocefin hydrolysis for each bacterium at the different time points, corrected for the dilution factor of 2.

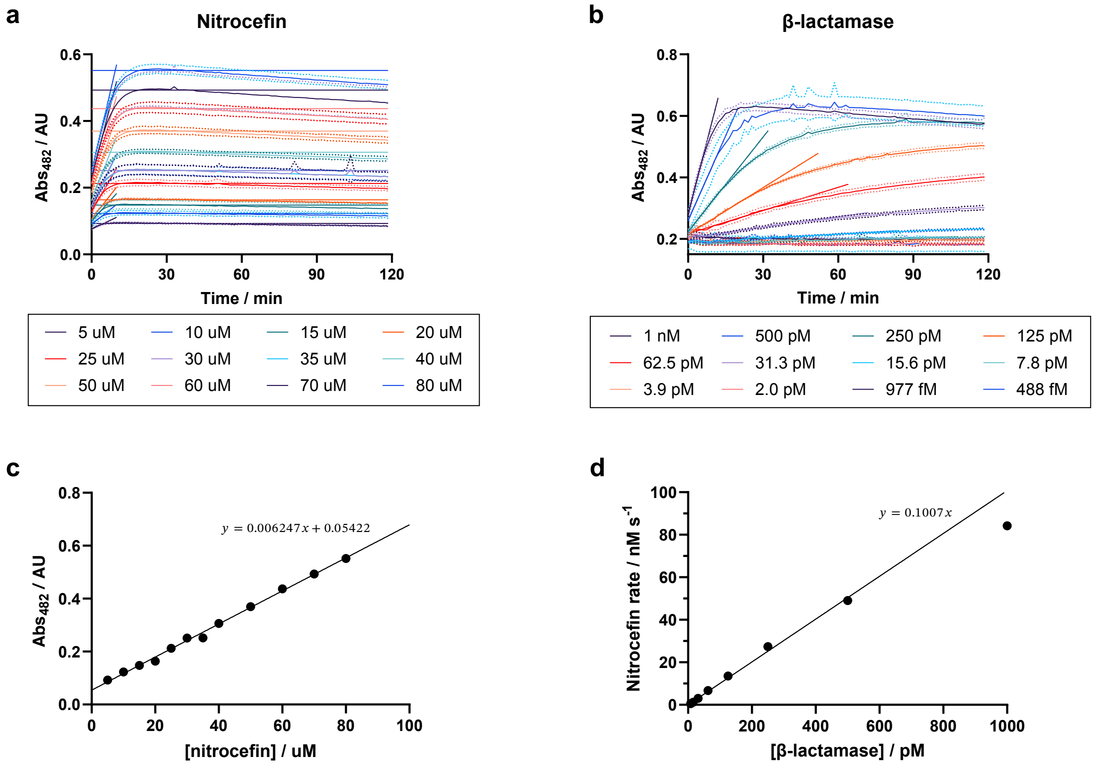

Figure S6 a Hydrolysis of different concentration of nitrocefin by 1 nM β-lactamase. Data shown as an average of triplicates with the dotted line representing standard deviation. The initial rate and the plateau are highlighted by the straight lines of the corresponding colour. **b** Hydrolysis of 100 µM of nitrocefin by varying amount of β-lactamase. Data shown as an average of triplicates with the dotted line representing standard deviation. The initial rate is highlighted by the straight lines of the corresponding colour. **c** Calibration curve correlating the plateau Abs$_{482}$ from (a) to the concentration of nitrocefin. **d** Calibration curve correlating the initial rate from (b) to the concentration of β-lactamase. The initial rate in AU s^{-1} was converted to nM s^{-1} using the slope of the trendline in (c). The last data point was not included due to low number of points defining the initial rate.

2 Supplementary tables

Table S1 Optimisation of the conditions for the deprotection of **P2-10a** by changing the ratios of TFA, H_2O, CH_2Cl_2 and a scavenger.

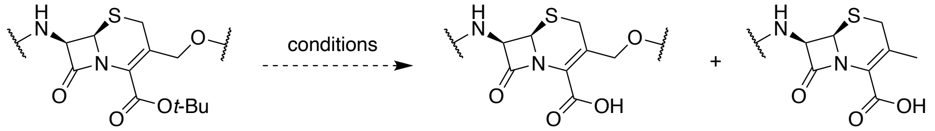

P2-10a **P2-10** **P2-10b**

Entry	Solvent mixture TFA / CH_2Cl_2 / TIPS / H_2O				Scavenger	LC–MS detected P2-10	LC–MS detected P2-10b
	TFA	CH_2Cl_2	TIPS	H_2O		**P2-10**	**P2-10b**
**1**	92.5	2.5	2.5	2.5	–	✓	✓
**2**	90	7.5	2.5	–	–	✓	✓
**3**	50	47.5	2.5	–	–	✓	✓
**4**	10	87.5	2.5	–	–	✗	✗
**5**	99	–	1	–	–	✓	✓
**6**	90	10	0.45	–	–	✓	✓
**7**	90	10	0.045	–	–	✓	✗
**8**	90	7.5	–	–	2.5 eq. of TES	✗	✓
**9**	92.5	2.5	–	2.5	2.5 eq. of $(TMS)_3SiH$	✗	✗

Table S2 Comparison of different protecting group strategies used for synthesis of the functionalised staple.

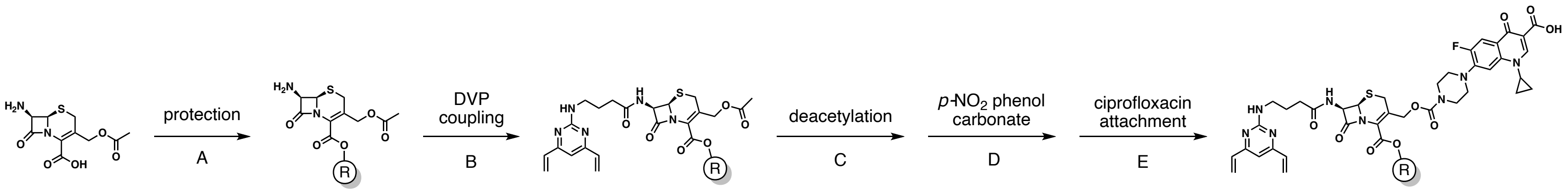

Protecting group R =		Step A Conditions	Step A Yield	Step B Yield	Step C LC–MS‡	Step D LC–MS‡	Step E Yield
t-Bu	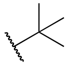	*t*-BuOAc<br>$BF_3 \cdot Et_2O$	67%	65%	✓	✓	18%
Bzh	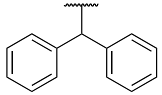	diphenyldiazomethane<br>CH_2Cl_2, MeOH	46%	62%*	✓	✓	✗
allyl	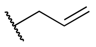	1) Boc_2O, Et_3N, H_2O, 1,4-dioxane<br>2) allyl alcohol, DCC, HOBt, CH_2Cl_2	32%	24%†	✓	✓	✗
methyl	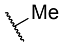	TMS-diazomethane<br>toluene, MeOH	46%	✗	–	–	–

* As a mixture of isomers.

† Over two steps including Boc deprotection.

‡ LC–MS detection of the desired product.

Table S3 Initial MIC values for stapled peptides where the *t*-butyl protecting group was not removed. Measured for *P. aeruginosa* strains PAO1 and PA14, and expressed in both mg/mL and µM.

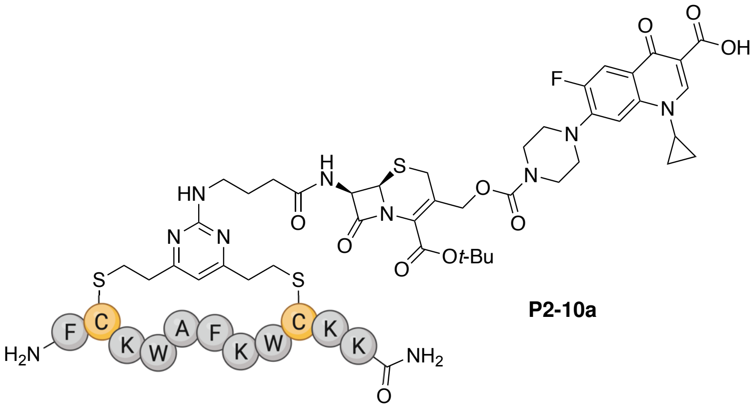

P2-10a

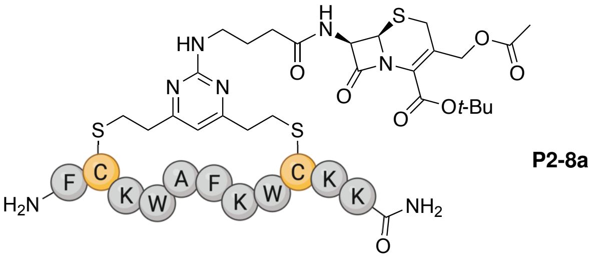

P2-8a

Compound	PAO1 MIC		PA14 MIC	
	µg/mL	µM	µg/mL	µM
**P2-10a**	64	27	32	14
**P2-8a**	64	32	64	32
**P2-10**	16	7.0	16	7.0
**P2-8**	>64	>33	>64	>33
Ciprofloxacin	0.125	0.38	0.0625	0.19
**P2a**	32	22	32	22
Polymyxin B	2	1.7	1	0.83

Table S4 MIC values for stapled peptides where the suitability of a more polar cephalosporin-based staple at different spots on a peptide was tested.

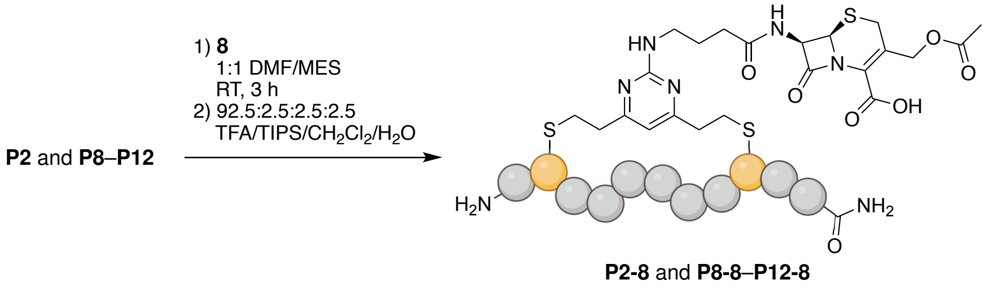

Compound	PAO1 MIC		PA14 MIC	
	µg/mL	µM	µg/mL	µM
**P2-8**	>64	>33	>64	>33
**P8-8**	>64	>35	>64	>35
**P9-8**	>64	>31	64	31
**P10-8**	>64	>35	>64	>35
**P11-8**	>64	>32	>64	>32
**P12-8**	>64	>33	>64	>33
**P2a**	16	11	32	22
Polymyxin B	1	0.83	1	0.83

Table S5 MIC values for stapled peptides where the linking region between the peptide and cephalosporin was altered. Peptide **P2** was stapled with staples **20a** and **21a** (Scheme S3) and deprotected using the standard procedures to give the peptide conjugates below.

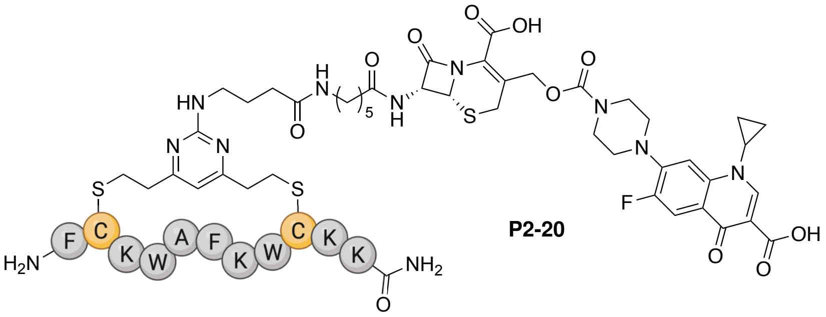

P2-20

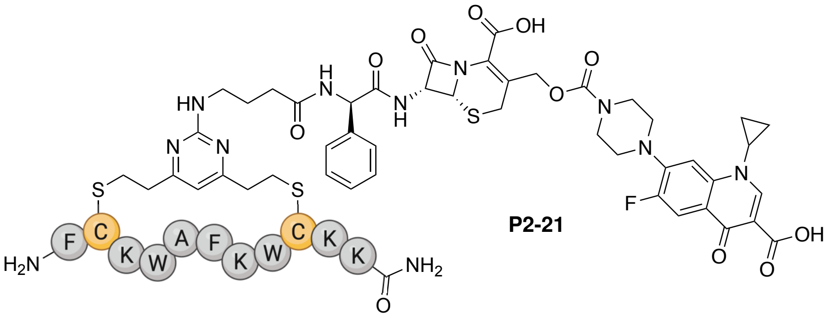

P2-21

Compound	PAO1 MIC		PA14 MIC	
	µg/mL	µM	µg/mL	µM
**P2-20**	32	13	32	13
**P2-21**	64	27	32	13
Ciprofloxacin	0.125	0.38	0.0625	0.19
**P2a**	16	11	32	22
Polymyxin B	1	0.83	1	0.83

3 General information

Solvents and chemicals

Reactions were carried out in oven- or flame-dried glassware under a nitrogen atmosphere. Tetrahydrofuran was always used freshly distilled from calcium hydride and $LiAlH_4$ in the presence of triphenyl methane. Dichloromethane, toluene, hexane and petroleum ether (refers to petroleum ether 40–60 °C) were distilled from calcium hydride. Diethyl ether, methanol, acetonitrile and all other reagents were used as provided by commercial sources (Sigma Aldrich, Fluorochem, Fischer Scientific).

Infrared spectroscopy

Infrared spectra were recorded on a Perkin-Elmer Spectrum One spectrometer with internal referencing fitted with an Attenuated Total Reflectance (ATR) sampling accessory, and Agilent Cary 630 FTIR with single bounce diamond ATR accessory using neat compounds or solutions (in which case solvent absorption was used for background). Absorption maxima ($\tilde{v}_{max}$) are reported in wavenumbers (cm^{-1}) rounded to a whole number. All peaks above 1500 cm^{-1} are reported. Only strong peaks below 1500 cm^{-1} that could be assigned are reported. The following abbreviations are used in assignment: aliph, aliphatic; arom, aromatic; conj, conjugated; tert, tertiary.

NMR spectroscopy

Magnetic resonance spectra were recorded at 298 K using an internal deuterium lock on Bruker Avance III HD (400 MHz; Smart probe), Bruker Avance III HD (500 MHz; Smart probe), Bruker Avance III HD (500 MHz; DCH Cryoprobe), 600 MHz Avance (600 MHz; Smart probe) BBI and 700 MHz TXO (700 MHz, Cryoprobe) spectrometers. Proton chemical shifts (δ_H) are quoted in ppm to the nearest 0.01 ppm and are referenced to the residual non-deuterated solvent peak ($CDCl_3$: 7.26, d_6-DMSO: 2.50, CD_3OD: 3.31). Carbon chemical shifts (δ_C) are quoted in ppm to the nearest 0.1 ppm and are referenced to the deuterated solvent ($CDCl_3$: 77.2, d_6-DMSO: 39.5, CD_3OD: 49.0). Fluorine chemical shifts (δ_F) are quoted in ppm to the nearest 0.1 ppm and are not referenced. Coupling constants (J) are reported in Hertz to the nearest 0.1 Hz. Data are reported as follows: chemical shift, integration, multiplicity [br, broad; app, apparent; s, singlet; d, doublet; t, triplet; q, quartet; quint, quintet; sext, sextet; sept, septet; m, multiplet; or as a combination of these (e.g. app s, dd, dt, etc.)], coupling constant(s) and assignment. Proton and carbon assignments are supported by DEPT135, ^{1}H-^{1}H COSY, ^{1}H-^{13}C HSQC and ^{1}H-^{13}C HMBC spectra. Some assignments were made by analogy to already reported and assigned molecules of similar structure. The numbering of the compounds does not follow IUPAC convention.

Column chromatography and TLC

Flash chromatography was done using Millipore Silica gel 60 (0.040–0.063 mm and distilled solvents. Thin layer chromatography was done using Supelco glass plates covered with TLC Silica gel 60 F_{254} and distilled solvents as eluents. The TLC plates were visualised by UV unless otherwise specified. If specified, the following stains were used: $KMnO_4$ (1.05 g of $KMnO_4$, 7 g of K_2CO_3, 90 mg of NaOH in 100 mL of water), Seebach's stain (2.5 g of phosphomolybdic acid, 1 g of cerium(IV) sulfate and 6 mL conc. H_2SO_4 in 94 mL of water), ninhydrin (1.5 g of ninhydrin in 100 mL of n-butanol with 3 mL of acetic acid). The retention factor R_f was quoted to two decimal places.

HPLC

Analytical HPLC was done on Agilent 1260 Infinity Series, fitted with a binary pump, using Supelco Supelcosil ABZ+PLUS (4.6 mm x 150 mm, particle size 3 µm, porosity 120 Å) column and operated by ChemStation C.01.03 software; or Agilent 1200 Series, fitted with a quaternary pump, using Agilent Eclipse Plus C18 (4.6 mm x 150 mm, particle size 3.5 µm, porosity 95 Å) column and operated by ChemStation B.04.03 software. The LC systems used a linear gradient of solvent B (acetonitrile with 0.05% TFA) in solvent A (solventwater with 0.05% TFA) run over 15 min, flow rate 1 mL/min, and UV absorption was measured using a diode-array detector and extracted wavelengths of 220 nm and 254 nm. Unless otherwise specified the gradient of 5% to 95% solvent B was used. The retention time t_R was quoted to 0.01 min.

Preparative HPLC was performed on Agilent 1260 Infinity Series, fitted with a binary pump cluster, using Supelco Supelcosil ABZ+PLUS (21.2 mm x 250 mm, particle size 5 µm, porosity 100 Å) or Agilent 10 Prep-C18 (21.2 mm x 250 mm, particle size 10 µm, porosity 100 Å) columns and operated by ChemStation C.01.07 software. The LC system used a linear gradient of solvent B (acetonitrile with 0.05% TFA) in solvent A (water with 0.1% TFA), flow rate 20 mL/min, and fractions were collected based on extracted wavelengths of 220 nm and 254 nm from an overall UV absorption measured using a diode-array detector. The run times and gradients were sample-specific.

LC–MS

Liquid chromatography–mass spectrometry (low-resolution mass spectrometry) was measured on Waters ACQUITY H-class UPLC with Waters SQ Detector 2 spectrometer fitted with an ESCi Multi-Mode probe and operated by MassLynx 4.1 software. The LC system used a gradient of 5–95% B with constant 5% C over 1 min at flow rate of 0.6 mL/min (solvent A: 2 mM NH_4OAc in H_2O/MeCN (95:5), solvent B: MeCN, solvent C: 2% formic acid); ACQUITY UPLC CSH C18 (2.1 mm x 50 mm, particle size 1.7 µm, porosity 130 Å) column was used; UV absorption was measured using a PDA eλdetector in 220–800 nm range, interval 1.2 nm; and molecules were ionised using electron spray ionisation (ESI).

HRMS

High-resolution mass spectrometry was measured on Waters Vion IMS QTof using ESI techniques. Mass values are reported within the error limits of ±5 ppm mass units.

Melting point and optical rotation

Melting points were measured on Gallenkamp melting point apparatus and are uncorrected. The optical rotation was measured on Anton Paar MCP 100 using a chamber with inner diametre of 5 mm and length of 100 mm. The concentrations are quoted in g/100 mL.

Automated flash chromatography

Normal phase automated flash chromatography was done on Biotage Isolera Four using Büchi FlashPure EcoFlex Silica cartridges or the same cartridges repacked with Millipore Silica gel 60 (0.040–0.063 mm and distilled solvents. The fractions were collected based on UV absorption at 254 nm and 280 nm.

Reversed-phase automated flash chromatography was done on CombiFlash R_f200 using RediSep R_f Gold C18 columns and HPLC-grade solvents. The fractions were collected based on UV absorption at 220 nm and 254 nm.

4 Experimental procedures

General procedure A: Nucleophilic substitution on heteroaromatic rings

Amine (1.2 eq) was suspended in a solution of heteroarene (1.0 eq) in acetone. The suspension was cooled to 0 °C and triethylamine (2.5 eq) was added slowly. The solution was stirred at 0 °C for 2 h and then for 1 h at RT. The reaction was diluted with ethyl acetate and the organic layer was washed with water (3×), dried over anhydrous $MgSO_4$ and the solvent was removed *in vacuo*. The crude product was purified using column chromatography.

General procedure B: Suzuki cross-coupling

Aryl chloride (1.0 eq), potassium vinyltrifluoroborate (3.0 eq) and potassium carbonate (6.0 eq) were dissolved in a 10:1 mixture of 1,4-dioxane and water. The solution was flushed with nitrogen for 15 min then [1,1′-bis(diphenylphosphino)ferrocene]dichloropalladium(II), dichloromethane complex (0.1–0.15 eq) added and the mixture stirred at 90 °C overnight. After that, the solution was allowed to cool to ambient temperature, filtered through Celite, washed and then further diluted with ethyl acetate. The mixture was washed with water, brine, dried over anhydrous Na_2SO_4, and the solvent was removed *in vacuo*. The crude product was purified using column chromatography.

N-(3-Phenylpropyl)-4,6-divinylpyrimidin-2-amine (1)

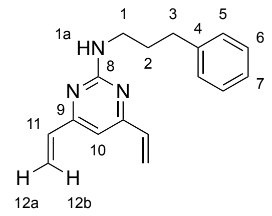

Following general procedure B, **16** (71 mg, 0.25 mmol), potassium vinyltrifluoroborate (100 mg, 0.75 mmol), potassium carbonate (207 mg, 1.50 mmol) and [1,1′-bis(diphenylphosphino)ferrocene]dichloropalladium(II), dichloromethane complex (31 mg, 0.038 mmol) were reacted in a mixture of 1,4-dioxane (3.0 mL) and water (0.3 mL). After purification using flash column chromatography (10% ethyl acetate in 40–60 petroleum ether), **1** (37 mg, 0.14 mmol, 56%) was obtained as a pale-yellow oil.

TLC (10% EA in PE): R_f 0.17

^{1}H-NMR (500 MHz, $CDCl_3$): δ 1.96 (2 H, quint, $J = 7.4$ Hz, H-2), 2.73 (2 H, t, $J = 7.7$ Hz, H-3), 3.52 (2 H, q, $J = 6.7$ Hz, H-1), 5.38 (1 H, br s, H-1a), 5.59 (2 H, d, $J = 10.6$ Hz, H-12a), 6.38 (2 H, d, $J = 17.3$ Hz, H-12b), 6.53 (1 H, s, H-10), 6.58 (2 H, dd, $J = 17.4, 10.6$ Hz, H-11), 7.16–7.23 (3 H, m, H-5/7), 7.28 (2 H, t, $J = 7.4$ Hz, H-6),

^{13}C-NMR (126 MHz, $CDCl_3$): δ 31.4 (C-2), 33.4 (C-3), 41.4 (C-1), 105.7 (C-10), 122.0 (C-12), 126.0 (C-7), 128.5 (C-5/6), 128.6 (C-5/6), 135.7 (C-11), 141.9 (C-4), 162.3 (C-8),[a] 163.7 (C-9)

FT-IR (neat) $\tilde{v}_{max}$: 3027 (CH, arom./alkene), 2937 (CH, aliph.), 1636 (C=C, alkene), 1541 (ring), 1325 (CN, arom.), 907 (CH, arom./alkene) cm^{-1}

HRMS-ESI (*m/z*): [M + H]$^+$ calcd for $[C_{17}H_{20}N_3]^+$, 266.1657; found, 266.1645

N-Methyl-*N*-(3-phenylpropyl)-4,6-divinylpyrimidin-2-amine (2)

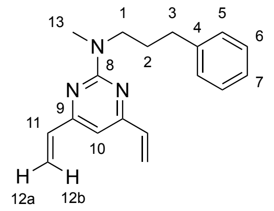

Following general procedure B, **17** (61 mg, 0.21 mmol), potassium vinyltrifluoroborate (84 mg, 0.63 mmol), potassium carbonate (174 mg, 1.26 mmol) and [1,1′-bis(diphenylphosphino)ferrocene]dichloropalladium(II), dichloromethane complex (26 mg, 0.032 mmol) were reacted in a mixture of 1,4-dioxane (2.5 mL) and water (0.25 mL). After purification using flash column chromatography (5% ethyl acetate in 40–60 petroleum ether), **2** (45 mg, 0.16 mmol, 77%) was obtained as a yellow oil.

TLC (5% EA in PE): R_f 0.38

^{1}H-NMR (500 MHz, $CDCl_3$): δ 1.98 (2 H, quint, $J = 7.5$ Hz, H-2), 2.67 (2 H, t, $J = 7.7$ Hz, H-3), 3.21 (3 H, s, H-13), 3.74 (2 H, t, $J = 7.3$ Hz, H-1), 5.53 (2 H, d, $J = 10.6$ Hz, H-12a), 6.35 (2 H, dd, $J = 17.3, 0.9$ Hz, H-12b), 6.44 (1 H, s, H-10), 6.60 (2 H, br dd, $J = 15.7, 10.6$ Hz, H-11), 7.16–7.24 (3 H, m, H-5/7), 7.28 (2 H, t, $J = 7.5$ Hz, H-6)[b]

^{13}C-NMR (126 MHz, $CDCl_3$): δ 28.9 (C-2), 33.4 (C-3), 35.3 (C-13), 49.0 (C-1), 104.4 (C-10), 121.0 (C-12), 125.9 (C-7), 128.4 (C-6), 128.5 (C-5), 136.4 (C-11), 142.3 (C-4), 162.1 (C-8),[c] 163.3 (C-9)

FT-IR (30 mg/mL solution in DMSO) $\tilde{v}_{max}$: 1651 (C=C, alkene), 1545 (ring), 662 (CH, arom.) cm^{-1}

HRMS-ESI (*m/z*): [M + H]$^+$ calcd for $[C_{18}H_{22}N_3]^+$, 280.1808; found, 280.1819

[a]The peak was confirmed by 2D NMR.
[b]The peak overlaps with the solvent peak.
[c]The peak was confirmed by 2D NMR.

N-(3-Phenylpropyl)-4,6-divinyl-1,3,5-triazin-2-amine (3)

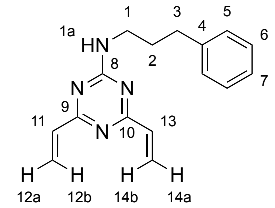

Following general procedure B, **18** (102 mg, 0.36 mmol), potassium vinyltrifluoroborate (145 mg, 1.08 mmol), potassium carbonate (300 mg, 2.16 mmol) and [1,1′-bis(diphenylphosphino)ferrocene]dichloropalladium(II), dichloromethane complex (44 mg, 0.054 mmol) were reacted in a mixture of 1,4-dioxane (4.5 mL) and water (0.45 mL). After purification using flash column chromatography (10% ethyl acetate in 40–60 petroleum ether), **3** (50 mg, 0.19 mmol, 52%) was obtained as a clear oil.

TLC (10% EA in PE): R_f 0.11

^{1}H-NMR (400 MHz, $CDCl_3$): δ 1.96 (2 H, quint, $J = 7.3$ Hz, H-2), 2.71 (2 H, t, $J = 7.6$ Hz, H-3), 3.53 (2 H, q, $J = 6.6$ Hz, H-1), 5.46 (1 H, br s, H-1a), 5.78 (2 H, d, $J = 10.1$ Hz, H-12a/14a), 6.49–6.78 (4 H, m, H-11/12b/13/14b), 7.16–7.23 (3 H, m, H-5/7), 7.29 (2 H, t, $J = 7.5$ Hz, H-6)

^{13}C-NMR (101 MHz, $CDCl_3$): δ 31.2 (C-2), 33.2 (C-3), 40.5 (C-1), 126.2 (C-7), 126.5 (C-12/14), 126.7 (C-12/14), 128.5 (C-5), 128.6 (C-6), 135.8 (C-11/13), 136.2 (C-11/13), 141.4 (C-4), 166.1 (C-8), 170.6 (C-9/10), 171.2 (C-9/10)

FT-IR (30 mg/mL solution in DMSO) $\tilde{\nu}_{max}$: 1579 (C=C, alkene), 1542 (ring), 849 (CH, arom.) cm^{-1}

HRMS-ESI (*m/z*): $[M + H]^+$ calcd for $[C_{16}H_{19}N_4]^+$, 267.1604; found, 267.1608

N-Methyl-*N*-(3-phenylpropyl)-4,6-divinyl-1,3,5-triazin-2-amine (4)

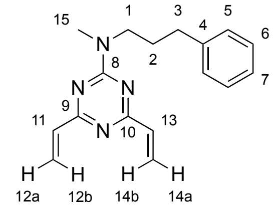

Following general procedure B, **19** (149 mg, 0.50 mmol), potassium vinyltrifluoroborate (201 mg, 1.50 mmol), potassium carbonate (415 mg, 3.00 mmol) and [1,1′-bis(diphenylphosphino)ferrocene]dichloropalladium(II), dichloromethane complex (61 mg, 0.075 mmol) were reacted in a mixture of 1,4-dioxane (6.0 mL) and water (0.6 mL). After purification using flash column chromatography (5% ethyl acetate in 40–60 petroleum ether), **4** (105 mg, 0.37 mmol, 75%) was obtained as a clear oil.

TLC (5% EA in PE): R_f 0.18

^{1}H-NMR (700 MHz, $CDCl_3$): δ 1.98 (2 H, quint, $J = 7.6$ Hz, H-2), 2.67 (2 H, t, $J = 7.7$ Hz, H-3), 3.20 (3 H, s, H-15), 3.72 (2 H, dd, $J = 8.1, 6.6$ Hz, H-1), 5.72 (2 H, d, $J = 10.0$ Hz, H-12a/14a), 5.75 (2 H, d, $J = 10.7$ Hz, H-12a/14a), 6.56–6.66 (3 H, m, H-11/12b/13/14b), 6.72 (1 H, d, $J = 17.1$ Hz, H-12b/14b), 7.14–7.22 (3 H, m, H-5/6/7), 7.26–7.30 (2 H, m, H-5/6/7),

^{13}C-NMR (176 MHz, $CDCl_3$): δ 28.7 (C-2), 33.2 (C-3), 34.6 (C-15), 48.4 (C-1), 125.7 (C-12/14), 126.1 (C-7), 128.48 (C-5/6), 128.52 (C-5/6), 136.6 (C-11/13), 141.7 (C-4), 165.2 (C-8), 170.3 (C-9/10), 170.6 (C-9/10)

FT-IR (30 mg/mL solution in DMSO) $\tilde{\nu}_{max}$: 1648 (C=C, alkene), 1562 (ring), 1552 (ring), 1509 (ring), 663 (CH, arom.) cm^{-1}

HRMS-ESI (*m/z*): $[M + H]^+$ calcd for $[C_{17}H_{21}N_4]^+$, 281.1761; found, 281.1769

***tert*-Butyl (6*R*,7*R*)-3-(acetoxymethyl)-7-amino-8-oxo-5-thia-1-azabicyclo[4.2.0]oct-2-ene-2-carboxylate (6)**

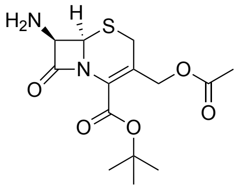

tert-Butyl acetate (4.1 mL) was purged with N_2 for 5 min and 7-aminocephalosporanic acid (250 mg, 0.92 mmol) was added. After that, boron trifluoride diethyl etherate (0.70 mL, 5.50 mmol) was added dropwise and the mixture was stirred at RT for 2 h. The mixture was then poured into an ice/water mixture and ethyl acetate added. The layers were separated, the aqueous layer was washed with a mixture of 40–60 petroleum ether and ethyl acetate (1:1) and the organic layer removed. Ethyl acetate was added to the aqueous layer and while the mixture was cooled to 0 °C and stirred vigorously, Na_2CO_3 was added slowly to adjust the pH to about 8.5. The organic layer was collected and the aqueous layer washed with ethyl acetate (3×). The combined organic layers were dried over anhydrous Na_2SO_4, the solvent was removed *in vacuo* and the residue triturated by stirring vigorously in a mixture of 40–60 petroleum ether and ethyl acetate (10:1) for 10 min to yield **6** (202 mg, 0.62 mmol, 67%) as a cream solid.

HPLC: t_R 7.44 min

^{1}H-NMR (700 MHz, d_6-DMSO): δ 1.46 (9 H, s), 2.02 (3 H, s), 3.44 (1 H, d, $J = 18.1$ Hz), 3.59 (1 H, d, $J = 18.1$ Hz), 4.61 (1 H, d, $J = 12.6$ Hz), 4.81 (1 H, d, $J = 5.1$ Hz), 4.89 (1 H, d, $J = 12.6$ Hz), 5.00 (1 H, d, $J = 5.1$ Hz)

^{13}**C-NMR** (176 MHz, d_6-DMSO): δ 20.6, 25.3, 27.4, 58.9, 62.7, 63.4, 82.5, 122.1, 127.0, 160.7, 169.4, 170.2

LRMS-ESI (*m/z*): [M + Na]$^+$ calcd for $[C_{14}H_{20}N_2NaO_5S]^+$, 351.1; found, 351.2

m.p. (PE): 93.0–97.0 °C

$[\alpha]_D^{20}$ −61.3 (*c* 0.16, $CHCl_3$)

The data are in accordance with previously reported values.[3,4]

4-((4,6-Divinylpyrimidin-2-yl)amino)butanoic acid (7)

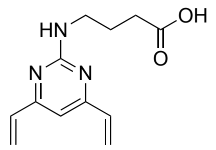

Compound **14** (967 mg, 3.70 mmol) was dissolved in a mixture of freshly distilled tetrahydrofuran (24.0 mL) and water (24.0 mL) and cooled to 0 °C. Lithium hydroxide monohydrate (466 mg, 11.1 mmol) was added at 0 °C, after which the mixture was stirred at RT overnight. The mixture was then diluted with water and 1 M HCl was added dropwise to adjust the pH to about 5. The mixture was extracted with dichloromethane (4×), the combined organics were dried over anhydrous $MgSO_4$ and the solvent was removed *in vacuo* to yield compound **7** (800 mg, 3.43 mmol, 93%) as a pale-yellow solid.

TLC (5% MeOH in CH_2Cl_2): R_f 0.24

HPLC: t_R 6.85 min

^{1}**H-NMR** (700 MHz, $CDCl_3$): δ 2.03 (2 H, quint, J = 6.8 Hz), 2.48 (2 H, t, J = 7.0 Hz), 3.60 (2 H, dd, J = 11.1, 5.9 Hz), 5.61 (2 H, dd, J = 10.7, 1.1 Hz), 6.20–6.42 (2 H, m), 6.51–6.64 (3 H, m), 6.97–7.06 (1 H, m), 11.16 (1 H, br s)

^{13}**C-NMR** (176 MHz, $CDCl_3$): δ 24.9, 32.3, 40.9, 103.8, 122.5, 135.2, 162.1, 163.9, 178.1

LRMS-ESI (*m/z*): [M + H]$^+$ calcd for $[C_{12}H_{16}N_3O_2]^+$, 234.1; found, 234.1

m.p. (CH_2Cl_2): 102.5–106.5 °C

The data are in accordance with previously reported values.[5]

***tert*-Butyl (6*R*,7*R*)-3-(acetoxymethyl)-7-(4-((4,6-divinylpyrimidin-2-yl)amino)butanamido)-8-oxo-5-thia-1-azabicyclo[4.2.0]oct-2-ene-2-carboxylate (8)**

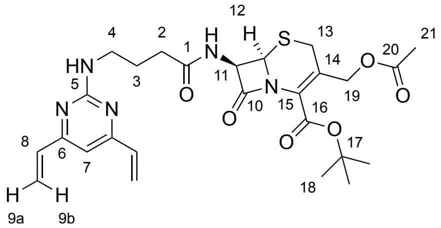

Compound **7** (204 mg, 0.88 mmol) was dissolved in dry *N*,*N*-dimethylformamide (8.0 mL) and 1-[bis(dimethylamino)methylene]-1*H*-1,2,3-triazolo[4,5-b]pyridinium 3-oxide hexafluorophosphate (402 mg, 1.06 mmol) and *N*,*N*-diisopropylethylamine (0.46 mL, 2.64 mmol) were added. After 15 min, **6** (288 mg, 0.88 mmol) was added and the mixture was stirred at RT for 3 h. The mixture was then diluted with ethyl acetate and washed with water, 5% solution of LiCl (4×) and brine. The organic layer was dried over anhydrous Na_2SO_4, the solvent was removed *in vacuo*, and the residue purified using automated reversed-phase flash column chromatography (gradient of 5–95% acetonitrile in 0.1 M NH_4OH). After freeze-drying, compound **8** (311 mg, 0.57 mmol, 65%) was obtained as a pale-yellow amorphous solid.

TLC (5% MeOH in CH_2Cl_2): R_f 0.35

HPLC: t_R 10.24 min

^{1}H-NMR (700 MHz, $CDCl_3$): δ 1.54 (9 H, s, H-18), 2.00 (2 H, quint, J = 6.8 Hz, H-3), 2.08 (3 H, s, H-21), 2.40 (2 H, t, J = 7.2 Hz, H-2), 3.35 (1 H, d, J = 18.3 Hz, H-13), 3.49–3.61 (3 H, m, H-4/13), 4.81 (1 H, d, J = 13.2 Hz, H-19), 4.97 (1 H, d, J = 4.8 Hz, H-12), 5.07 (1 H, d, J = 13.2 Hz, H-19), 5.64 (2 H, d, J = 10.1 Hz, H-9a), 5.85 (1 H, dd, J = 8.7, 4.8 Hz, H-11), 6.40 (2 H, d, J = 17.4 Hz, H-9b), 6.55–6.62 (3 H, m, H-7/8)[d]

^{13}C-NMR (176 MHz, $CDCl_3$): δ 20.9 (C-21), 25.7 (C-13), 26.6 (C-3), 27.9 (C-18, 33.4 (C-2), 40.6 (C-4), 57.5 (C-11), 59.5 (C-12), 63.3 (C-19), 84.0 (C-17), 105.4 (C-7),[e] 123.2 (C-9),[e] 123.4 (C-15), 127.7 (C-14), 135.3 (C-8),[e] 160.5 (C-10), 164.2 (C-5/6), 164.6 (C-1), 170.8 (C-16/20), 173.0 (C-16/20)

FT-IR (neat) $\tilde{\nu}_{max}$: 3287 (NH), 2979 (CH, arom.), 2940 (CH, aliph.), 1775 (C=O, β-lactam), 1740 (C=O, ester), 1720 (C=O, conj. ester), 1656 (C=O, amide), 1542 (C=C, arom.), 1221 (CO, ester), 1151 (CO, ester) cm^{-1}

HRMS-ESI (*m/z*): $[M + H]^+$ calcd for $[C_{26}H_{34}N_5O_6S]^+$, 544.2230; found, 544.2216

$[\alpha]_D^{20}$ +56.3 (*c* 0.16, $CHCl_3$)

[d]Amide protons not observed.
[e]Broad peak.

(6*R*,7*R*)-2-(*tert*-Butoxycarbonyl)-7-(4-((4,6-divinylpyrimidin-2-yl)amino)butanamido)-8-oxo-5-thia-1-azabicyclo[4.2.0]oct-2-en-3-yl)methoxy)carbonyl)piperazin-1-yl)-1-cyclopropyl-6-fluoro-4-oxo-1,4-dihydroquinoline-3-carboxylic acid (10)

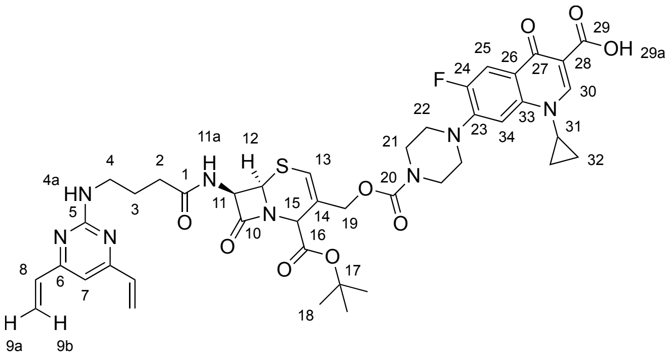

In dry glassware, compound **8** (116 mg, 0.214 mmol) was dissolved in tetrahydrofuran (1.14 mL). Hexane (10.3 mL) was added and a suspension formed. 2-Butanol (380 µL), CAL-B lipase on beads (95 mg; immobilised on Immobead 150, recombinant from *Aspergillus oryzae*, 2542 U/kg; Sigma-Aldrich, 54326) and molecular sieves (ca. 100 mg; 4 Å, 8–12 mesh) were added and the mixture heated with occasional gentle stirring at 50 °C for 3 d in an incubator. The mixture was diluted with dichloromethane, filtered and the filtrate evaporated *in vacuo*.

The residue was dissolved in *N*,*N*-dimethylformamide (1.0 mL), *N*,*N*-diisopropylethylamine (188 µL, 1.08 mmol) and bis(4-nitrophenyl) carbonate (131 mg, 0.43 mmol) were added and the mixture stirred at RT for 2 d. The solvent was evaporated under a stream of N_2, residue diluted with water and dichloromethane, and layers separated. The organic layer was washed with brine, 1 M KOH solution, brine, then dried over anhydrous $MgSO_4$ and the solvent was removed *in vacuo*. The residue was dissolved in 1,4-dioxane (1.35 mL) and added to a suspension of ciprofloxacin (75 mg, 0.23 mmol) and sodium bicarbonate (91 mg, 1.08 mmol) in water (1.35 mL) pre-mixed for 40 min at 45 °C. The mixture was stirred at RT overnight. Afterwards, water and dichloromethane were added, the aqueous layer was acidified with 1 M HCl to pH 4, layers separated and the aqueous layer further extracted with dichloromethane (3×). The combined organic layers were dried over anhydrous $MgSO_4$, the solvent was removed *in vacuo* and the residue purified using automated reversed-phase flash column chromatography (gradient of 5–95% acetonitrile in 0.1 M NH_4OH). After freeze-drying, compound **10** (33.5 mg, 0.039 mmol, 18% over two steps) was obtained as a cream amorphous solid.

HPLC: t_R 11.55 min

^{1}H-NMR (700 MHz, $CDCl_3$): δ 1.17–1.22 (2 H, m, H-32), 1.38–1.42 (2 H, m, H-32), 1.50 (9 H, s, H-18), 1.99 (2 H, quint, $J = 6.9$ Hz, H-3), 2.38 (2 H, t, $J = 7.1$ Hz, H-2), 3.28–3.33 (4 H, m, H-21/22), 3.52–3.57 (3 H, m, H-4/31), 3.69–3.77 (4 H, m, H-21/22), 4.72 (1 H,

d, J = 12.5 Hz, H-19), 4.75 (1 H, d, J = 12.7 Hz, H-19), 4.94 (1 H, s, H-15), 5.19–5.22 (1 H, br m, H-4a), 5.31 (1 H, d, J = 4.0 Hz, H-12), 5.56 (2 H, dd, J = 10.6, 0.9 Hz, H-9a), 5.67 (1 H, dd, J = 8.7, 4.0 Hz, H-11), 6.31–6.38 (3 H, m, H-9b/11a), 6.43 (1 H, s, H-13), 6.54 (1 H, s, H-7), 6.55–6.60 (2 H, m, H-8), 7.38 (1 H, d, J = 6.9 Hz, H-34), 8.06 (1 H, d, J = 12.6 Hz, H-25), 8.79 (1 H, s, H-30), 14.94 (1 H, br s, H-29a)

^{13}C-NMR (176 MHz, $CDCl_3$): δ 8.4 (C-32), 8.5 (C-32), 25.7 (C-3), 28.1 (C-18), 33.5 (C-2), 35.5 (C-31), 40.7 (C-4), 43.7 (C-21/22), 44.0 (C-21/22), 49.8 (C-21/22), 50.8 (C-15), 53.9 (C-12), 60.5 (C-11), 66.9 (C-19), 84.3 (C-17), 105.4 (d, J = 2.3 Hz, C-34), 105.8 (C-7), 108.5 (C-28), 112.9 (d, J = 23.4 Hz, C-25), 120.6 (C-14), 120.7 (d, J = 7.8 Hz, C-26), 121.6 (C-13), 121.7 (C-9), 136.0 (C-8), 139.2 (C-33), 145.7 (C-23), 147.8 (C-30), 153.8 (d, J = 251.3 Hz, C-24), 154.7 (C-20), 162.8 (C-5), 163.9 (C-6), 165.1 (C-10), 166.0 (C-16), 167.1 (C-29), 172.8 (C-1), 177.3 (C-27)

^{19}F-NMR (471 MHz, $CDCl_3$): δ −121.2

FT-IR (neat) $\tilde{v}_{max}$: 3305 (NH), 2937 (CH, aliph.), 1776 (C=O, β-lactam), 1703 (C=O), 1687 (C=O), 1627 (C=O, amide), 1544 (C=C), 1510 (ring), 1459 (ring), 1335 (CN, arom.), 1241 (CF, arom.), 1149 (CO, ester), 835 (CH, arom./alkene) cm^{-1}

LRMS-ESI (*m/z*): $[M + H]^+$ calcd for $[C_{42}H_{48}FN_8O_9S]^+$, 859.3; found, 859.3

$[\alpha]_D^{20}$ +157.5 (*c* 0.16, $CHCl_3$)

Ethyl 1-cyclopropyl-6-fluoro-4-oxo-7-(piperazin-1-yl)-1,4-dihydroquinoline-3-carboxylate (11)

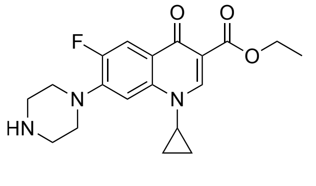

Into a suspension of ciprofloxacin (1.00 g, 3.02 mmol) in ethanol (25.0 mL) at 0 °C was added thionyl chloride (4.38 mL, 60.1 mmol) dropwise and the mixture was refluxed overnight. Afterwards, the solvent was removed *in vacuo* and the residue poured into cold saturated $NaHCO_3$ solution. The mixture was extracted with dichloromethane (3×), combined organic layers dried over anhydrous $MgSO_4$ and the solvent was removed *in vacuo* to yield compound **11** (686 mg, 1.91 mmol, 63%) as a cream solid.

TLC (5% MeOH in CH_2Cl_2): R_f 0.02

HPLC: t_R 6.24 min

^{1}H-NMR (400 MHz, $CDCl_3$): δ 1.10–1.17 (2 H, m), 1.29–1.35 (2 H, m), 1.40 (3 H, t, $J = 7.1$ Hz), 3.11–3.18 (4 H, m), 3.24–3.32 (4 H, m), 3.43 (1 H, sept, $J = 3.5$ Hz), 4.38 (2 H, q, $J = 7.1$ Hz), 7.26–7.29 (1 H, m), 8.04 (1 H, d, $J = 13.3$ Hz), 8.53 (1 H, s)

^{13}C-NMR (176 MHz, $CDCl_3$): δ 8.3, 14.6, 34.6, 46.1, 51.2 (d, $J = 4.4$ Hz), 61.0, 104.8 (d, $J = 3.0$ Hz), 110.6, 113.4 (d, $J = 23.1$ Hz), 123.2 (d, $J = 7.0$ Hz), 138.2, 145.1 (d, $J = 10.5$ Hz), 148.3, 153.6 (d, $J = 248.7$ Hz), 166.0, 173.3 (d, $J = 2.1$ Hz)

^{19}F-NMR (471 MHz, $CDCl_3$): δ −123.6

LRMS-ESI (*m/z*): $[M + H]^+$ calcd for $[C_{19}H_{23}FN_3O_3]^+$, 360.2; found, 360.3

m.p. (CH_2Cl_2): 215.0 °C dec

The data are in accordance with previously reported values.[6]

1-Cyclopropyl-7-(4-(4-((4,6-divinylpyrimidin-2-yl)amino)butanoyl)piperazin-1-yl)-6-fluoro-4-oxo-1,4-dihydroquinoline-3-carboxylic acid (12)

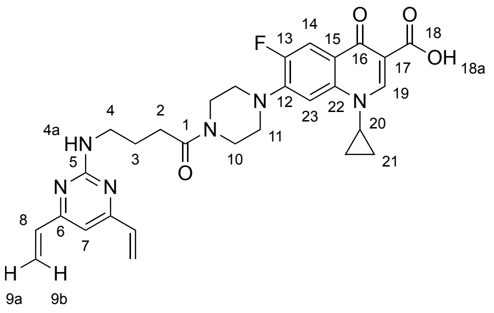

To a suspension of **11** (70 mg, 0.19 mmol) and **7** (50 mg, 0.21 mmol) in *N*,*N*-dimethylformamide (2.2 mL) with triethylamine (75 µL, 0.54 mmol) was added propylphosphonic anhydride (187 mL, 0.32 mmol, 50% w/w solution in *N*,*N*-dimethylformamide) and a solution formed. The mixture was stirred at RT over 3 d, after which it was diluted with ethyl acetate, washed with saturated $NaHCO_3$ solution, 10% citric acid solution, 5% LiCl solution and the organic layer dried over anhydrous $MgSO_4$. The solvent was removed *in vacuo* and the residue dried under high vacuum, after which it was dissolved in a mixture of freshly distilled tetrahydrofuran (1.1 mL) and water (1.1 mL). The mixture was cooled to 0 °C, lithium hydroxide monohydrate (22.5 mg, 0.54 mmol) was added and the mixture was then stirred at RT for 3 d. The mixture was then diluted with water and 1 M HCl was added dropwise to adjust pH to 5. The aqueous layer was extracted with dichloromethane (5×), the combined organics dried over anhydrous $MgSO_4$ and the solvent was removed *in vacuo* to yield compound **12** (49 mg, 0.14 mmol, 55%) as a yellow solid.

TLC (5% MeOH in CH_2Cl_2): R_f 0.01

HPLC: t_R 9.44 min

^{1}H-NMR (400 MHz, d_6-DMSO): δ 1.14–1.21 (2 H, m, H-21), 1.28–1.34 (2 H, m, H-21), 1.82 (2 H, quint, J = 6.6 Hz, H-3), 2.45 (2 H, t, J = 7.0 Hz, H-2), 3.26–3.37 (6 H, m, H-4/10),[f] 3.63–3.72 (4 H, m, H-11), 3.77–3.84 (1 H, m, H-20), 5.58 (2 H, d, J = 10.5 Hz, H-9a), 6.35 (2 H, d, J = 16.4 Hz, H-9b), 6.58 (2 H, dd, J = 17.2, 10.5 Hz, H-8), 6.77 (1 H, s, H-7), 7.10 (1 H, t, J = 5.2 Hz, H-4a), 7.56 (1 H, d, J = 7.5 Hz, H-23), 7.93 (1 H, d, J = 13.4 Hz, H-14), 8.67 (1 H, s, H-19), 15.19 (1 H, s, H-18a)

^{13}C-NMR (101 MHz, $CDCl_3$): δ 8.3 (C-21), 25.1 (C-3), 30.5 (C-2), 35.3 (C-20), 41.0 (C-4), 41.2 (C-10), 45.2 (C-10), 49.4 (d, J = 2.7 Hz, C-11), 50.2 (d, J = 6.3 Hz, C-11), 105.0 (d, J = 3.3 Hz, C-23), 105.6 (C-7), 108.4 (C-17), 112.7 (d, J = 23.7 Hz, C-14), 120.5 (C-9), 121.6 (C-15), 135.8 (C-8), 139.0 (d, J = 1.0 Hz, C-22), 145.4 (d, J = 10.1 Hz, C-12), 147.6 (C-19), 153.6 (d, J = 249.2 Hz, C-13), 162.6 (C-5), 163.7 (C-6), 166.9 (C-18), 171.3 (C-1), 177.2 (C-16)

^{19}F-NMR (376 MHz, $CDCl_3$): δ −121.2

FT-IR (neat) $\tilde{\nu}_{max}$: 3038 (CH, arom.), 2922 (CH, aliph.), 1732 (C=O), 1729 (C=O), 1625 (C=O, tert. amide), 1556 (C=C, arom.), 1212 (CF, arom.) cm^{-1}

HRMS-ESI (*m/z*): $[M + H]^+$ calcd for $[C_{29}H_{32}FN_6O_4]^+$, 547.2469; found, 547.2470

m.p. (CH_2Cl_2): 170.0 °C dec

Ethyl 4-((4,6-dichloropyrimidin-2-yl)amino)butanoate (13)

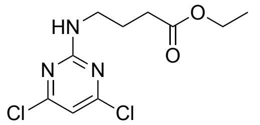

Following general procedure A, 2,4,6-trichloropyrimidine (3.0 g, 10.9 mmol) and ethyl 4-aminobutyrate hydrochloride (3.3 g, 13.1 mmol) were reacted in acetone (100 mL) with triethylamine (5.72 mL, 41.0 mmol). After purification using flash column chromatography (20% ethyl acetate in 40–60 petroleum ether), **13** (1.19 mg, 4.28 mmol, 39%) was obtained as a clear oil, which solidified on standing.

TLC (20% EA in PE): R_f 0.48

^{1}H-NMR (400 MHz, $CDCl_3$): δ 1.25 (3 H, t, J = 7.1 Hz), 1.93 (2 H, quint, J = 7.1 Hz), 2.38 (2 H, t, J = 7.2 Hz), 3.48 (2 H, q, J = 6.4 Hz), 4.14 (2 H, q, J = 7.1 Hz), 5.64 (1 H, br s), 6.59 (1 H, s)

^{13}C-NMR (101 MHz, $CDCl_3$): δ 14.3, 24.7, 31.7, 41.1, 60.7, 109.2, 173.2[g]

[f] The peak overlaps with the water peak.

[g] Two carbon peaks (corresponding to carbons attached to chlorine and three nitrogens) are missing.

LRMS-ESI (*m/z*): $[M + H]^+$ calcd for $[C_{10}H_{14}Cl_2N_3O_2]^+$, 278.0; found, 278.0

The data are in accordance with previously reported values.[5]

Ethyl 4-((4,6-divinylpyrimidin-2-yl)amino)butanoate (14)

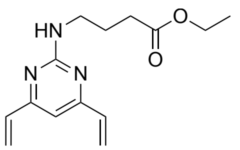

Following general procedure B, **13** (1.17 g, 4.20 mmol), potassium vinyltrifluoroborate (1.69 g, 12.6 mmol), potassium carbonate (3.49 g, 25.3 mmol) and [1,1′-bis(diphenylphosphino)ferrocene]dichloropalladium(II), dichloromethane complex (344 mg, 0.42 mmol) were reacted in a mixture of 1,4-dioxane (51.0 mL) and water (5.1 mL). After purification using flash column chromatography (25% ethyl acetate in 40–60 petroleum ether), **14** (988 mg, 3.78 mmol, 90%) was obtained as a yellow liquid.

TLC (25% EA in PE): R_f 0.25

^{1}H-NMR (700 MHz, $CDCl_3$): δ 1.24 (3 H, t, $J = 7.1$ Hz), 1.96 (2 H, quint, $J = 7.1$ Hz), 2.41 (2 H, t, $J = 7.4$ Hz), 3.53 (2 H, q, $J = 6.6$ Hz), 4.12 (2 H, q, $J = 7.1$ Hz), 5.20 (1 H, br s), 5.56 (2 H, d, $J = 10.7$ Hz), 6.36 (2 H, d, $J = 17.3$ Hz), 6.52 (1 H, s), 6.57 (2 H, dd, $J = 17.3, 10.6$ Hz),

^{13}C-NMR (176 MHz, $CDCl_3$): δ 14.4, 25.3, 31.9, 40.9, 60.5, 105.9, 121.6, 136.0, 162.6, 163.8, 173.6

LRMS-ESI (*m/z*): $[M + H]^+$ calcd for $[C_{14}H_{20}N_3O_2]^+$, 262.2; found, 262.2

The data are in accordance with previously reported values.[5]

***N*-Methyl-3-phenylpropan-1-amine (15)**

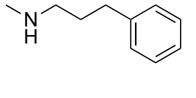

A solution of 1-bromo-3-phenylpropane (380 µL, 2.50 mmol) in ethanol (1.75 mL) was added dropwise at 0 °C to methylamine (4.4 mL, 33% w/w solution in ethanol). The mixture was stirred at RT overnight, after which the solvent was removed *in vacuo* and the residue purified using flash chromatography (7% methanol in dichloromethane with 1% triethylamine) to yield compound **15** (304 mg, 2.04 mmol, 81%) as a clear liquid.

TLC (5% MeOH in CH_2Cl_2): R_f 0.06

^{1}H-NMR (400 MHz, $CDCl_3$): δ 1.74 (1 H, br s), 1.83 (2 H, quint, J = 7.5 Hz), 2.44 (3 H, s), 2.62 (2 H, t, J = 7.3 Hz), 2.66 (2 H, t, J = 7.9 Hz), 7.15–7.22 (3 H, m), 7.25–7.31 (2 H, m)[h]

^{13}C-NMR (101 MHz, $CDCl_3$): δ 31.5, 33.8, 36.5, 51.7, 125.9, 128.48, 128.50, 142.2

LRMS-ESI (*m/z*): $[M + H]^+$ calcd for $[C_{10}H_{16}N]^+$, 150.1; found, 149.8

The data are in accordance with previously reported values.[7]

4,6-Dichloro-*N*-(3-phenylpropyl)pyrimidin-2-amine (16)

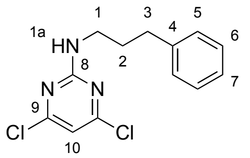

Following general procedure A, 2,4,6-trichloropyrimidine (167 mg, 0.91 mmol) and 3-phenyl-1-propylamine (155 µL, 1.09 mmol) were reacted in acetone (4.0 mL) with triethylamine (0.32 mL, 2.28 mmol). After purification using flash column chromatography (5% ethyl acetate in 40–60 petroleum ether), **16** (79 mg, 0.28 mmol, 31%) was obtained as a white solid.

TLC (10% EA in PE): R_f 0.31

^{1}H-NMR (400 MHz, $CDCl_3$): δ 1.94 (2 H, quint, J = 7.4 Hz, H-2), 2.70 (2 H, t, J = 7.7 Hz, H-3), 3.41–3.51 (2 H, m, H-1), 5.51 (1 H, br s, H-1a), 6.60 (1 H, s, H-10), 7.16–7.23 (3 H, m, H-5/7), 7.29 (2 H, t, J = 7.5 Hz, H-6)

^{13}C-NMR (101 MHz, $CDCl_3$): δ 30.8 (C-2), 33.0 (C-3), 41.3 (C-1), 109.1 (C-10), 126.2 (C-7), 128.5 (C-5), 128.6 (C-6), 141.3 (C-4), 161.7 (C-8/9)[i]

FT-IR (neat) $\tilde{\nu}_{max}$: 3271 (NH), 3122 (CH, arom.), 2934 (CH, aliph.), 1600 (C=C, arom.), 1518 (NH), 1450 (CH, aliph.), 696 (CH, arom.) cm^{-1}

HRMS-ESI (*m/z*): $[M + H]^+$ calcd for $[C_{13}H_{14}Cl_2N_3]^+$, 282.0565; found, 282.0557

m.p. (EA): 85.0–87.0 °C

[h] The peak overlaps with the solvent peak.
[i] The peak was identified with help from 2D NMR.

4,6-Dichloro-*N*-methyl-*N*-(3-phenylpropyl)pyrimidin-2-amine (17)

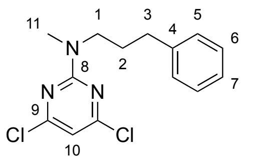

Following general procedure A, 2,4,6-trichloropyrimidine (186 mg, 1.01 mmol) and **15** (162 mg, 1.09 mmol) were reacted in acetone (4.0 mL) with triethylamine (0.32 mL, 2.10 mmol). After purification using flash column chromatography (5% ethyl acetate in 40–60 petroleum ether), **17** (70 mg, 0.24 mmol, 23%) was obtained as a clear oil.

TLC (5% EA in PE): R_f 0.20

^{1}H-NMR (400 MHz, $CDCl_3$): δ 1.95 (2 H, quint, $J = 7.3$ Hz, H-2), 2.65 (2 H, t, $J = 7.5$ Hz, H-3), 3.12 (3 H, s, H-11), 3.64 (2 H, t, $J = 7.2$ Hz, H-1), 6.50 (1 H, s, H-10), 7.15–7.23 (3 H, m, H-5/7), 7.28 (2 H, t, $J = 7.2$ Hz, H-6)

^{13}C-NMR (101 MHz, $CDCl_3$): δ 28.6 (C-2), 33.1 (C-3), 35.6 (C-11), 49.3 (C-1), 107.4 (C-10), 126.1 (C-5/7), 128.4 (C-5/7), 128.5 (C-6), 141.6 (C-4), 161.2 (C-8), 161.5 (C-9)

FT-IR (neat) $\tilde{\nu}_{max}$: 2932 (CH, arom.), 2863 (CH, aliph.), 1552 (ring), 1503 (ring), 1402 (ring), 1091 (CCl, arom.), 816 (CH, arom.), 697 (CH, arom.) cm^{-1}

HRMS-ESI (*m/z*): $[M + H]^+$ calcd for $[C_{14}H_{16}Cl_2N_3]^+$, 296.0716; found, 296.0723

4,6-Dichloro-*N*-(3-phenylpropyl)-1,3,5-triazin-2-amine (18)

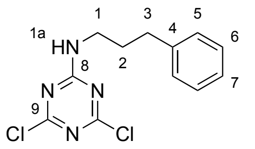

Following general procedure A, cyanuric chloride (155 mg, 0.84 mmol) and 3-phenyl-1-propylamine (144 µL, 1.01 mmol) were reacted in acetone (4.0 mL) with triethylamine (0.3 mL, 2.10 mmol). After purification using flash column chromatography (10% ethyl acetate in 40–60 petroleum ether), **18** (105 mg, 0.37 mmol, 44%) was obtained as a white solid.

TLC (10% EA in PE): R_f 0.18

^{1}H-NMR (400 MHz, $CDCl_3$): δ 1.96 (2 H, quint, $J = 7.3$ Hz, H-2), 2.70 (2 H, t, $J = 7.6$ Hz, H-3), 3.51 (2 H, q, $J = 6.7$ Hz, H-1), 5.86 (1 H, br s, H-1a), 7.15–7.25 (3 H, m, H-5/7), 7.30 (2 H, t, $J = 7.3$ Hz, H-6)

^{13}C-NMR (101 MHz, $CDCl_3$): δ 30.6 (C-2), 33.0 (C-3), 41.2 (C-1), 126.4 (C-7), 128.5 (C-5), 128.8 (C-6), 140.7 (C-4), 166.1 (C-8)[j]

FT-IR (neat) $\tilde{\nu}_{max}$: 3261 (NH), 3114 (CH, arom.), 2948 (CH, aliph.), 2859 (CH, aliph.), 1623 (ring), 1546 (ring), 1514 (ring), 1400 (ring), 1323 (CN, arom.), 1236 (CN, aliph.), 1156 (CCl, arom.), 795 (CH, arom), 696 (CH, arom.) cm^{-1}

HRMS-ESI (*m/z*): $[M + H]^+$ calcd for $[C_{12}H_{13}Cl_2N_4]^+$, 283.0512; found, 283.0522

m.p. (EA): 77.0–81.0 °C

4,6-Dichloro-*N*-methyl-*N*-(3-phenylpropyl)-1,3,5-triazin-2-amine (19)

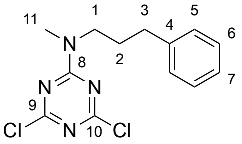

Following general procedure A, cyanuric chloride (155 mg, 0.84 mmol) and **15** (150 mg, 1.01 mmol) were reacted in acetone (4.0 mL) with triethylamine (0.3 mL, 2.10 mmol). After purification using flash column chromatography (10% ethyl acetate in 40–60 petroleum ether), **19** (160 mg, 0.54 mmol, 64%) was obtained as a clear oil.

TLC (10% EA in PE): R_f 0.26

^{1}H-NMR (700 MHz, $CDCl_3$): δ 1.97 (2 H, quint, $J = 7.6$ Hz, H-2), 2.66 (2 H, t, $J = 7.7$ Hz, H-3), 3.15 (3 H, s, H-11), 3.67 (2 H, dd, $J = 7.9, 6.7$ Hz, H-1), 7.12–7.23 (3 H, m, H-5/6/7), 7.24–7.31 (2 H, m, H-5/6/7)[k]

^{13}C-NMR (176 MHz, $CDCl_3$): δ 28.3 (C-2), 32.9 (C-3), 35.4 (C-11), 49.3 (C-1), 126.3 (C-7), 128.4 (C-5/6), 128.6 (C-5/6), 140.9 (C-4), 164.8 (C-8), 170.0 (C-9/10), 170.1 (C-9/10)

FT-IR (neat) $\tilde{\nu}_{max}$: 2935 (CH, aliph.), 2865 (CH, aliph.), 1560 (ring), 1476 (ring), 1402 (ring), 1327 (CN, arom.), 1232 (CN, aliph.), 1165 (CCl, arom.), 795 (CH, arom.), 698 (CH, arom.) cm^{-1}

HRMS-ESI (*m/z*): $[M + H]^+$ calcd for $[C_{13}H_{15}Cl_2N_4]^+$, 297.0668; found, 297.0674

[j] The peak was identified with help from 2D NMR. Peaks corresponding to carbons C-9 were not observed.

[k] The peak overlaps with the solvent peak.

5 Peptide synthesis and analysis

Initial stapling position computation

Peptides were modelled using Chimera. For peptides **P1–P7**, the staple was placed so that the overall cationic charge and amphipathicity, both of which are linked to antimicrobial activity, were preserved.[8] Additionally, any potentially unstable residues (Met) were replaced by the staple where possible.

Synthesis

Peptides were synthesised on a CEM Liberty Automated Microwave Peptide Synthesiser using Rink Amide MBHA low-loading resin (100–200 mesh, loading 0.3–0.4 mmol/g, Merck and Fluorochem) to afford C-terminal amides upon cleavage. Standard Fmoc-protected amino acids were used with the following side chain protecting groups: 2,2,4,6,7-pentamethyldihydrobenzofuran-5-sulfonyl (Arg), Boc (Lys, Trp, His), Trt (Cys, Asn, Gln), *t*-Bu (Thr, Tyr).

The resin was swelled in DMF before loading onto the machine. All peptide couplings were performed with Fmoc-protected amino acids (5 equiv.), Oxyma pure (10 equiv.) and DIC (5 equiv.) in DMF. Unless otherwise specified, amino acids were coupled using double coupling with 25 W power at 75 °C over 15 min. Arginine couplings were carried out twice for 15 min each without microwave irradiation. Cysteine couplings were carried out at 50 °C. Fmoc deprotection was achieved with a solution of 20% piperidine in DMF, using 45 W power at 75 °C over 3 min. If required, the N-terminus of the peptide was capped by acetylation using 20% acetic anhydride or octanoyl chloride solution in DMF solution at room temperature for 20 min.

Afterwards, the resin was drained of DMF, washed with CH_2Cl_2 and suspended in a cleavage mix of TFA/TIPS/CH_2Cl_2/water (92.5:2.5:2.5:2.5, v/v). The mixture was gently stirred at 40 °C for 2 h, resin filtered off and washed with methanol and the filtrate was concentrated under a stream of N_2. The residue was dissolved in minimal amount of trifluoroacetic acid and the peptide crashed out as a TFA salt in cold diethyl ether. The suspension was centrifuged, supernatant decanted, solid dissolved in DMSO and purified using preparative HPLC.

Peptide data

Table S6 Peptide sequences, scale and preparative HPLC parameters. Gradient is expressed in % of solvent B and its length in minutes is shown.

Peptide	Sequence	Scale	Purification	
			Gradient	Length
**P1**	Ac—TLKQFCKGVGKWCVK—NH_2	0.25 mmol	15–50	20 min
**P2**	H—FCKWAFKWCKK—NH_2	0.25 mmol	5–95	20 min
**P2a**	H—FAKWAFKWLKK—NH_2	0.1 mmol	10–50	17 min
**P3**	Ac—WCLKKFRGCF—NH_2	0.25 mmol	30–50	20 min
**P4**	H—RFRRLCKKWRKRCKKI—NH_2	0.25 mmol	15–40	20 min
**P4a**	H—RFRRLRKKWRKRLKKI—NH_2	0.1 mmol	30–70	20 min
**P5**	C_8—GCLKFIKKCL—NH_2	0.25 mmol	40–60	20 min
**P6**	H—KWVQNYCKHLGRKCHTLKT—NH_2	0.25 mmol	10–50	20 min
**P6a**	H—KWVQNYMKHLGRKAHTLKT—NH_2	0.1 mmol	5–95	20 min
**P7**	H—NLFRKLCHRLFRRCFGYTLR—NH_2	0.25 mmol	20–60	20 min
**P8**	H—CAKWAFKCLKK—NH_2	0.25 mmol	5–55	13 min
**P9**	H—FAKWCFKWLKKC—NH_2	0.25 mmol	15–60	13 min
**P10**	H—FAKCAFKWLKC—NH_2	0.1 mmol	15–60	13 min
**P11**	H—CFAKWAFCWLKK—NH_2	0.1 mmol	15–60	13 min
**P12**	H—KFAKCAFKWLKC—NH_2	0.1 mmol	15–60	13 min

Table S7 Peptide analytical data.

Peptide	Yield		LC-MS			HPLC	
	Mass	%*	Ion	Calculated	Observed	t_R	Purity
**P1**	57 mg	13	$[M + 2H]^{2+}$	884.1 Da	884.4 Da	6.93 min	84%
**P2**	129.5 mg	25	$[M + 2H]^{2+}$	737.4 Da	737.6 Da	6.06 min	92%
**P2a**	72.4 mg	36	$[M + 2H]^{2+}$	726.4 Da	726.6 Da	6.94 min	97%
**P3**	51 mg	15	$[M + 2H]^{2+}$	665.3 Da	665.4 Da	7.93 min	85%
**P4**	30 mg	5	$[M + 3H]^{3+}$	735.9 Da	735.9 Da	6.20 min	69%

Continued on next page

Table S7 Peptide analytical data. (Continued)

Peptide	Yield		LC-MS			HPLC	
	Mass	%*	Ion	Calculated	Observed	t_R	Purity
**P4a**	37 mg	16	$[M + 3H]^{3+}$	757.3 Da	757.1 Da	4.82 min	90%
**P5**	29 mg	9	$[M + H]^{+}$	1278.7 Da	1278.8 Da	10.23 min	95%
**P6**	14 mg	2	$[M + 2H]^{2+}$	1171.4 Da	1171.6 Da	6.00 min	99%
**P6a**	26 mg	11	$[M + 2H]^{2+}$	1170.5 Da	1170.9 Da	6.40 min	96%
**P7**	35 mg	5	$[M + 3H]^{3+}$	867.4 Da	867.2 Da	7.96 min	90%
**P8**	134 mg	28	$[M + 2H]^{2+}$	662.9 Da	663.1 Da	5.41 min	92%
**P9**	123 mg	23	$[M + 2H]^{2+}$	793.9 Da	794.1 Da	6.67 min	98%
**P10**	34 mg	19	$[M + 2H]^{2+}$	672.4 Da	672.7 Da	5.82 min	96%
**P11**	58 mg	29	$[M + 2H]^{2+}$	765.4 Da	764.9 Da	7.08 min	92%
**P12**	62 mg	30	$[M + 2H]^{2+}$	736.4 Da	736.9 Da	6.27 min	97%

* Calculated using a TFA salt of the peptide (added a molecule of TFA for N-terminus and each basic residue)

Peptide stapling and deprotection

All peptide stapling reactions were performed in 1:1 mixture of DMF and MES[1] buffer (50 mM, pH 6) and at final peptide concentration of 2 mg/mL. After mixing of all the starting materials the mixture was stirred for a given time, after which the solvent was removed under a stream of N_2 and the crude material purified using preparative HPLC.

If peptides were deprotected, they were first stapled using the conditions above, the solvent removed under a stream of nitrogen, the crude material dissolved in a deprotection mix of TFA/CH_2Cl_2/TIPS/water (92.5:2.5:2.5:2.5, v/v) and stirred for a given time. The solvent was then removed under a stream of N_2 and the crude material purified using preparative HPLC.

The labelling of the stapled peptides uses notation **PX-Y**, where **PX** is the number of the peptide that was used for stapling and **Y** is the number of the staple used for the stapling.

[1] 2-(*N*-morpholino)ethanesulfonic acid

Table S8 Peptide stapling, deprotection and purification conditions.

Stapled peptide	Peptide	Staple		Timing		Purification	
		Amount	Form	Stap.	Deprot.	Grad.	Length
**P1-1**	5.6 mg	23.1 µL (1.1 eq)	40 mg/mLin DMF	18 h	–	20–70	20 min
**P3-1**	5.0 mg	27.5 µL (1.1 eq)	30 mg/mLin DMSO	18 h	–	5–95	20 min
**P4-1**	5.6 mg	18.6 µL (1.1 eq)	40 mg/mLin DMF	18 h	–	20–70	20 min
**P5-1**	5.5 mg	31.3 µL (1.1 eq)	40 mg/mLin DMF	18 h	–	20–70	20 min
**P6-1**	5.0 mg	15 µL (1.1 eq)	30 mg/mLin DMSO	18 h	–	5–95	20 min
**P7-1**	7.0 mg	19.6 µL (1.1 eq)	40 mg/mLin DMF	18 h	–	5–95	20 min
**P2-10a**	4.0 mg	387 µL (1.4 eq)	6 mg/mLin DMF	2 h	–	20–60	20 min
**P2-10**	4.2 mg	2.45 mg (1.4 eq)	solid	2 h	30 min	15–40	20 min
**P2-8a**	4.1 mg	247 µL (1.4 eq)	6.19 mg/mLin DMF	2 h	–	20–60	20 min
**P2-8**	4 mg	1.06 mg (1.0 eq)	solid	3 h	50 min	15–40	20 min
**P2-12**	4.2 mg	1.53 mg (1.4 eq)	solid	2 h*	–	15–40	20 min
**P2-1**	4.0 mg	29 µL (1.7 eq)	30 mg/mLin DMF	2 h	–	5–80	14 min
**P2-2**	4.2 mg	32 µL (1.7 eq)	30 mg/mLin DMF	2 h	–	5–80	14 min
**P2-3**	4.1 mg	30 µL (1.7 eq)	30 mg/mLin DMF	2 h	–	5–95	14 min
**P2-4**	4.2 mg	32 µL (1.7 eq)	30 mg/mLin DMF	2 h	–	5–80	14 min

Continued on next page

Table S8 Peptide stapling, deprotection and purification conditions. (Continued)

Stapled peptide	Peptide	Staple		Timing		Purification	
		Amount	Form	Stap.	Deprot.	Grad.	Length
**P8-1**	4.1 mg	33 µL (1.7 eq)	30 mg/mLin DMF	2 h	–	5–80	14 min
**P8-2**	4.0 mg	34 µL (1.7 eq)	30 mg/mLin DMF	2 h	–	5–80	14 min
**P8-3**	3.9 mg	31 µL (1.7 eq)	30 mg/mLin DMF	2 h	–	5–80	14 min
**P8-4**	4.0 mg	34 µL (1.7 eq)	30 mg/mLin DMF	2 h	–	5–80	14 min
**P9-1**	4.2 mg	28 µL (1.6 eq)	30 mg/mLin DMF	2 h	–	5–80	14 min
**P9-2**	4.1 mg	29 µL (1.6 eq)	30 mg/mLin DMF	2 h	–	5–80	14 min
**P9-3**	3.9 mg	26 µL (1.6 eq)	30 mg/mLin DMF	2 h	–	5–80	14 min
**P9-4**	3.9 mg	27.6 µL (1.6 eq)	30 mg/mLin DMF	2 h	–	5–80	14 min
**P8-8**	4 mg	1.15 mg (1.0 eq)	solid	3 h	50 min	15–40	20 min
**P9-8**	4 mg	1.01 mg (1.0 eq)	solid	3 h	50 min	15–40	20 min
**P10-8**	4 mg	1.14 mg (1.0 eq)	solid	3 h	50 min	15–40	20 min
**P11-8**	4 mg	1.04 mg (1.0 eq)	solid	18 h	50 min	15–40	20 min
**P12-8**	4 mg	1.06 mg (1.0 eq)	solid	3 h	50 min	15–40	20 min
**P2-20**	4 mg	1.9 mg (1.0 eq)	solid	3 h	1 h	15–40	20 min
**P2-21**	2 mg	0.97 mg (1.0 eq)	solid	3 h	1 h	15–40	20 min

* A few drops of 2 M NaOH were added until the mixture became homogenous.

Notes: MES corresponds to MES buffer (50 mM, pH 6). "Stap." stands for stapling completion, "Deprot." stands for deprotection and "Grad." stands for gradient.

Table S9 Stapled peptide analytical data.

Peptide	Structure	LC-MS			HPLC	
		Ion	Calculated	Observed	t_R	Purity
**P1-1**	(1) Ac—TLKQFCKGVGKWCVK—NH_2	$[M + 2H]^{2+}$	1016.6 Da	1016.7 Da	8.46 min	94%
**P3-1**	(1) Ac—WCLKKFRGCF—NH_2	$[M + H]^{+}$	1594.0 Da	1594.5 Da	8.99 min	98%
**P4-1**	(1) H—RFRRLCKKWRKRCKKI—NH_2	$[M + 2H]^{2+}$	1236.1 Da	1236.3 Da	7.62 min	99%
**P5-1**	(1) C_8—GCLKFIKKCL—NH_2	$[M + H]^{+}$	1543.1 Da	1543.5 Da	9.79 min	92%
**P6-1**	(1) H—KWVQNYCKHLGRKCHTLKT—NH_2	$[M + H]^{+}$	2608.2 Da	2608.8 Da	7.84 min	82%
**P7-1**	(1) H—NLFRKLCHRLFRRCFGYTLR—NH_2	$[M + 2H]^{2+}$	1433.3 Da	1433.3 Da	8.87 min	99%
**P2-10a**	(10a) H—FCKWAFKWCKK—NH_2	$[M + 2H]^{2+}$	1167.0 Da	1167.4 Da	7.78 min	98%*
**P2-10**	(10) H—FCKWAFKWCKK—NH_2	$[M + 2H]^{2+}$	1139.5 Da	1139.3 Da	6.73 min	89%

Continued on next page

Table S9 Stapled peptide analytical data. (Continued)

Peptide	Structure	LC-MS			HPLC	
		Ion	Calculated	Observed	t_R	Purity
**P2-8a**	(8a) H—FCKWAFKWCKK—NH_2	$[M + 2H]^{2+}$	1011.0 Da	1009.5 Da	6.82 min	88%*
**P2-8**	(8) H—FCKWAFKWCKK—NH_2	$[M + 2H]^{2+}$	981.4 Da	981.4 Da	6.03 min	99.5%
**P2-12**	(12) H—FCKWAFKWCKK—NH_2	$[M - H]^{-}$	2019.0 Da	2019.7 Da	5.91 min	95%*
**P2-1**	(1) H—FCKWAFKWCKK—NH_2	$[M + H]^{+}$	1738.9 Da	1739.4 Da	6.72 min	98%
**P2-2**	(2) H—FCKWAFKWCKK—NH_2	$[M + H]^{+}$	1753.9 Da	1754.0 Da	6.94 min	99.6%
**P2-3**	(3) H—FCKWAFKWCKK—NH_2	$[M + H]^{+}$	1740.9 Da	1740.8 Da	6.54 min	99%
**P2-4**	(4) H—FCKWAFKWCKK—NH_2	$[M + H]^{+}$	1754.9 Da	1754.8 Da	6.88 min	99.6%
**P8-1**	(1) H—CAKWAFKCLKK—NH_2	$[M + 2H]^{2+}$	795.5 Da	795.7 Da	6.31 min	94%

Continued on next page

Table S9 Stapled peptide analytical data. (Continued)

Peptide	Structure	LC-MS			HPLC	
		Ion	Calculated	Observed	t_R	Purity
**P8-2**	② H—CAKWAFKCLKK—NH_2	$[M + 2H]^{2+}$	802.5 Da	802.7 Da	6.46 min	98%
**P8-3**	③ H—CAKWAFKCLKK—NH_2	$[M + 2H]^{2+}$	795.9 Da	796.4 Da	6.35 min	92%
**P8-4**	④ H—CAKWAFKCLKK—NH_2	$[M + 2H]^{2+}$	803.0 Da	803.2 Da	6.42 min	96%
**P9-1**	① H—FAKWCFKWLKKC—NH_2	$[M + 2H]^{2+}$	927.0 Da	926.8 Da	7.22 min	98%
**P9-2**	② H—FAKWCFKWLKKC—NH_2	$[M + 2H]^{2+}$	934.0 Da	933.8 Da	7.65 min	94%
**P9-3**	③ H—FAKWCFKWLKKC—NH_2	$[M + 2H]^{2+}$	927.5 Da	927.5 Da	7.17 min	97%
**P9-4**	④ H—FAKWCFKWLKKC—NH_2	$[M + 2H]^{2+}$	934.5 Da	934.3 Da	7.28 min	89%
**P8-8**	⑧ H—CAKWAFKCLKK—NH_2	$[M + 2H]^{2+}$	906.4 Da	907.1 Da	5.71 min	98%

Continued on next page

Table S9 Stapled peptide analytical data. (Continued)

Peptide	Structure	LC-MS			HPLC	
		Ion	Calculated	Observed	t_R	Purity
**P9-8**	(8)<br>H—FAKWCFKWLKKC—NH_2	$[M + 2H]^{2+}$	1038.0 Da	1038.1 Da	6.49 min	98%
**P10-8**	(8)<br>H—FAKCAFKWLKC—NH_2	$[M + 2H]^{2+}$	916.4 Da	916.7 Da	6.49 min	98%
**P11-8**	(8)<br>H—CFAKWAFCWLKK—NH_2	$[M + 2H]^{2+}$	1009.5 Da	1009.6 Da	7.15 min	98%
**P12-8**	(8)<br>H—KFAKCAFKWLKC—NH_2	$[M + 2H]^{2+}$	980.5 Da	980.7 Da	6.32 min	96%
**P2-20**	(20)<br>H—FCKWAFKWCKK—NH_2	$[M + 2H]^{2+}$	1195.6 Da	1196.2 Da	7.08 min	62%
**P2-21**	(21)<br>H—FCKWAFKWCKK—NH_2	$[M + 2H]^{2+}$	1205.5 Da	1206.2 Da	7.33 min	79%

* Purity based on UV absorbance chromatogram on LC–MS.

Circular dichroism spectroscopy

Circular dichroism spectroscopy was performed with an Aviv 410 circular dichroism spectro-polarimeter. Data was collected between 185–250 nm at 298 K with the following parameters: 1.0 nm bandwidth, 0.5 s averaging time, 1.0 nm wavelength step in a quartz cuvette with a 1 mm path length. Pure, lyophilised peptides were dissolved in either MeCN/H_2O (1:1) to an approximate concentration of 100 µL. The recorded spectra are a smoothed average of three scans, normalised against the solvent used. The ellipticity recorded was normalised against the ellipticity recorded at 207 nm, and the α-helicity calculated using the following equation, where E_{193} and E_{211} are the ellipticities recorded at these wavelengths (in nm):[9,10]

$$\alpha\, helicity = 27.58 - (14.46 \times E_{193}) + (1.86 \times E_{193}{}^{2}) - (5.66 \times E_{211}) - (14.72 \times E_{211}{}^{2})$$

6 Biological assays

Bacterial culture preparation

The bacterial strains were provided by the Welch Group. The *Pseudomonas aeruginosa* strains of PAO1 and YM64, and *Staphylococcus aureus* were used for initial susceptibility testing. For the follow-up minimal inhibitory concentration (MIC) determination, *P. aeruginosa* (strains PAO1 and PA14), *Burkholderia thailandensis* E264, *Enterococcus faecalis* ATCC29212, *Escherichia coli* DH5a, *Klebsiella pneumoniae* (CK1, Kenya/Kakamega)[11], *Serratia marcescens* ATCC39006, *Staphylococcus aureus* MRSA15 were used. Bacteria were plated from a glycerol stock on agar plates and plates incubated overnight at 37 °C. A single colony of bacteria was picked and grown in Luria-Bertani (LB-Broth Lennox, Formedium) medium overnight at 37 °C.

All compounds were stored as 1, 10 or 25.6 mg/mL stock solutions in sterilised deionised H_2O or sterilised DMSO:H_2O (1:1) mixture.

Initial susceptibility testing

Bacterial cultures were diluted to OD_{600} 0.1 in cation-adjusted Muller-Hinton medium (MH; Mueller-Hinton broth, Sigma-Aldrich). Into each well of a 96-well plate (untreated, flat bottom, Nuncleon Delta Surface Nunc F, ThermoFischer) was added 99 µL of bacterial suspension, leaving the outer columns empty in case of evaporation. To the desired wells 1 µL of compound stock solution was added. Some wells left untreated as a negative control, colistin was added to some wells as a positive control, and some wells contained only cation-adjusted MH to check for contamination of the broth. The plate was sealed with a gas-permeable membrane and incubated at 37 °C with 50 revolutions per minute (rpm) shaking for 18 h. The optical density at 600 nm (OD_{600}) was measured, and the MIC was determined as the lowest concentration of compound which prevented visible growth. Each sample was duplicated on the same plate and each test repeated in triplicate on separate days.

MIC determination using broth microdilution method

Bacterial culture was quantified by measuring the OD_{600}, medium exchanged to MH-medium and culture diluted to OD_{600} of 0.022 in MH-medium. Stock solutions of the samples were diluted to the starting concentration of 64 µg/mL in 300 µL of MH medium. The diluted samples were added to first well of 96-well microtiter plate (untreated, flat bottom, Nuncleon Delta Surface Nunc F, ThermoFischer) and diluted serially by one half. 4 µL of the diluted bacterial solution was used to inoculate the sample solutions (150 µL in each well). The plates were then incubated at 37 °C for 16 h. For each assay, sterility (broth only) and growth control (broth with bacterial inoculum, without antibiotics) were checked with six well each in each plate. The next day, OD_{600} of the microtiter plate was

measured and 15 µL of thiazolyl blue tetrazolium bromide (MTT; 1 mg/mL stock solution in water; Acros) was added to each well of the plate. The MIC was defined as the lowest concentration of the compound with a colourless well indicating no bacterial growth and the results cross-checked with OD_{600} reading. Each sample was run in triplicate on the same plate. Polymyxin B or ciprofloxacin or both were used as positive controls to ensure comparability of results across different experiments.

Drug synergism assay

Bacterial culture was quantified by measuring the OD_{600}, medium exchanged to MH-medium and culture diluted to OD_{600} of 0.022 in MH-medium. Stock solution of compound A was diluted to twice the starting concentration in MH medium. 75 µL of the solution was added to the first row of a 96-well microtiter plate (untreated, flat bottom) and diluted serially by one half going down the rows, except for the last row which was filled with 75 µL of medium only. In a separate 96-well plate, 100 µL of a solution of compound B diluted to twice the starting concentration in MH medium was added to the first column, and diluted serially by one half going across the columns, except for the last column which was filled with 100 µL of medium only. From each well in this second plate, 75 µL were transferred to the corresponding cell in the first plate. After that, 4 µL of the diluted bacterial solution was used to inoculate the sample solutions (150 µL in each well). The plates were then incubated at 37 °C for 16 h. The next day, OD_{600} of the microtiter plate was measured and 15 µL of thiazolyl blue tetrazolium bromide (MTT; 1 mg/mL stock solution in water) was added to each well of the plate to cross-check the OD_{600} readings.

β-Lactamase assessment – co-incubation with nitrocefin

Bacterial culture was quantified by measuring the OD_{600}, medium exchanged to MH-medium and culture diluted to OD_{600} of 0.022 in MH-medium. Nitrocefin (Abcam, ab145625) as a 10 mM stock solution in DMSO was diluted in MH medium to 100 µM. 150 µL of the solution was added to 96-well plate (3 wells per bacterium) as well as 150 µL of MH medium as a negative control (further 3 wells per bacterium). The media were inoculated with 4 µL of the diluted bacterial solution and incubated in a plate reader at 37 °C for 16 h and absorption at 482 nm (Abs_{482}) and 600 nm (OD_{600}) was continuously measured throughout the incubation. Increase in Abs_{482} corresponded to hydrolysis of nitrocefin.

β-Lactamase assessment – analysis of growth medium at different time points

Bacterial culture was quantified by measuring the OD_{600}, medium exchanged to MH-medium and culture diluted to OD_{600} of 0.022 in MH-medium. 267 µL were added to 10 mL of MH-medium and medium incubated at 37 °C. At 2.5 h, 5 h, 7 h, 9 h, 11 h and 21.5 h since the beginning of incubation, 500 µL of the culture was taken, centrifuged

for 2 min at 7500 rpm and 75 µL of the supernatant were transferred to a multi-well plate. 75 µL of 0.2 mM solution of nitrocefin in MH-medium was added and the kinetics of its degradation measured for 1.5 h to 2 h by following Abs_{482}. The initial rate of change in Abs_{482} was adjusted for the dilution factor of 2 and using a calibration curve (Figure S6c) converted to rate of change in nitrocefin concentration. This data were further used to estimate the equivalent amount of recombinant β-lactamase that would achieve the same initial rate (Figure S6d).

Fitting of growth curves

Data from the continuous monitoring of OD_{600} during bacterial growth were fitted with a line following a Baranyi and three-phase linear models[2,12,13]. Data were fitted using the `curve_fit` function from Python SciPy package (version 1.12.0). The data were plotted using Matplotlib package (Figure S4). The following equations were used for fitting:

Baranyi:

$$y(t) = y_0 + \mu A(t) - ln(1 + \frac{e^{\mu A(t)} - 1}{e^C})$$
$$\text{where } A(t) = t + \frac{1}{\mu} ln(e^{-\mu t} + e^{-\mu\lambda} - e^{-\mu(t+\lambda)})$$

Three-phase linear:

$$y(t) = y_0 \text{ for } t \leq \lambda$$
$$y(t) = y_0 + C\frac{t-\lambda}{t_{stat}-\lambda} \text{ for } \lambda < t < t_{stat}$$
$$y(t) = y_0 + C \text{ for } t \geq t_{stat}$$

$y(t)$ stands for ln of OD_{600} or ln of the cell titre, y_0 for the ln of the initial OD_{600}, C for the difference between ln of the maximum OD_{600} (y_{max}) and y_0, μ for the growth rate, λ for the growth lag time and t_{stat} for the time when the exponantial growth phase turns into stationary phase.

Calibration curves for β-lactamase activity

To obtain the calibration curve for nitrocefin, a series of solutions of nitrocefin in MH-medium with concentrations varying from 10 µM to 160 µM was prepared and 75 µL were added to a multi-well plate. 75 µL of 2 nM solution of recombinant β-lactamase (TEM-1 expressed in *E. coli*; manufactured by Prospec; ENZ-351) in MH-medium were added and Abs_{482} was followed over 2 h. The Abs_{482} of the maximal plateau value was obtained from the curves and the values plotted against the concentrations of nitrocefin to obtain the calibration curve.

To obtain the calibration curve for β-lactamase, a serial dilution of the recombinant β-lactamase was prepared in a multi-well plate, ending with 75 µL of the solutions in

MH-medium with concentrations varying from 2 nM to 977 fM. 75 µL of 0.2 mM solution of nitrocefin in MH-medium were added and Abs_{482} was followed over 2 h. The initial rate of increase of Abs_{482} was obtained from the curves and the values plotted against the concentrations of β-lactamase to obtain the calibration curve.

β-Lactamase activity by HPLC monitoring

To 196 µL of 1 mM recombinant β-lactamase in PBS buffer (pH 7.4, 50 mM) was added 0.8 µL of 15 mg/mL solution of caffeine in water and 4 µL of 10 mg/µL solution of peptide **P2-10** in water. The mixture was incubated at 37 °C. 20 µL aliquots of the mixture were taken at different time points and directly analysed by HPLC (10 µL injection volume; AdvanceBio Peptide 2.7 µm 4.6×150 mm column; gradient of 5–95%B over 15 min). The absorption at 280 nm was used to monitor cleavage of ciprofloxacin.

Serum stability of peptides

To 400 µL of MES buffer (pH 6, 50 mM) was added 100 µL human serum (Sigma-Aldrich, H4522) and 2 µL of 15 mg/mL solution of caffeine in water. The mixture was equilibrated at 37 °C for 15 min and 10 µL of 10 mg/mL or 4 µL of 10 mM stock solution of a peptide were added. The mixture was incubated at 37 °C and 50 µL aliquots were taken at different time points. The aliquots were added to 100 µL of 1:1 DMSO:ethanol mixture and the crashed-out protein centrifuged at 13 400 rpm for 10 min. 20 µL of the supernatant were analysed using HPLC. The absorption at 220 nm was used to monitor stability of peptides.

Mammalian cell culture preparation

Immortalised human epithelial kidney cells HEK293FT were cultured in DMEM, high glucose, pyruvate (Gibco™, 41966029), supplemented with 10% (v/v) HI-FBS (Gibco™, A5209502), L-glutamine (2 mM), penicillin (50 U/mL), and streptomycin (50 µg/mL). Cells were incubated at 37 °C with 5% CO_2 and were propagated approximately every 3 days (at 80% confluency).

Sample preparation for cell viability assay

Compound **P2-10**, 10 mg/mL in sterile water, was diluted in DMEM to achieve a 2× top concentration of 256 µg/mL (final drug concentration during treatment 128 µg/mL). Lower concentrations were obtained via serial dilution. The same amount of sterile water, without the peptide, diluted in media was used as a negative control.

Sodium dodecyl sulphate (SDS) was used as a positive control in the studies. Stock solution of 20% (w/v) SDS in water was diluted in media to obtain a 2% solution. This was further diluted serially to obtain a 2× 0.05% solution (final concentration during treatment 0.025%).

Cell viability assay

HEK293FT cells were detached using trypsin-EDTA 0.25% (Gibco™, 25200056) and were seeded in 96-well plates (Corning, white, clear bottom) at 8000 cells/well for the negative control to be 80% confluent at the endpoint of the study. After 24 h, half of the media (100 µL) was replaced with a 2× solution of the respective drug in media (or positive control SDS or negative control sterile water) and the plates were shaken carefully. After a further 24 h, cell viability was determined using CellTiter-Glo® cell viability assay according to manufacturer's instructions. Briefly, both the reagents and the cells were equilibrated to room temperature and 100 µL of cell media was removed from each well. Then, 100 µL of CellTiter-Glo® reagent was added to the remaining 100 µL of media and cells in each well. The plates were protected from light and shaken on an orbital shaker for 2 min, then incubated for a further 10 min at room temperature. The luminescent output was recorded using a PHERAstar FS plate reader. The cell viability was calculated relative to the negative control (sterile water) set at 100%. Data was obtained in three independent biological repeats, performed on separate passages of cells and on separate days, each containing 3 replicates. The means are presented with standard deviation on the graphs.

7 NMR spectra

N-(3-Phenylpropyl)-4,6-divinylpyrimidin-2-amine (1)

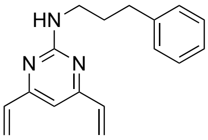

^{1}H NMR

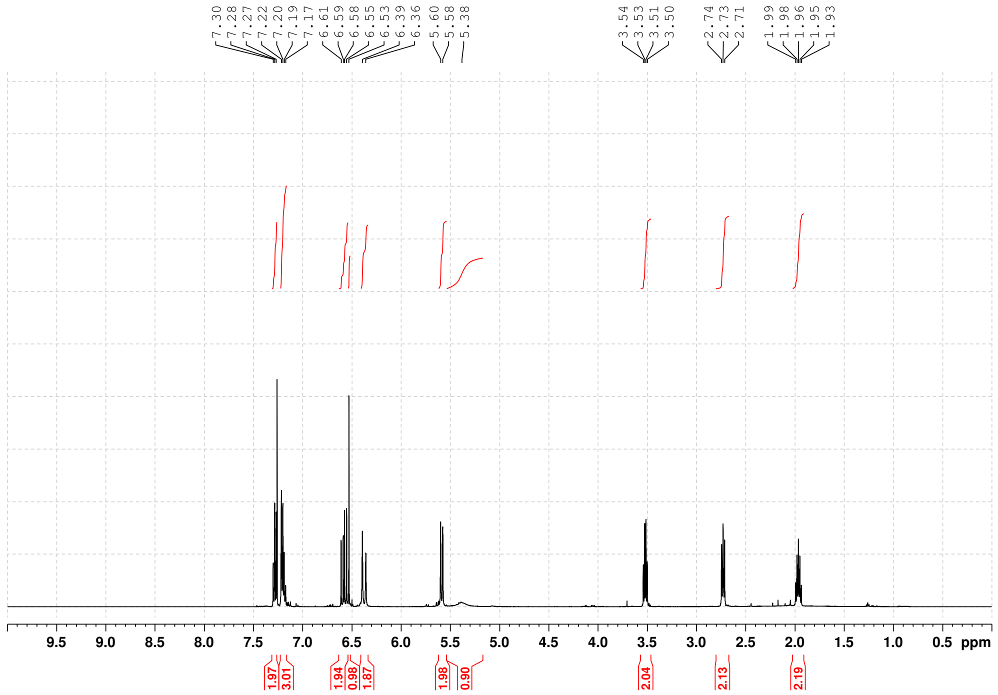

^{13}C NMR

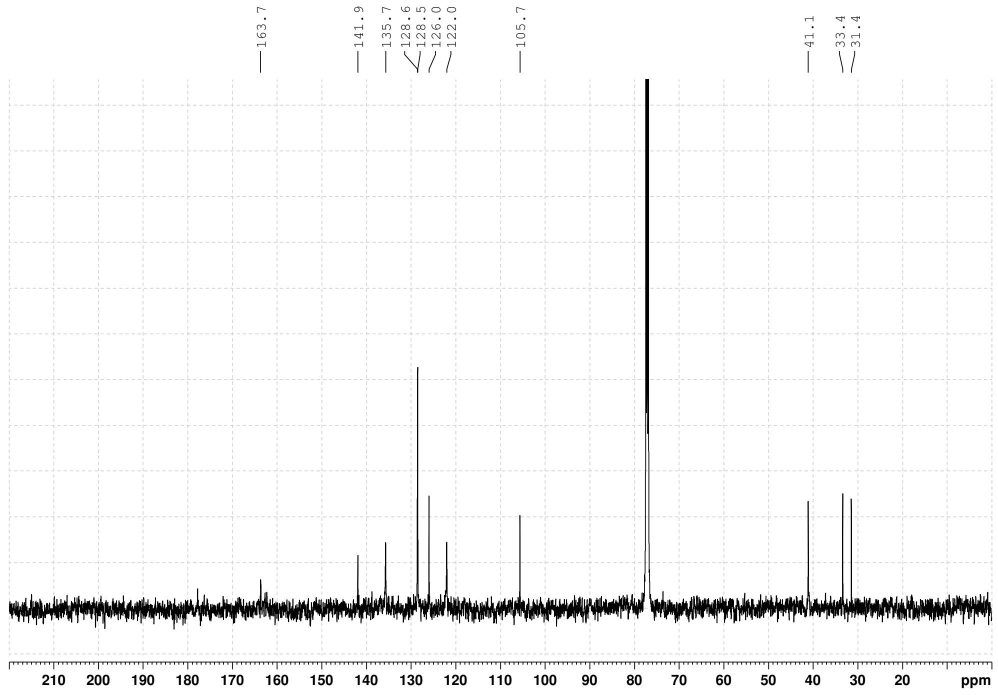

^{1}H-^{13}C HMBC

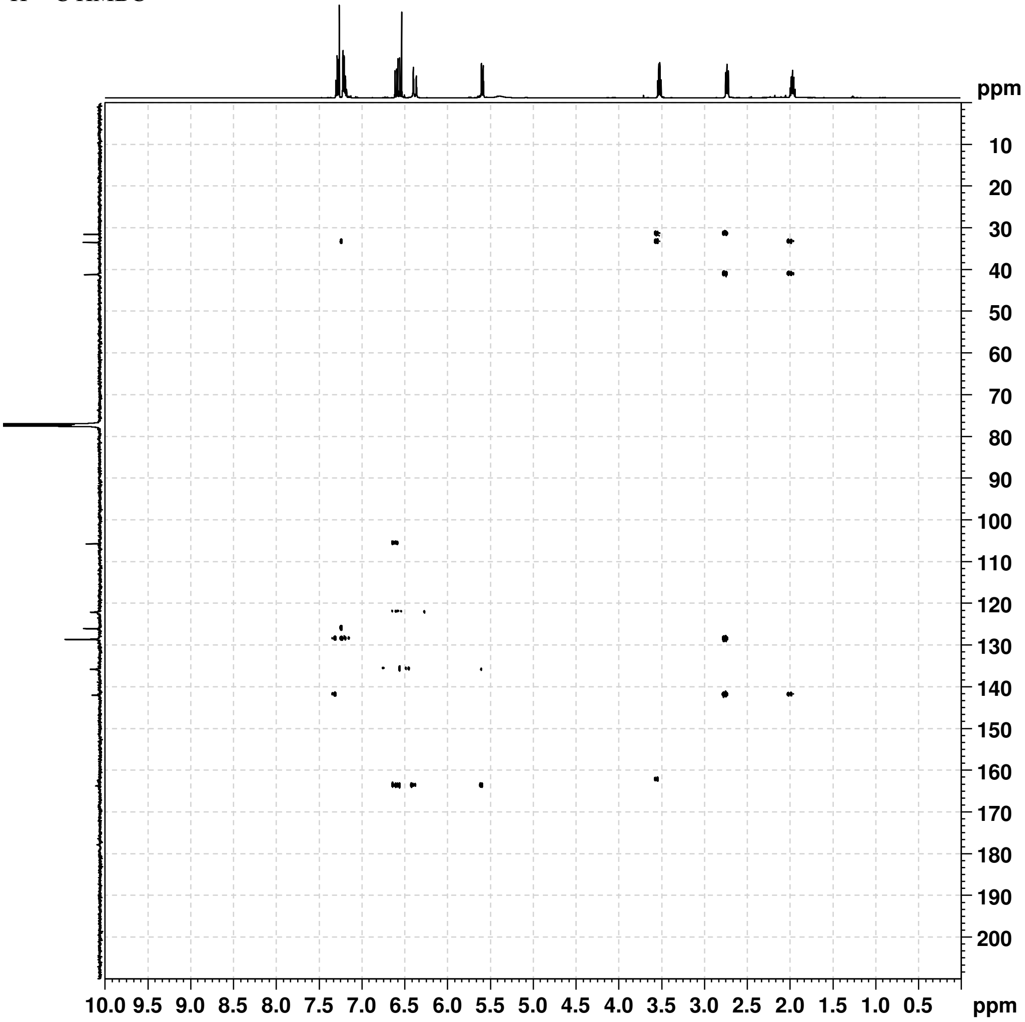
ppm
10
20
30
40
50
60
70
80
90
100
110
120
130
140
150
160
170
180
190
200
10.0 9.5 9.0 8.5 8.0 7.5 7.0 6.5 6.0 5.5 5.0 4.5 4.0 3.5 3.0 2.5 2.0 1.5 1.0 0.5
ppm

***N*-Methyl-*N*-(3-phenylpropyl)-4,6-divinylpyrimidin-2-amine (2)**

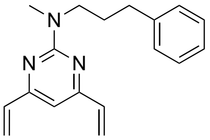

^{1}H NMR

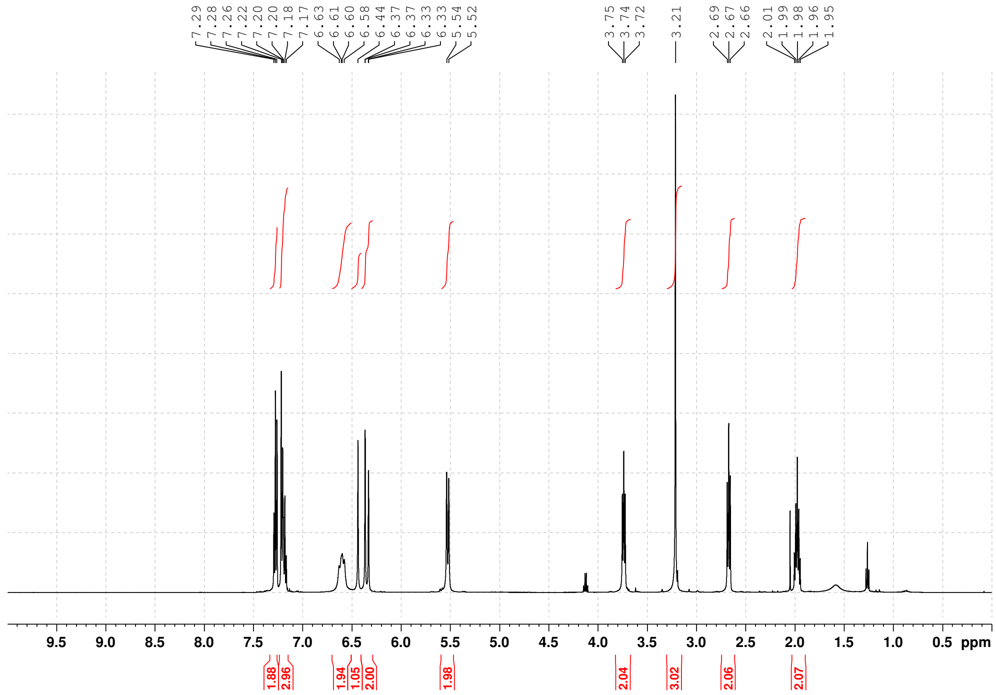

^{13}C NMR

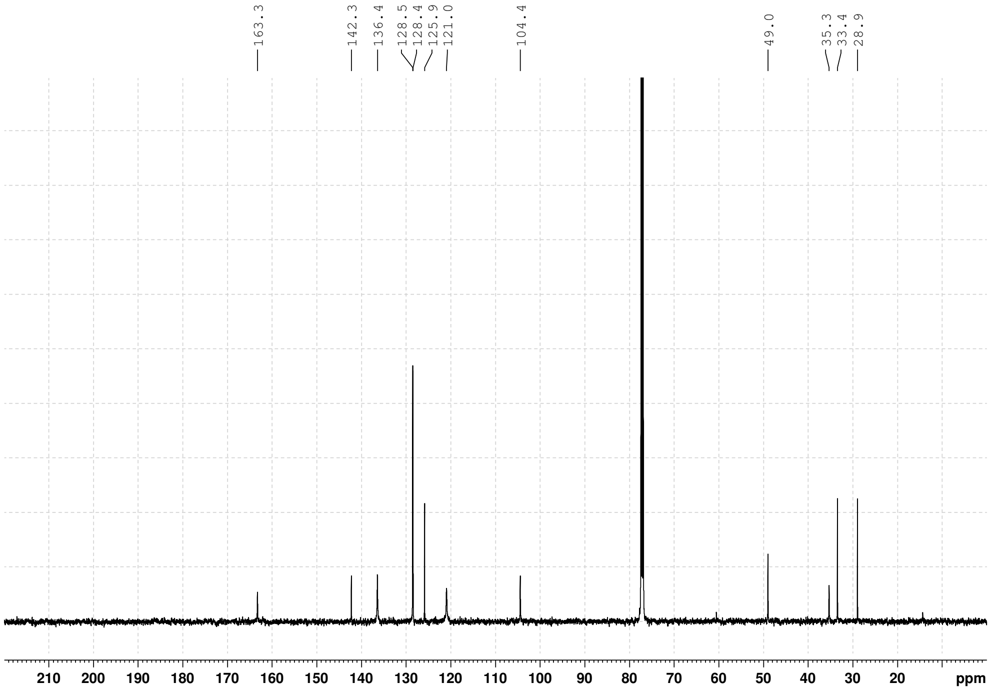

^{1}H-^{13}C HMBC

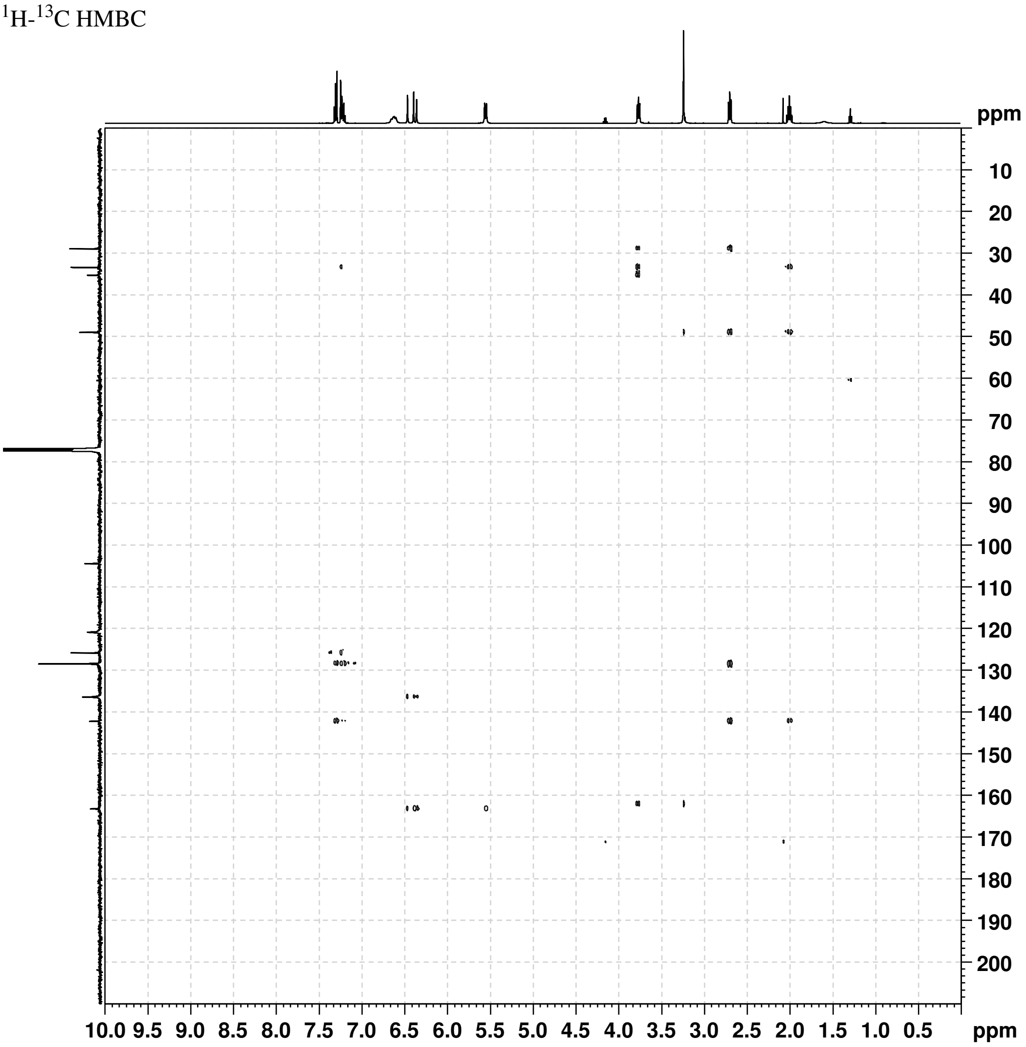

ppm
10
20
30
40
50
60
70
80
90
100
110
120
130
140
150
160
170
180
190
200
10.0 9.5 9.0 8.5 8.0 7.5 7.0 6.5 6.0 5.5 5.0 4.5 4.0 3.5 3.0 2.5 2.0 1.5 1.0 0.5
ppm

N-(3-Phenylpropyl)-4,6-divinyl-1,3,5-triazin-2-amine (3)

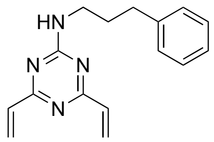

^{1}H NMR

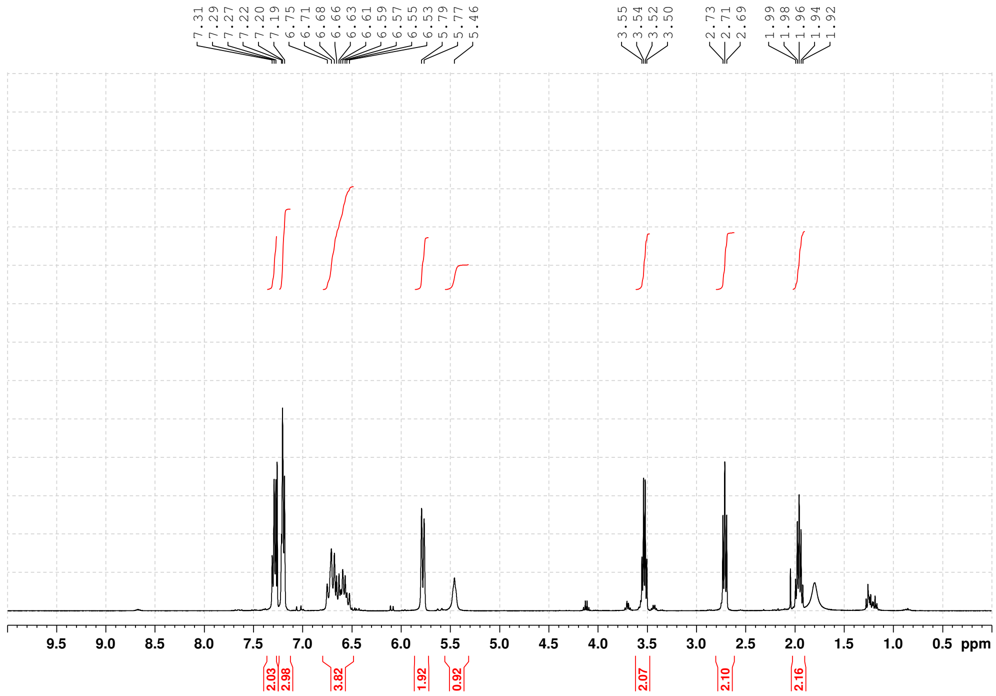

^{13}C NMR

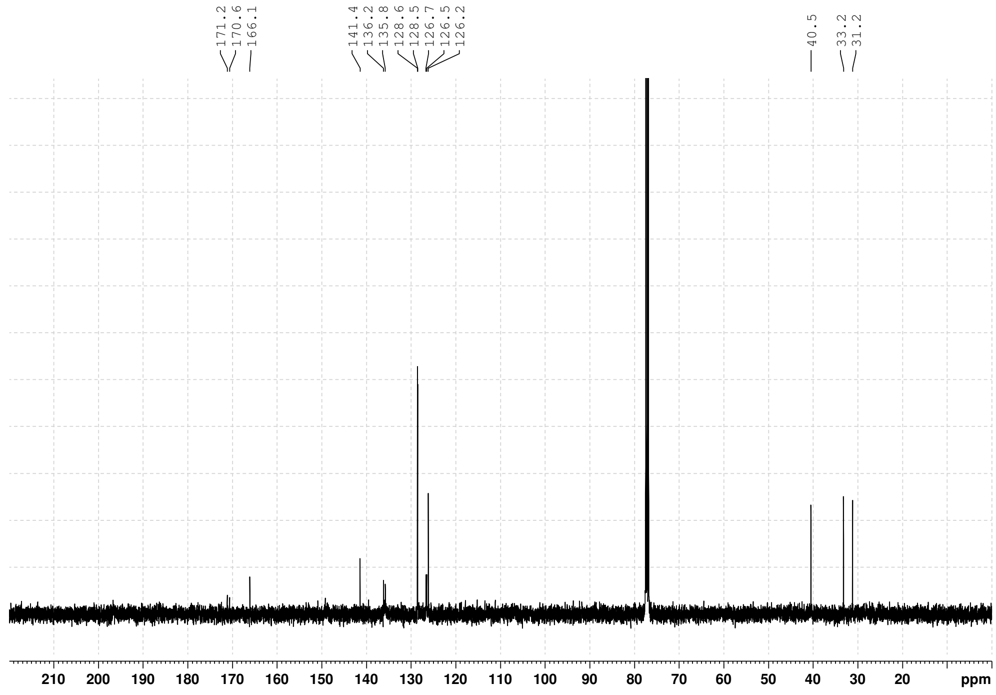

***N*-Methyl-*N*-(3-phenylpropyl)-4,6-divinyl-1,3,5-triazin-2-amine (4)**

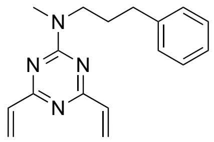

^{1}H NMR

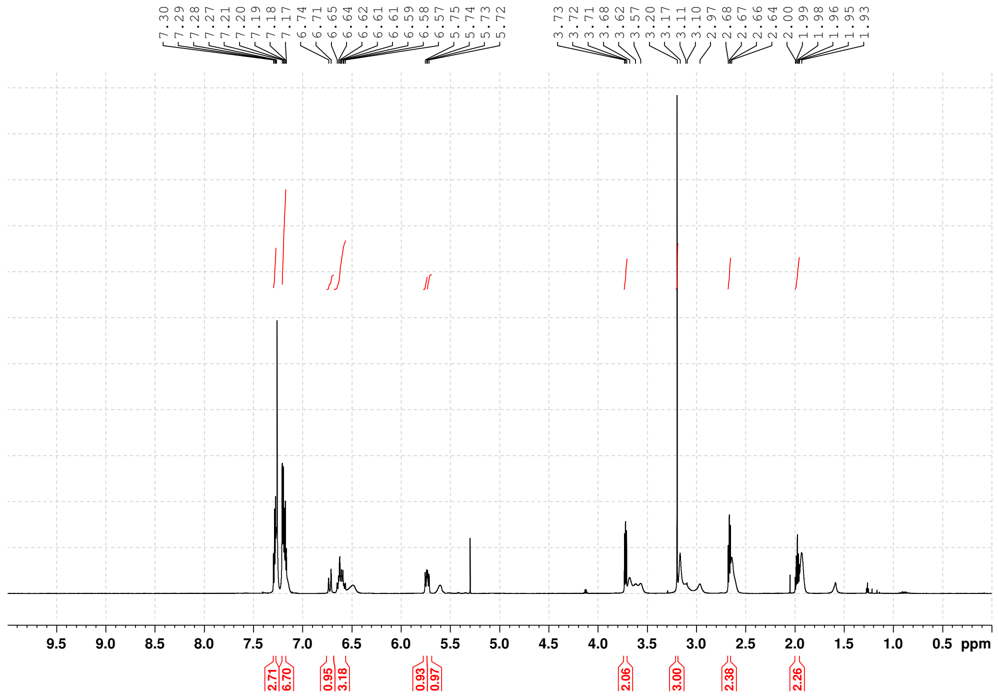

^{13}C NMR

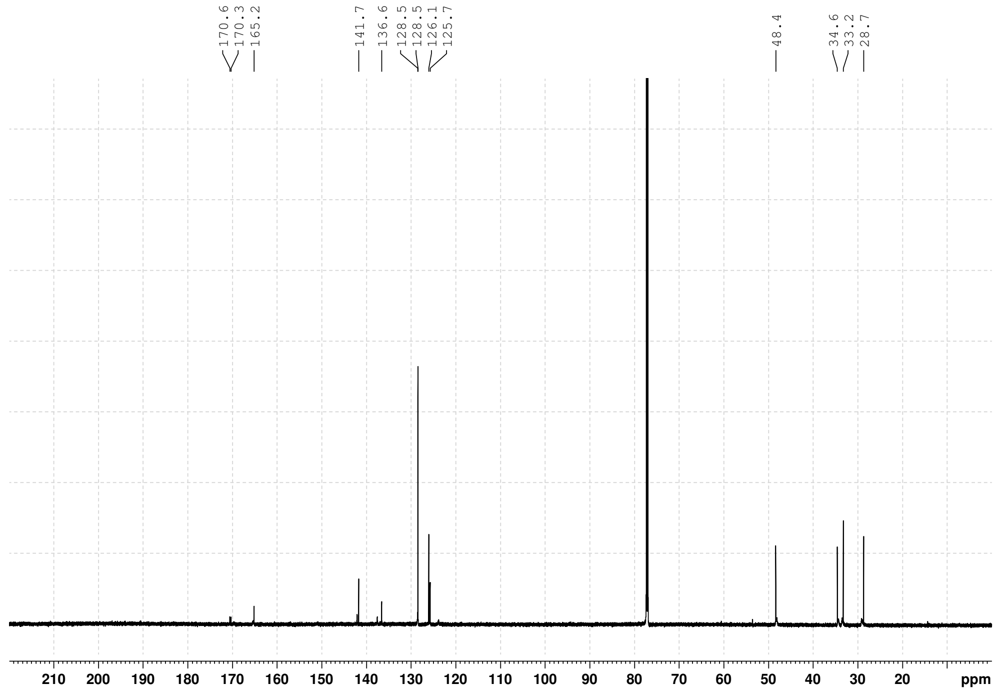

tert-Butyl (6R,7R)-3-(acetoxymethyl)-7-amino-8-oxo-5-thia-1-azabicyclo[4.2.0]oct-2-ene-2-carboxylate (6)

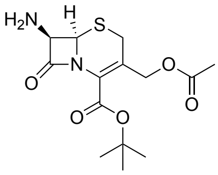

^{1}H NMR

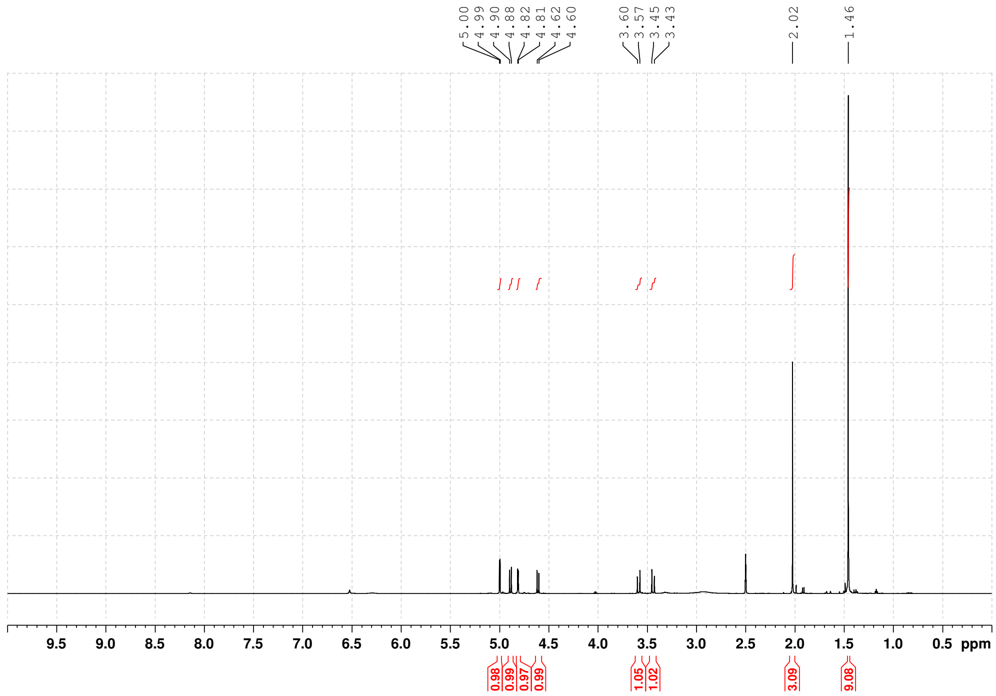

^{13}C NMR

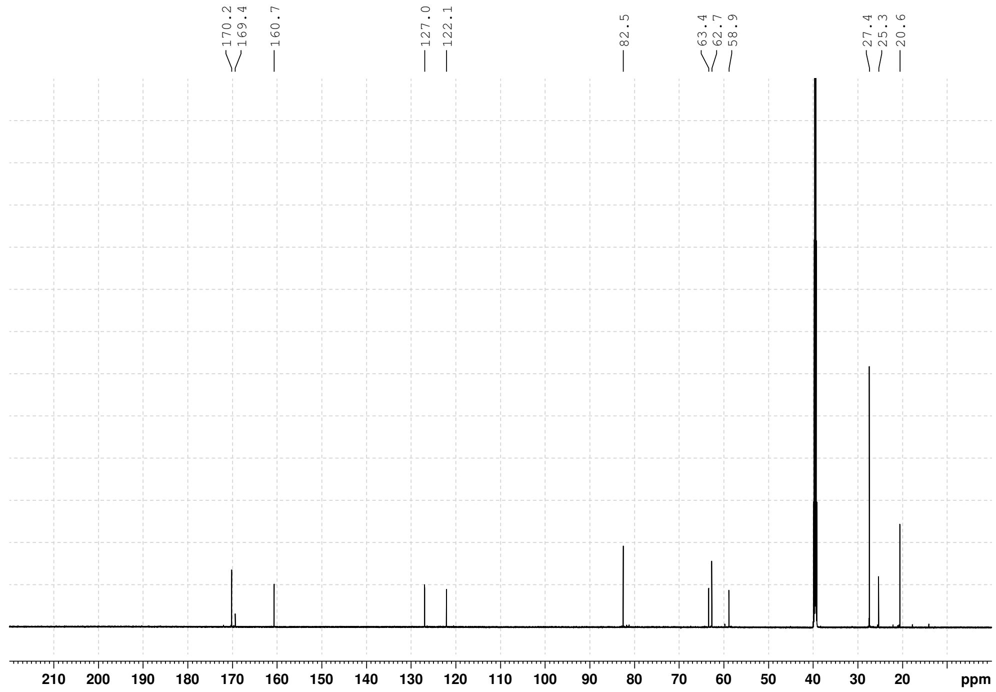

4-((4,6-Divinylpyrimidin-2-yl)amino)butanoic acid (7)

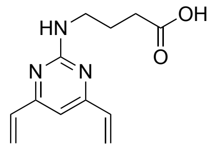

^{1}H NMR

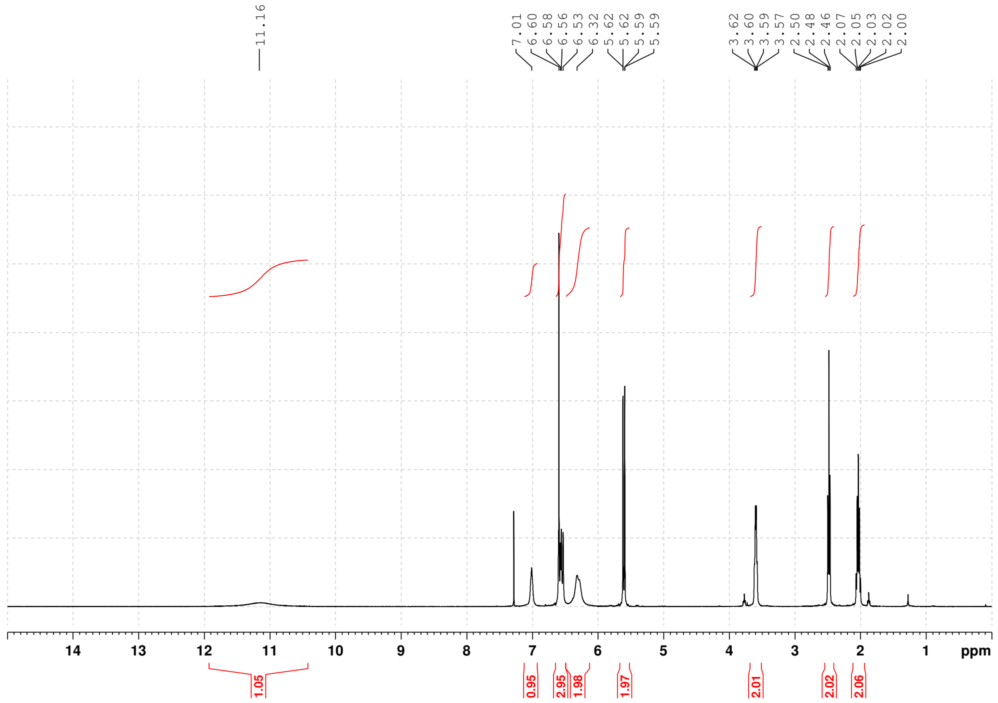

^{13}C NMR

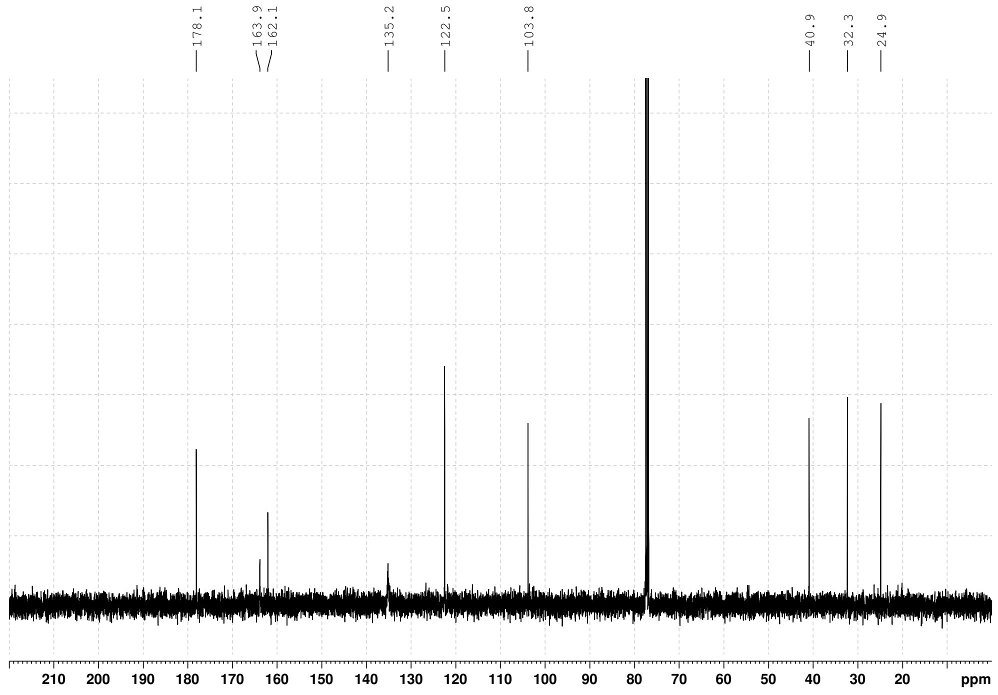

tert-Butyl (6*R*,7*R*)-3-(acetoxymethyl)-7-(4-((4,6-divinylpyrimidin-2-yl)amino)butanamido)-8-oxo-5-thia-1-azabicyclo[4.2.0]oct-2-ene-2-carboxylate (8)

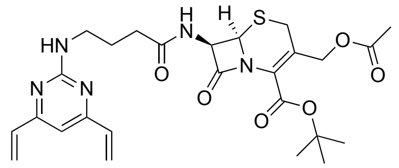

^{1}H NMR

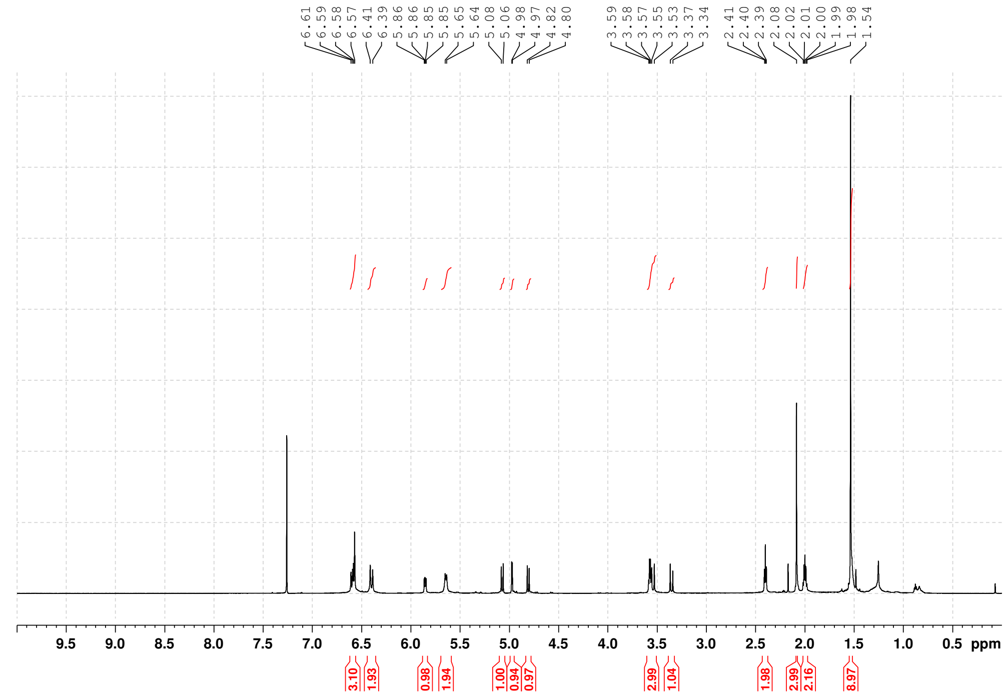

^{13}C NMR

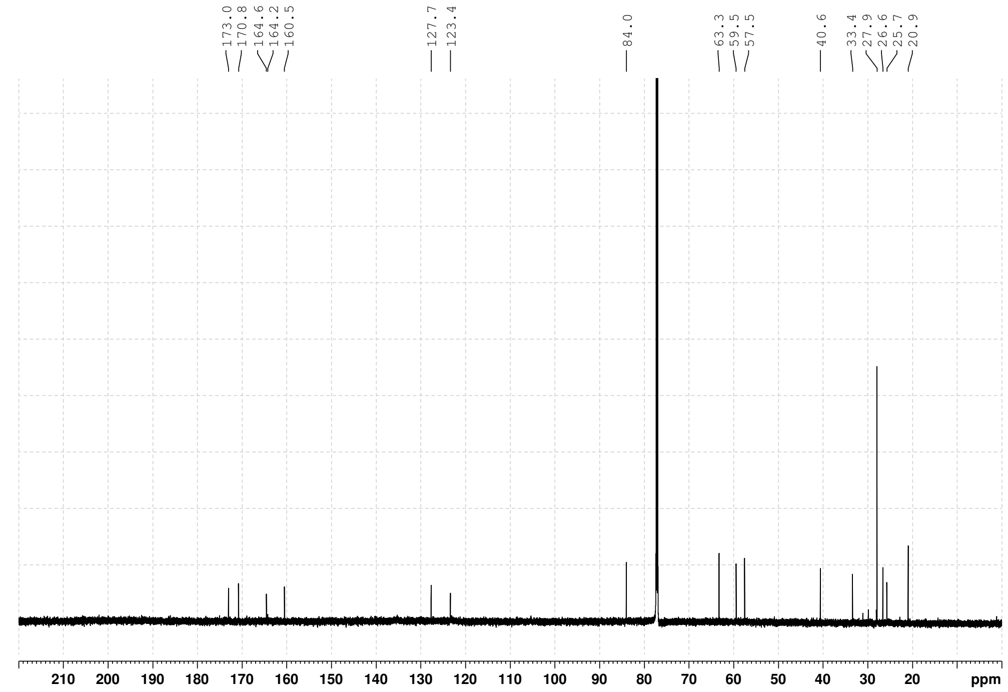

(6*R*,7*R*)-2-(*tert*-Butoxycarbonyl)-7-(4-((4,6-divinylpyrimidin-2-yl)amino)butanamido)-8-oxo-5-thia-1-azabicyclo[4.2.0]oct-2-en-3-yl)methoxy)carbonyl)piperazin-1-yl)-1-cyclopropyl-6-fluoro-4-oxo-1,4-dihydroquinoline-3-carboxylic acid (10)

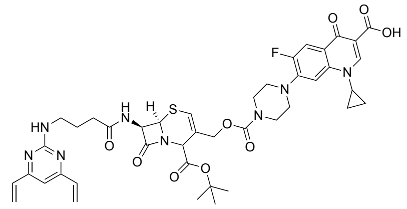

^{1}H NMR

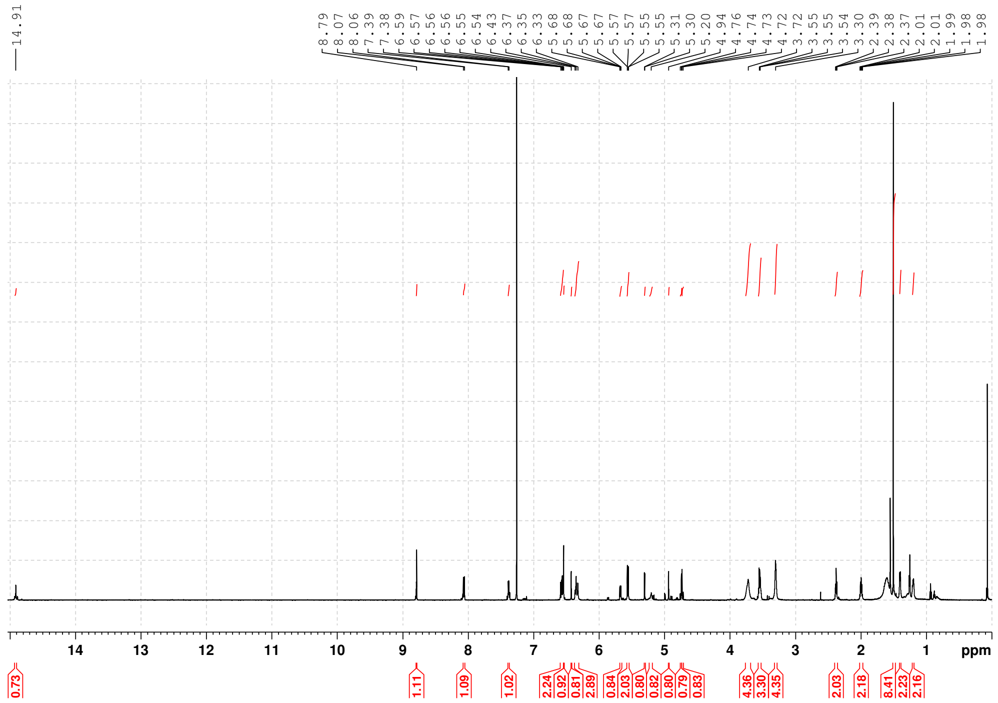

^{13}C NMR

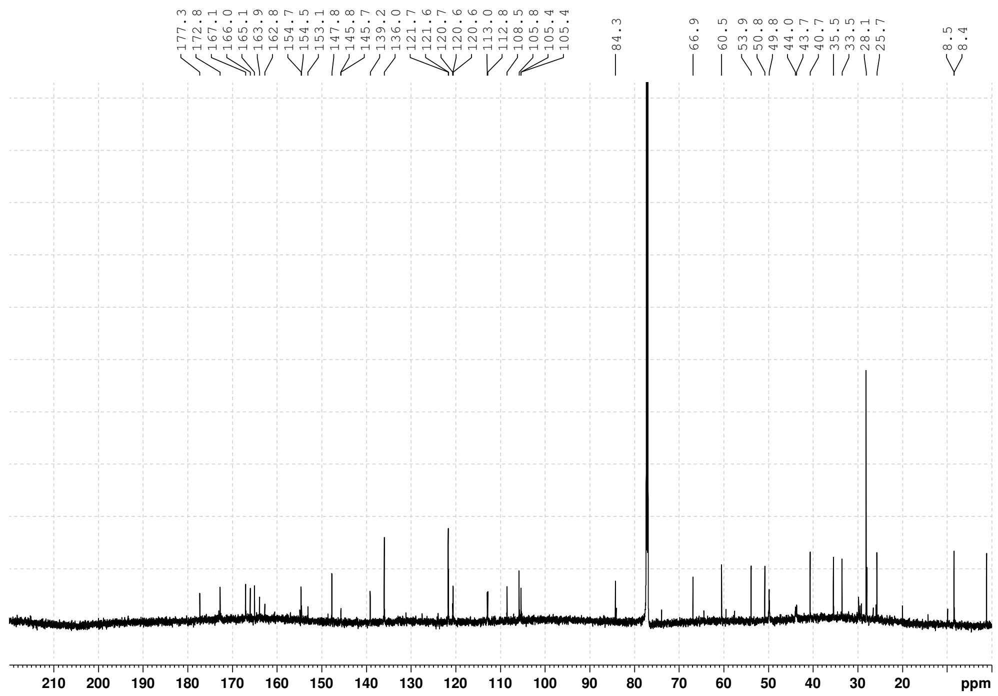

^{19}F NMR

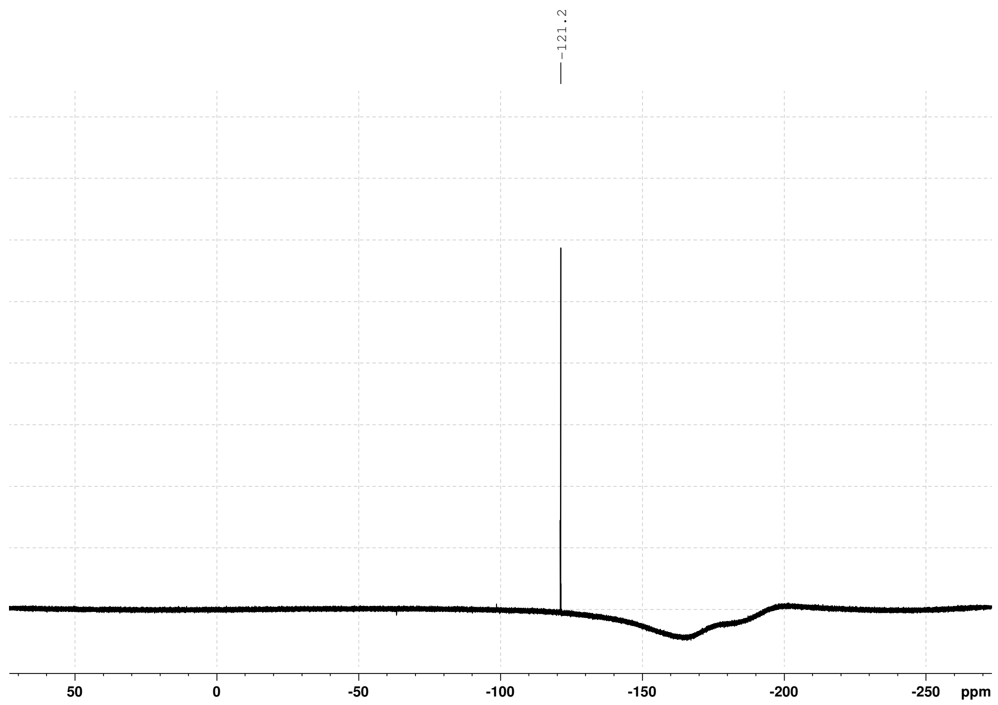

Ethyl 1-cyclopropyl-6-fluoro-4-oxo-7-(piperazin-1-yl)-1,4-dihydroquinoline-3-carboxylate (11)

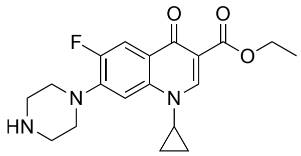

^{1}H NMR

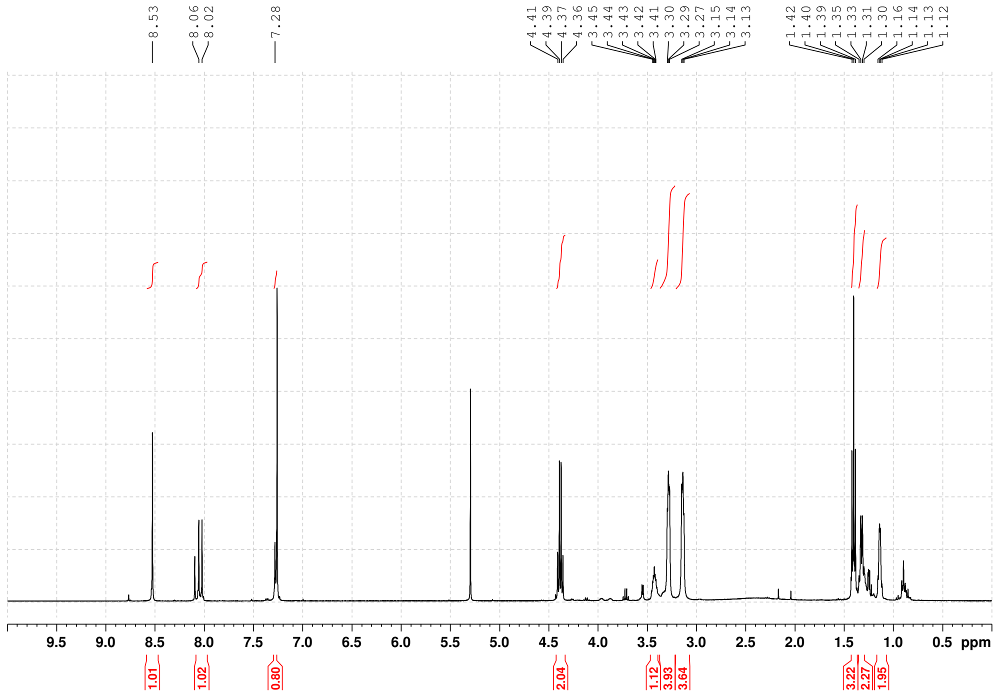

^{13}C NMR

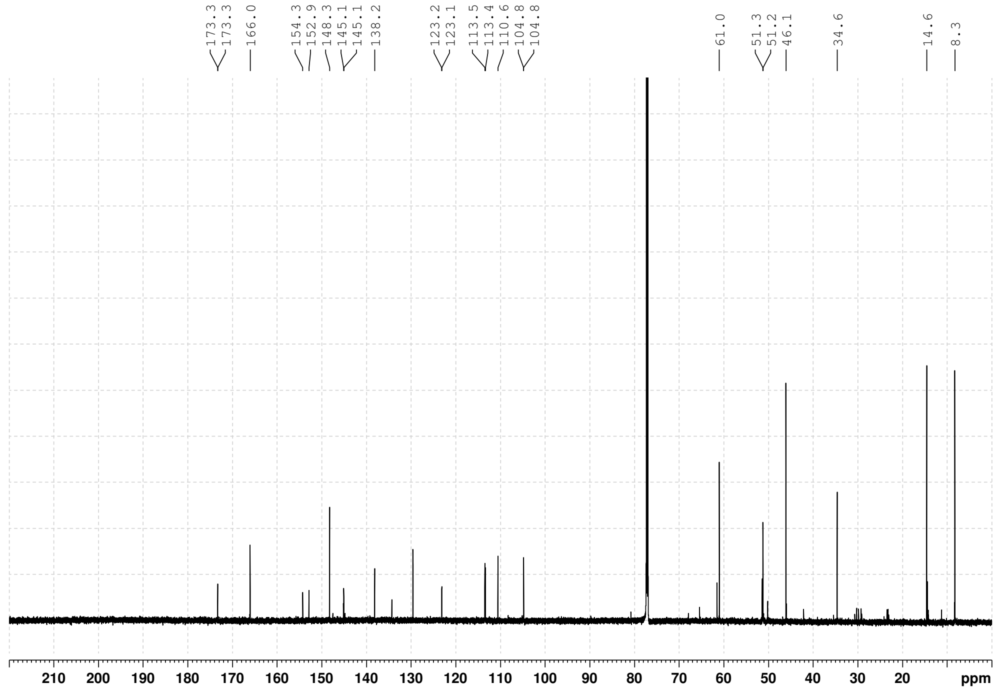

^{19}F NMR

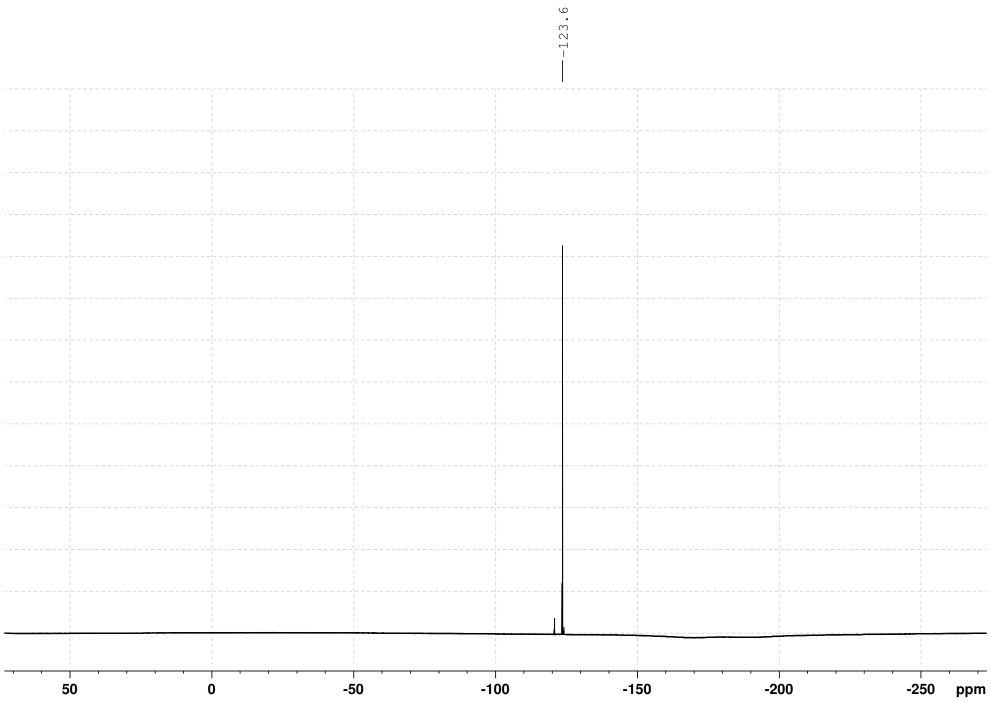

1-Cyclopropyl-7-(4-(4-((4,6-divinylpyrimidin-2-yl)amino)butanoyl)piperazin-1-yl)-6-fluoro-4-oxo-1,4-dihydroquinoline-3-carboxylic acid (12)

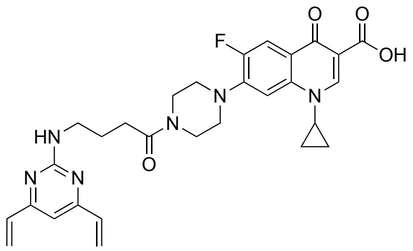

^{1}H NMR

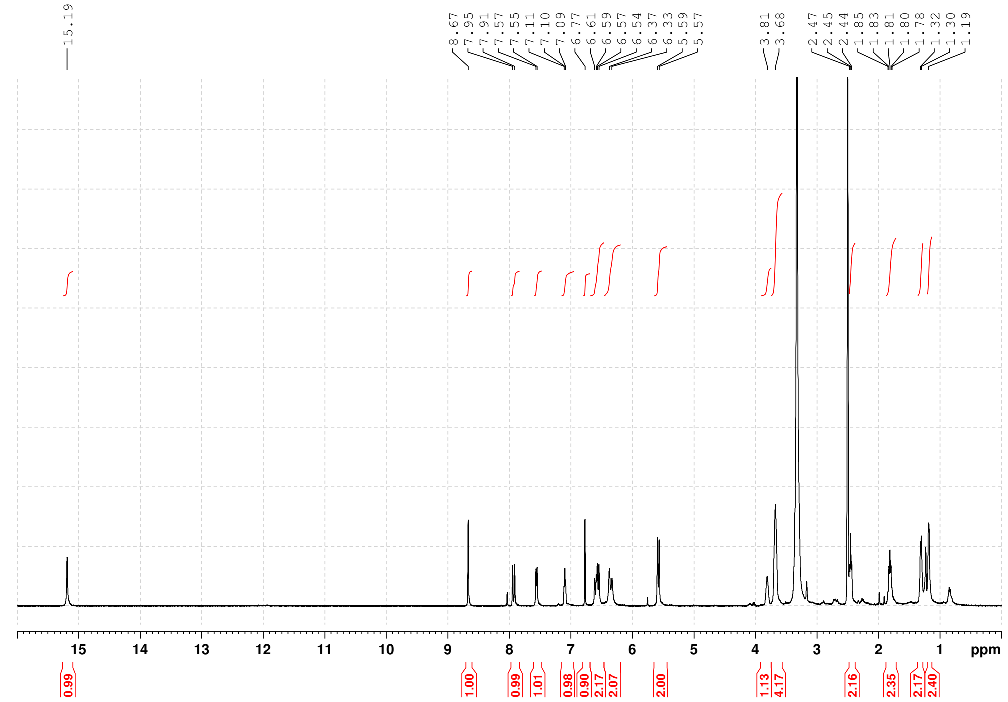

^{13}C NMR

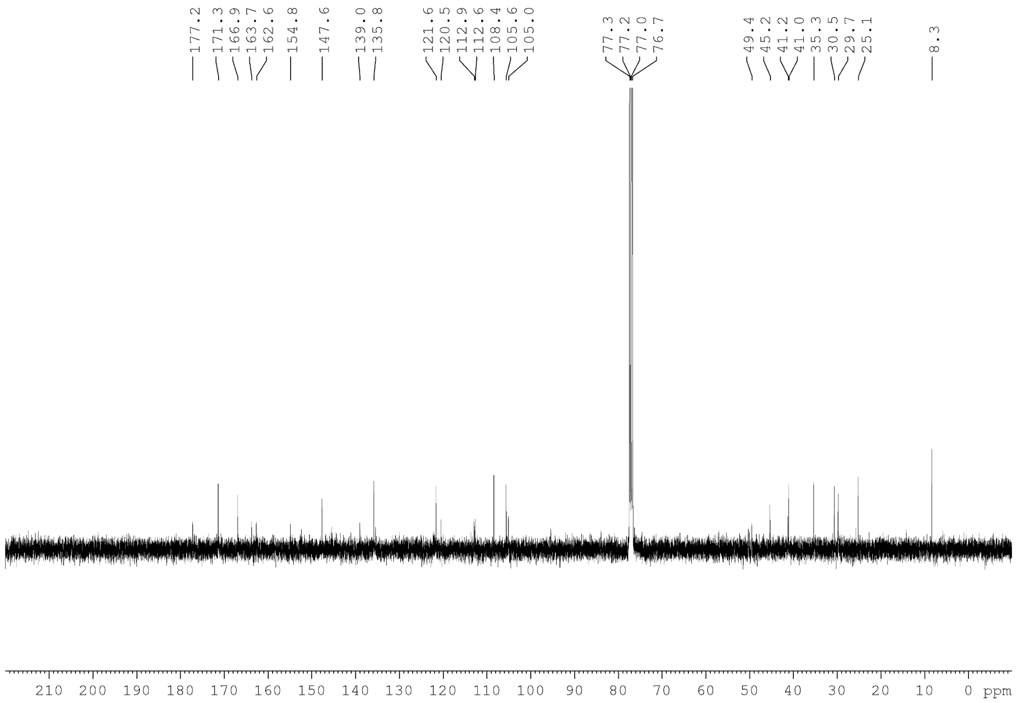

^{19}F NMR

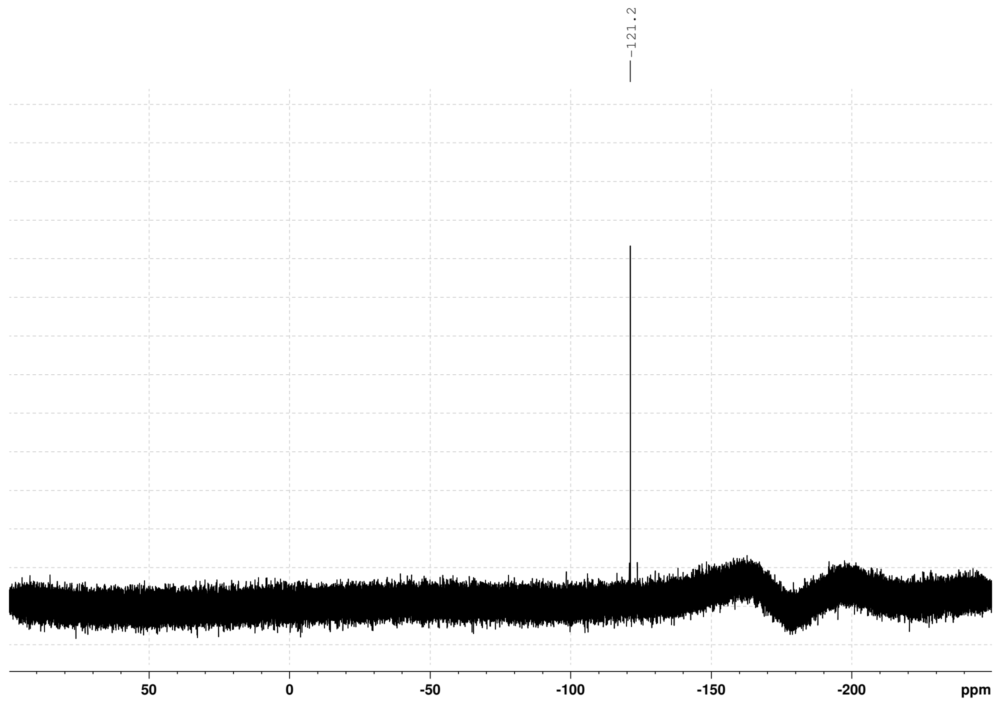

Ethyl 4-((4,6-dichloropyrimidin-2-yl)amino)butanoate (13)

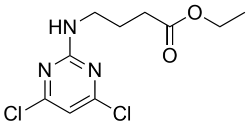

^{1}H NMR

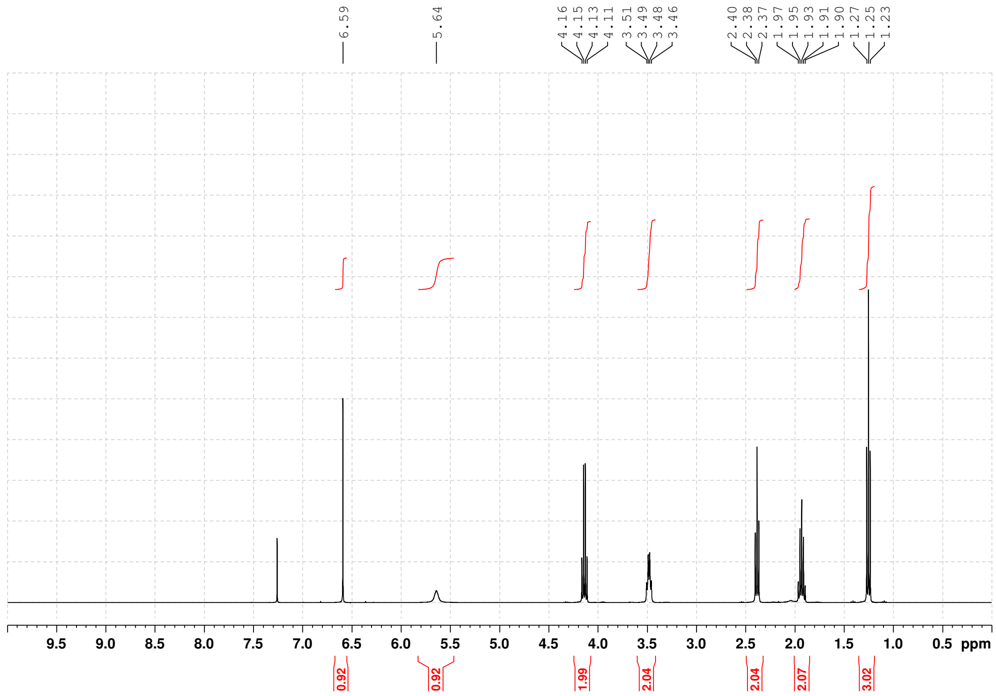

^{13}C NMR

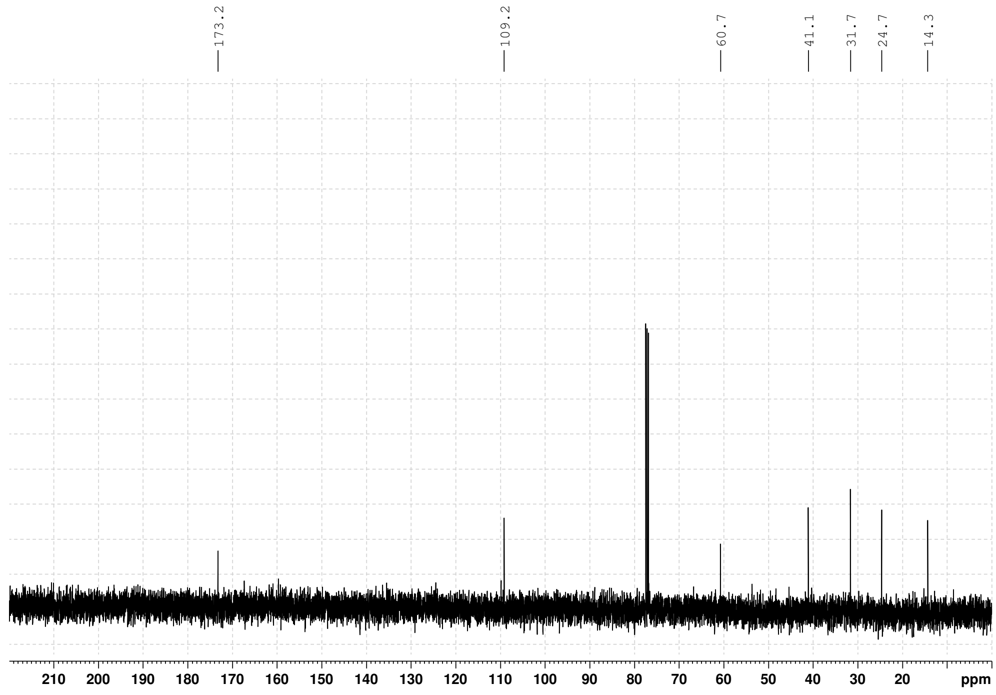

Ethyl 4-((4,6-divinylpyrimidin-2-yl)amino)butanoate (14)

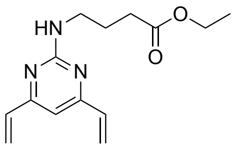

^{1}H NMR

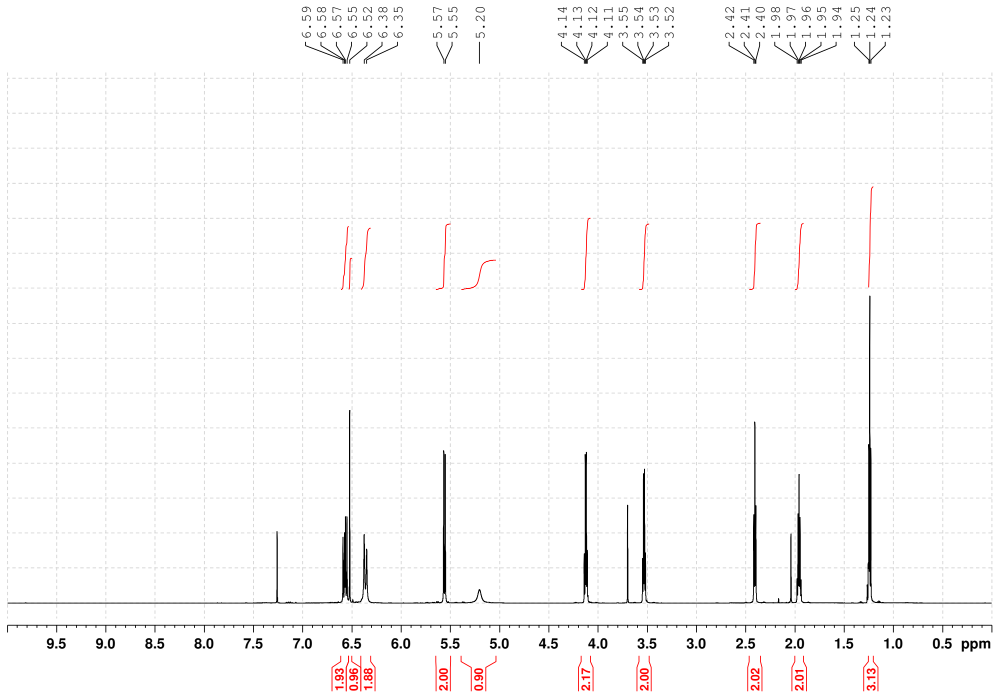

^{13}C NMR

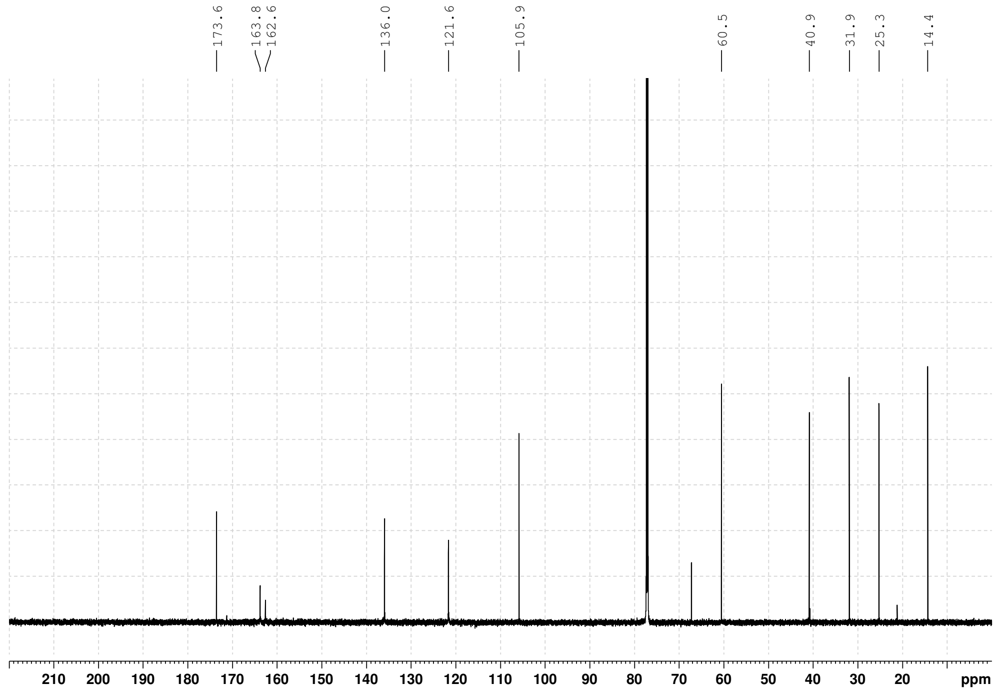

N-Methyl-3-phenylpropan-1-amine (15)

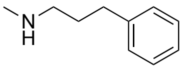

^{1}H NMR

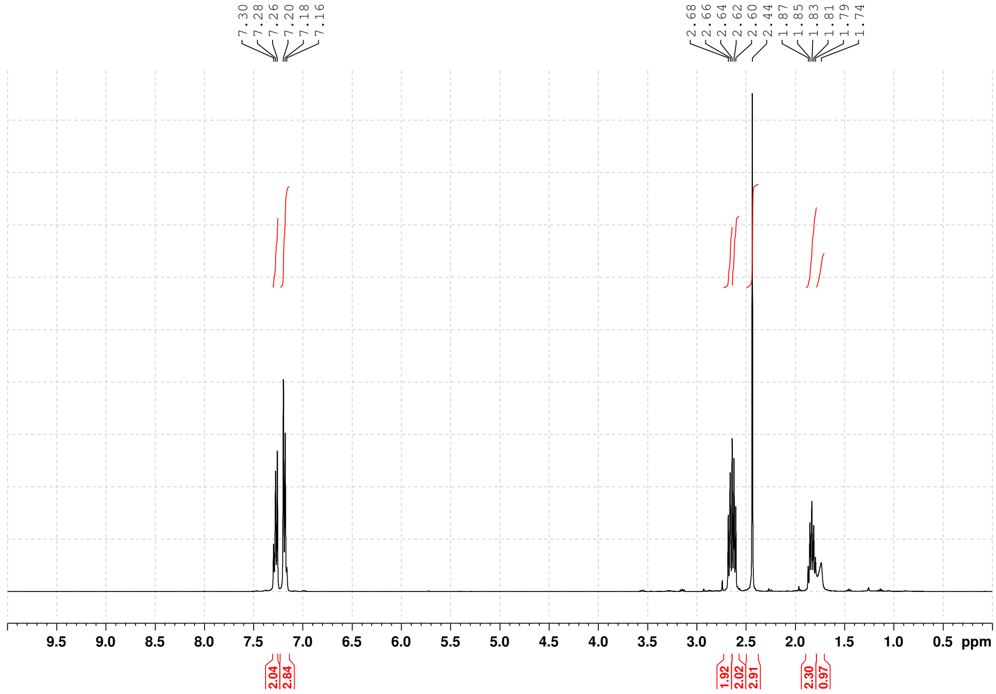

^{13}C NMR

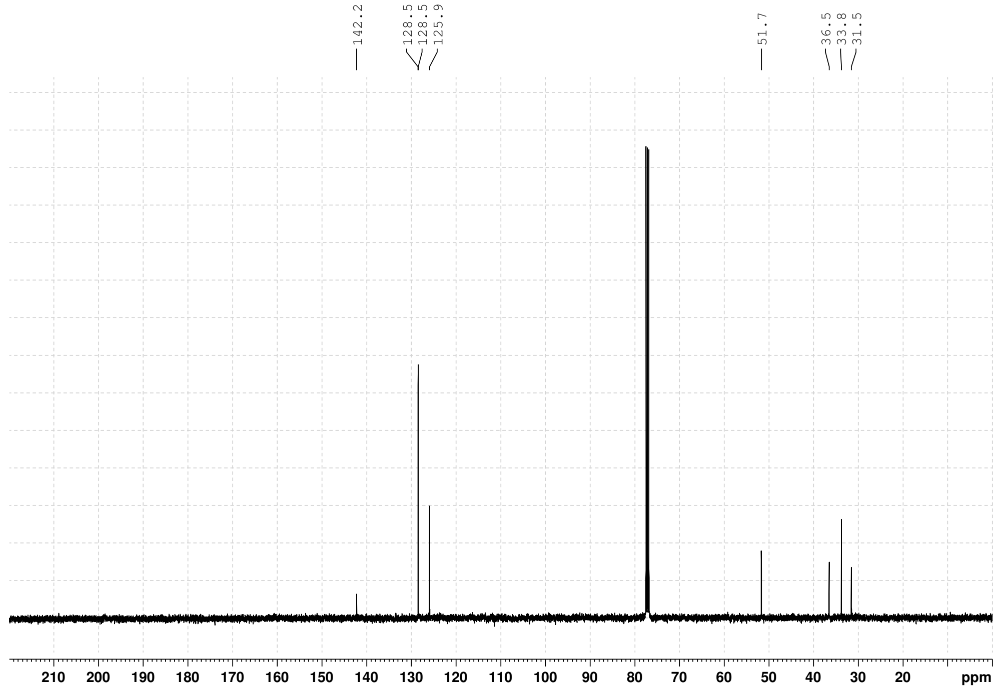

4,6-Dichloro-*N*-(3-phenylpropyl)pyrimidin-2-amine (16)

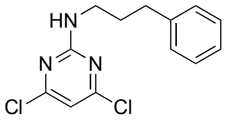

^{1}H NMR

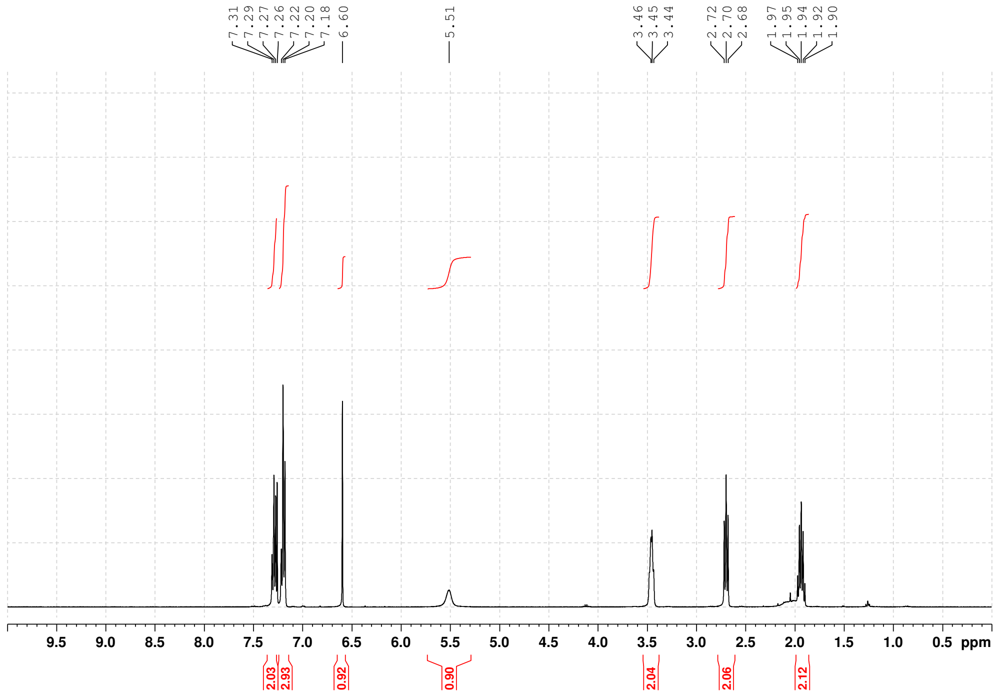

^{13}C NMR

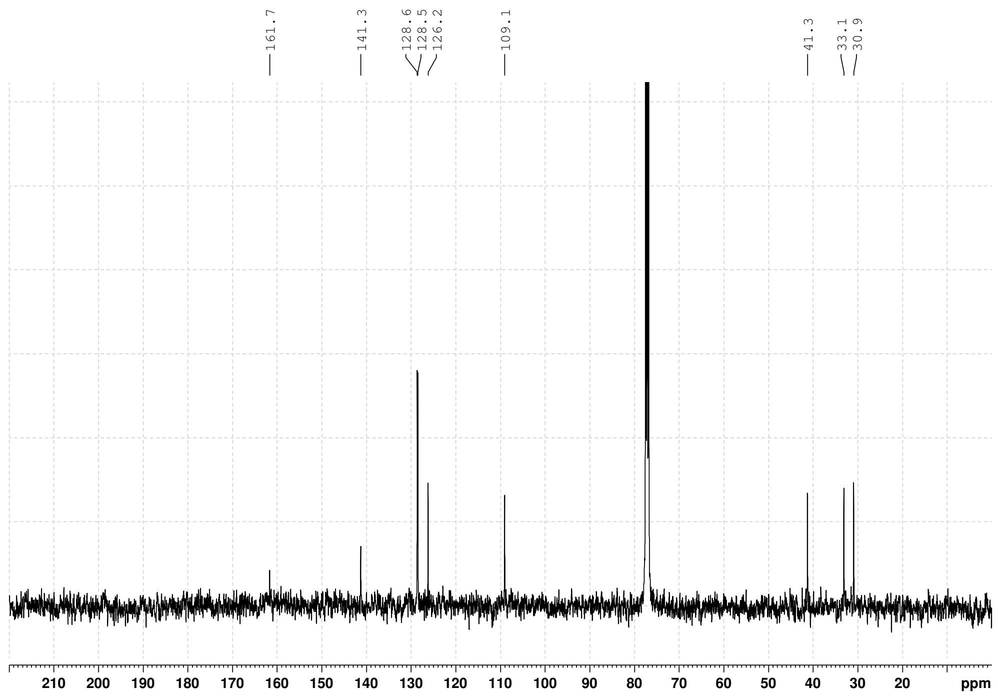

^{1}H-^{13}C HMBC

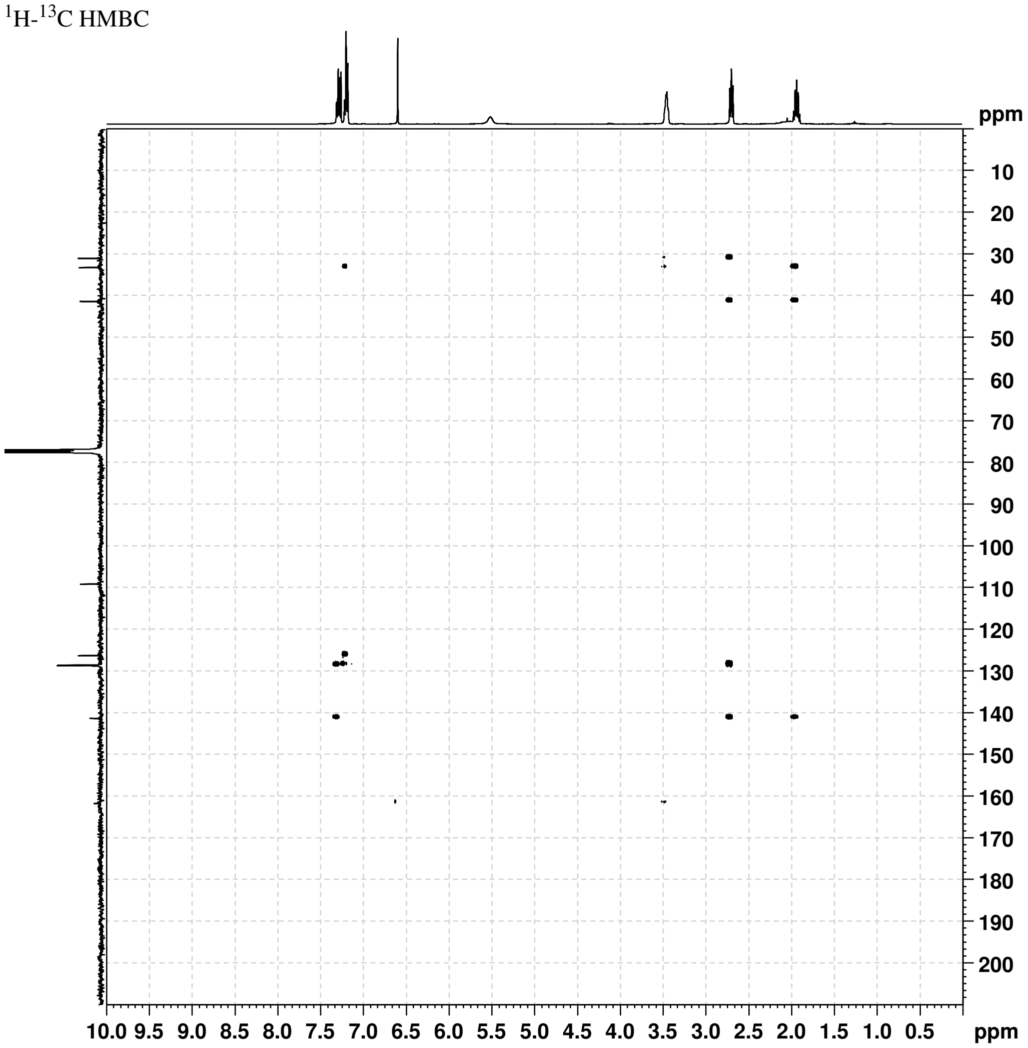
ppm
10
20
30
40
50
60
70
80
90
100
110
120
130
140
150
160
170
180
190
200
10.0 9.5 9.0 8.5 8.0 7.5 7.0 6.5 6.0 5.5 5.0 4.5 4.0 3.5 3.0 2.5 2.0 1.5 1.0 0.5
ppm

4,6-Dichloro-*N*-methyl-*N*-(3-phenylpropyl)pyrimidin-2-amine (17)

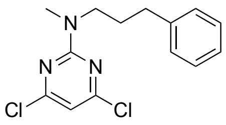

^{1}H NMR

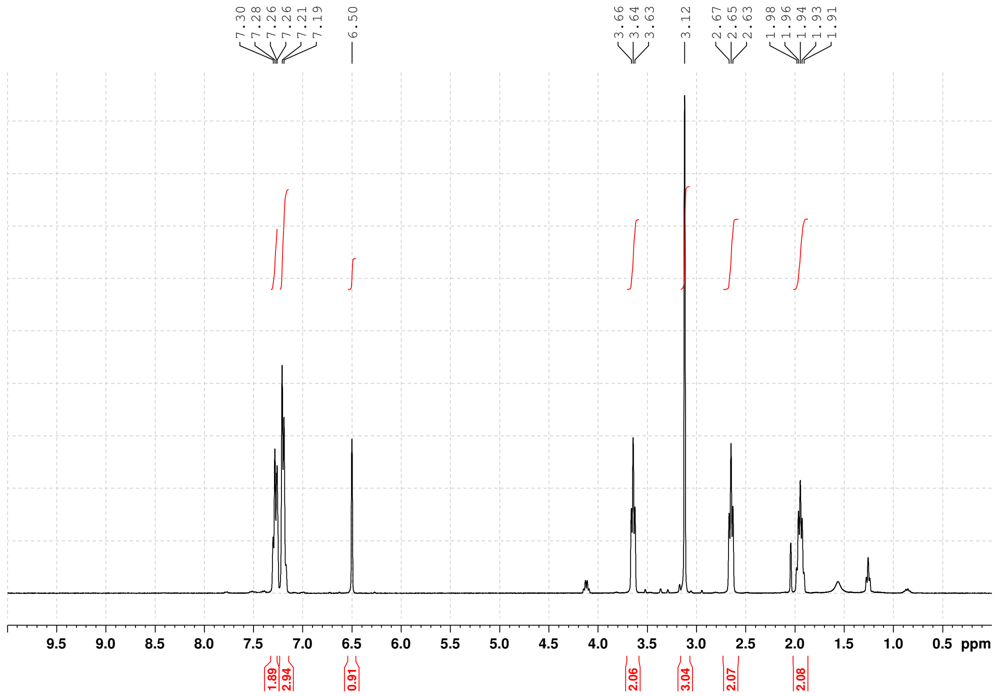

^{13}C NMR

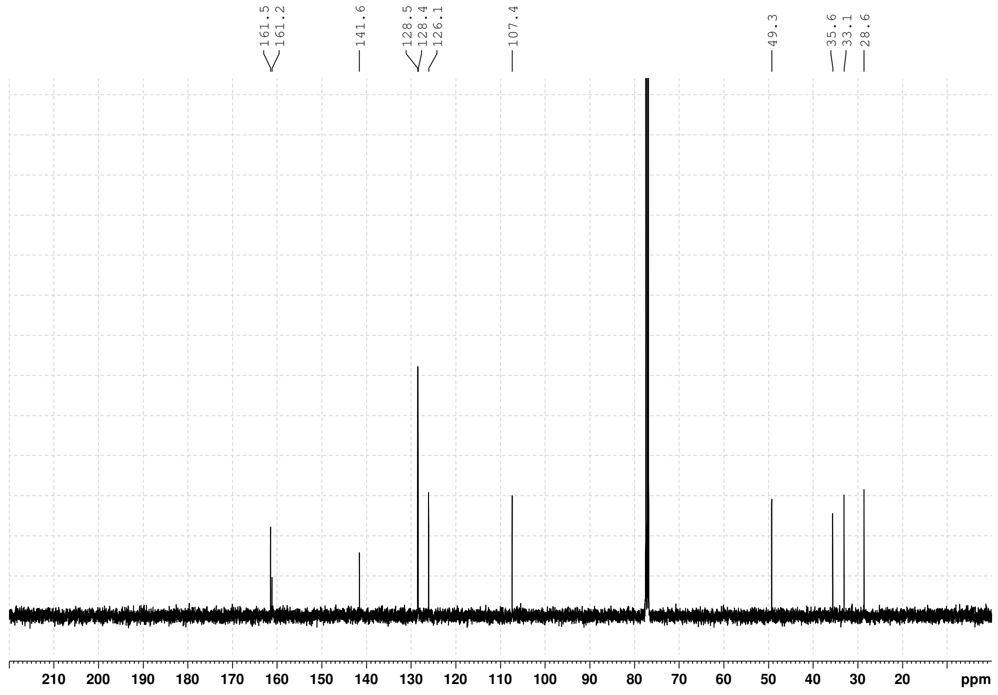

4,6-Dichloro-*N*-(3-phenylpropyl)-1,3,5-triazin-2-amine (18)

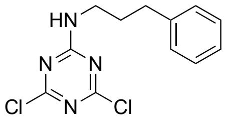

^{1}H NMR

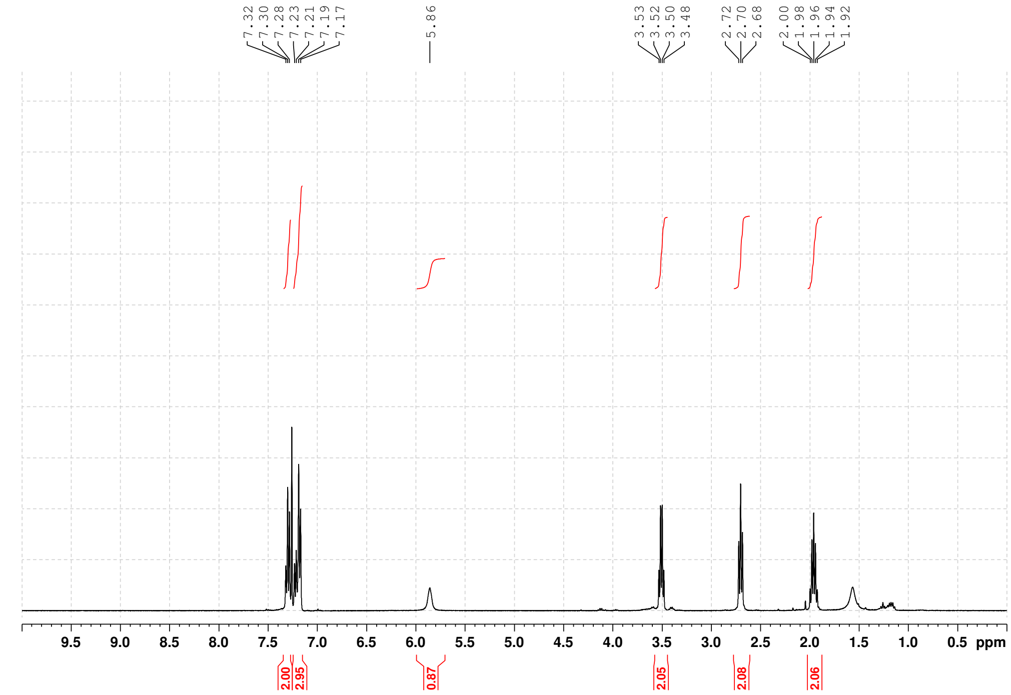

^{13}C NMR

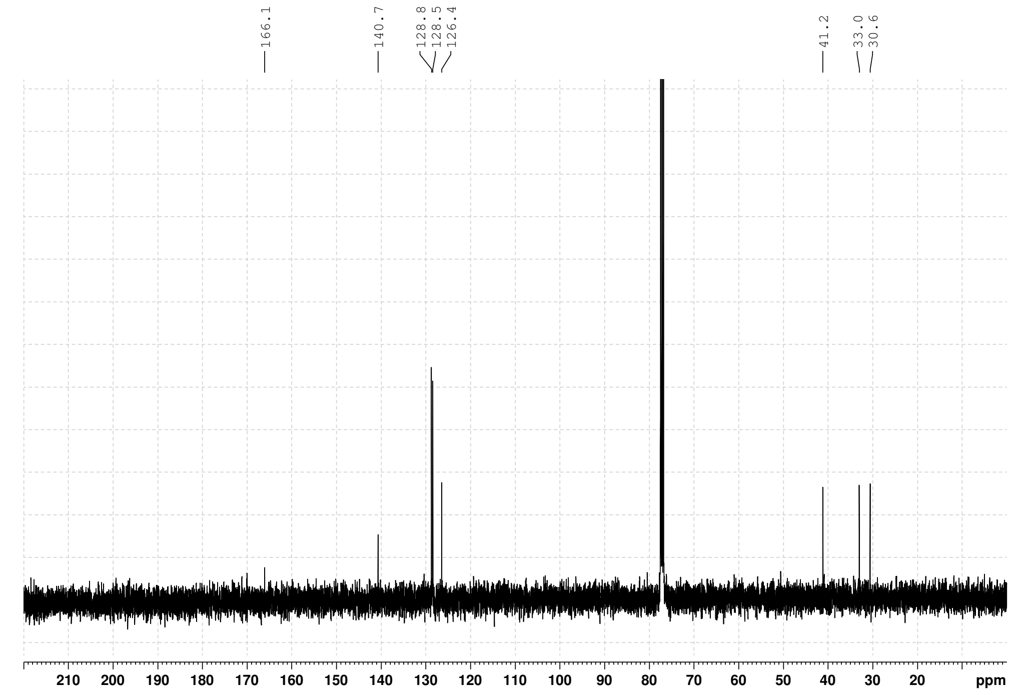

^{1}H-^{13}C HMBC

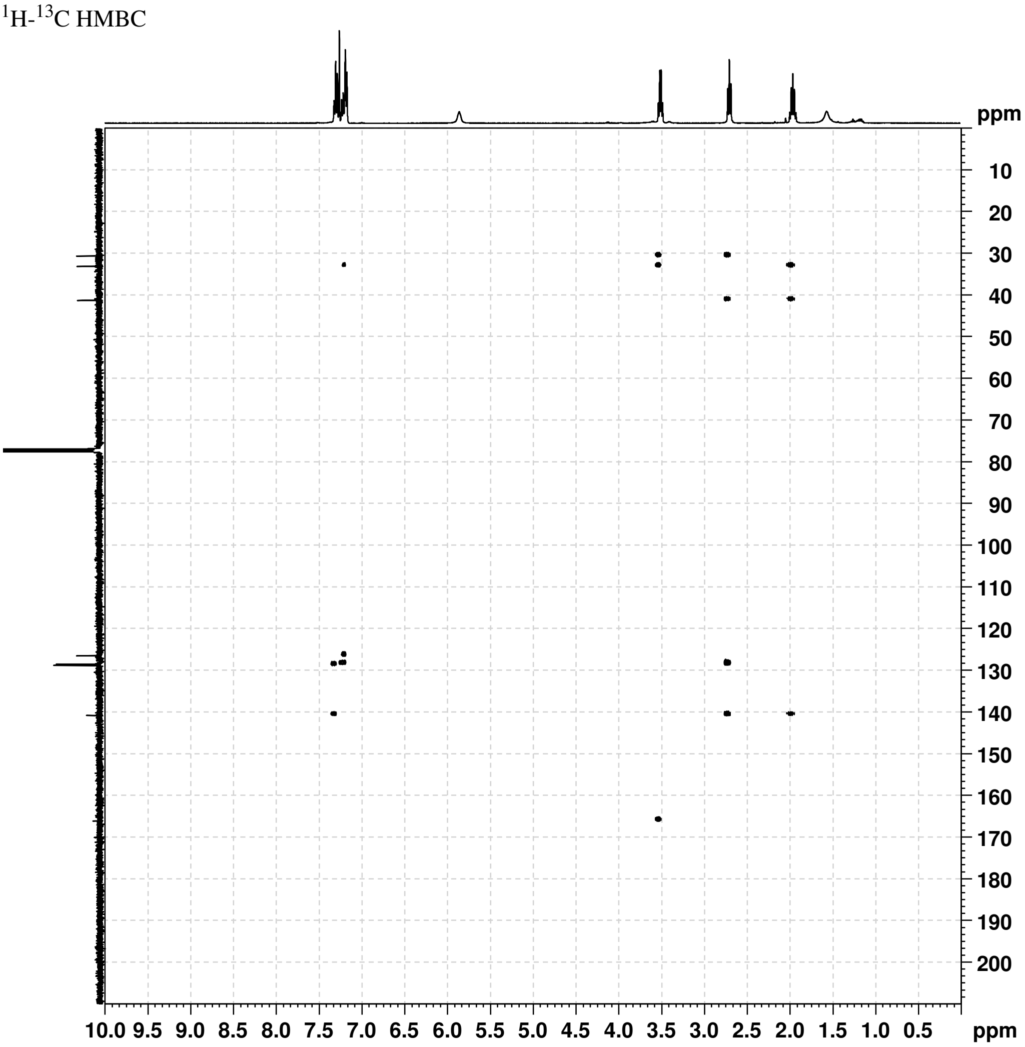
ppm
10
20
30
40
50
60
70
80
90
100
110
120
130
140
150
160
170
180
190
200
10.0 9.5 9.0 8.5 8.0 7.5 7.0 6.5 6.0 5.5 5.0 4.5 4.0 3.5 3.0 2.5 2.0 1.5 1.0 0.5
ppm

4,6-Dichloro-*N*-methyl-*N*-(3-phenylpropyl)-1,3,5-triazin-2-amine (19)

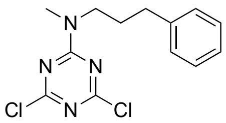

^{1}H NMR

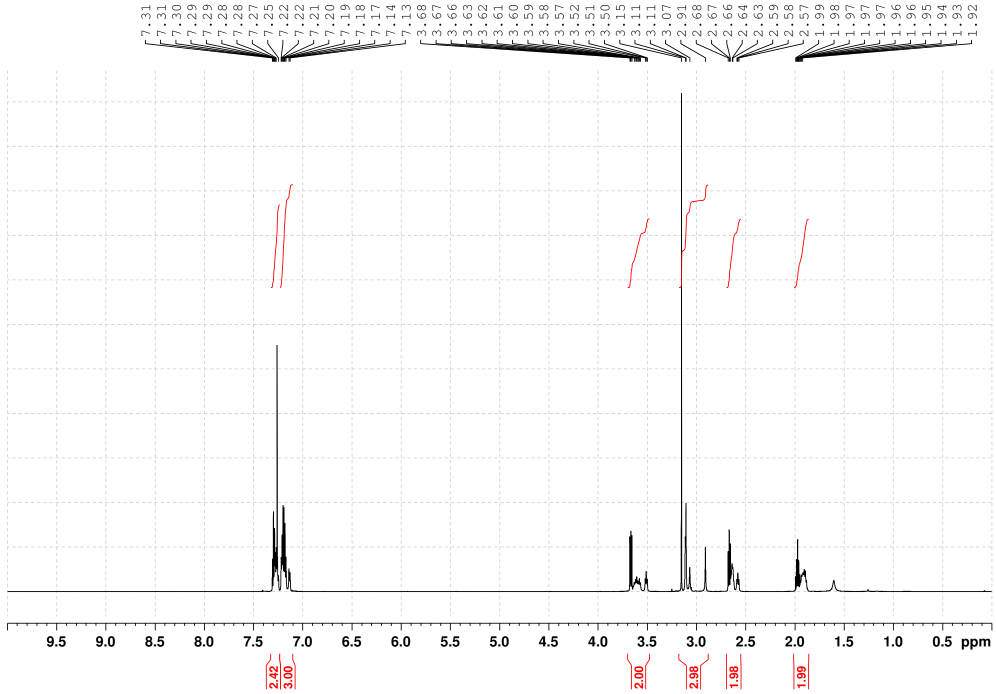

^{13}C NMR

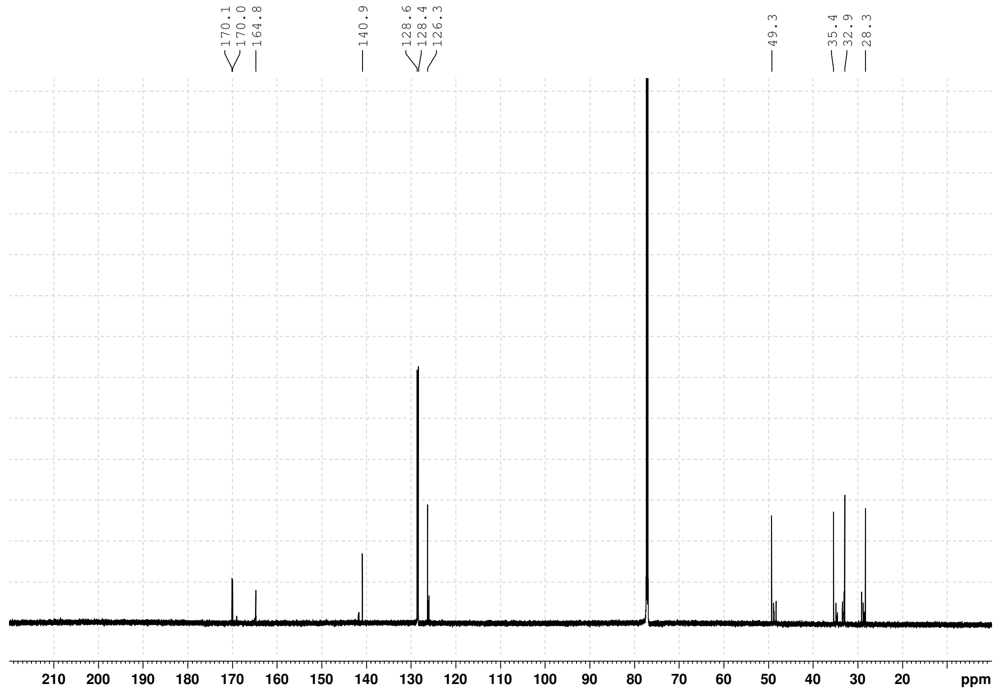

8 HPLC traces

Peptide P1, gradient 5–95 %B

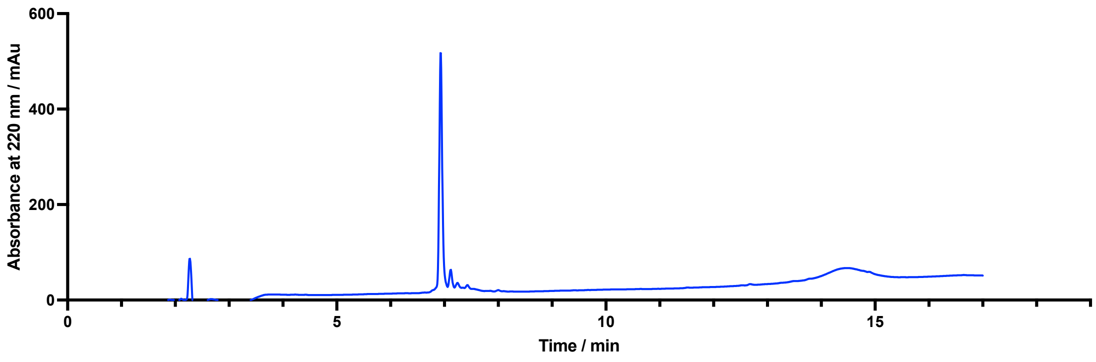

Peptide P2, gradient 5–95 %B

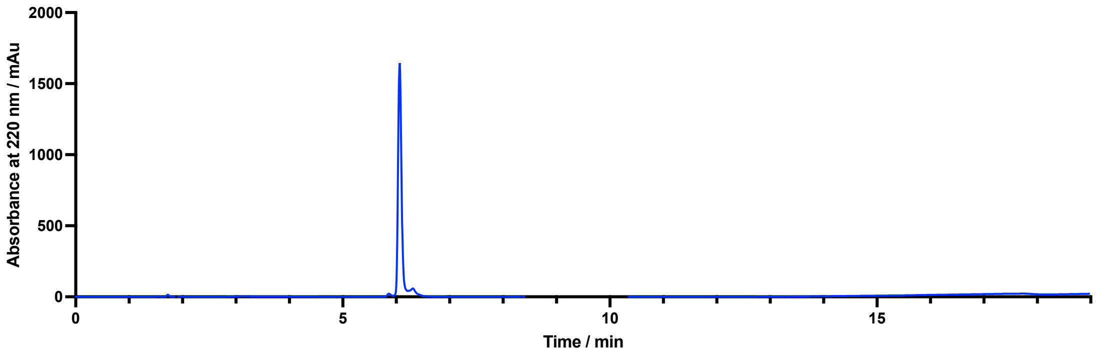

Peptide P2, gradient 20–60 %B

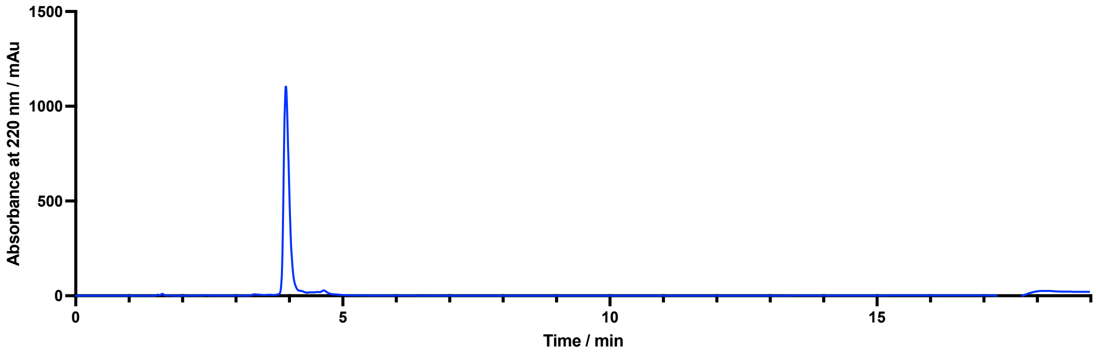

Peptide P2a, gradient 5–95 %B

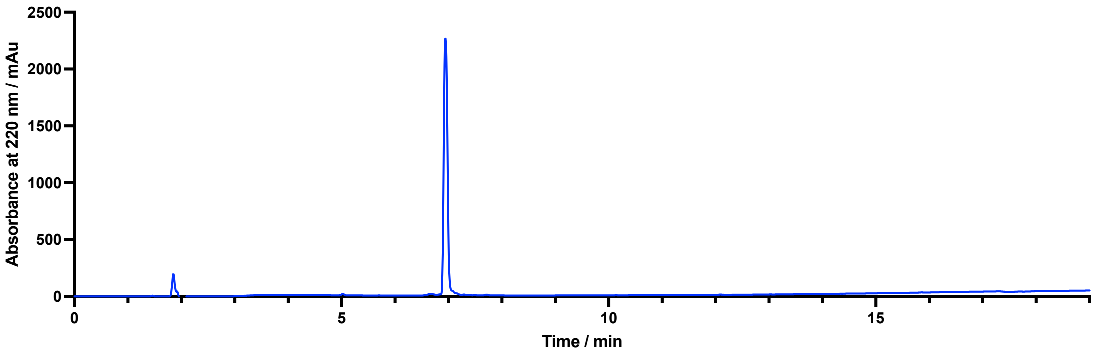

Peptide P2a, gradient 20–60 %B

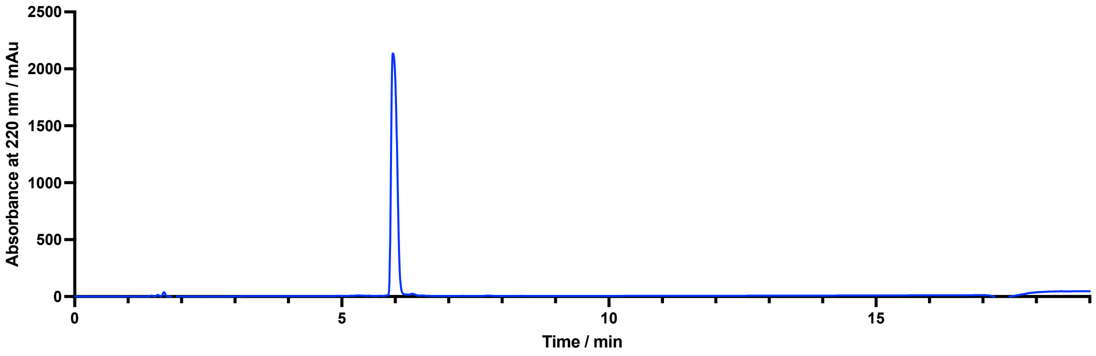

Peptide P3, gradient 5–95 %B

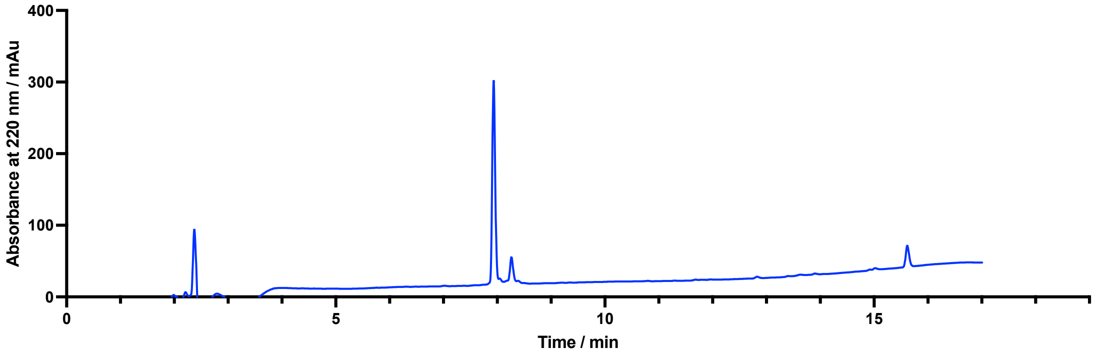

Peptide P4, gradient 5–95 %B

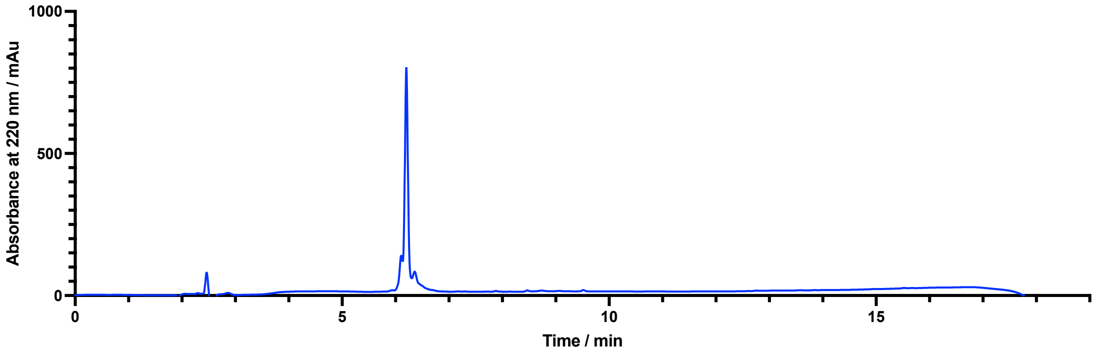

Peptide P4a, gradient 5–95 %B

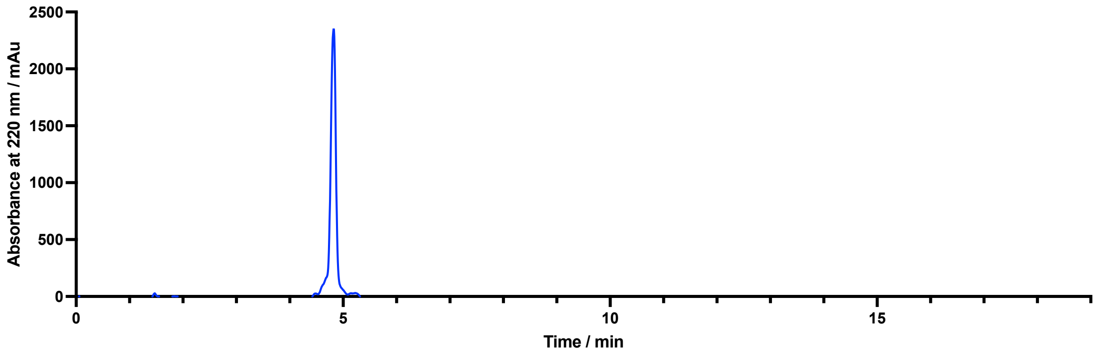

Peptide P4a, gradient 5–60 %B

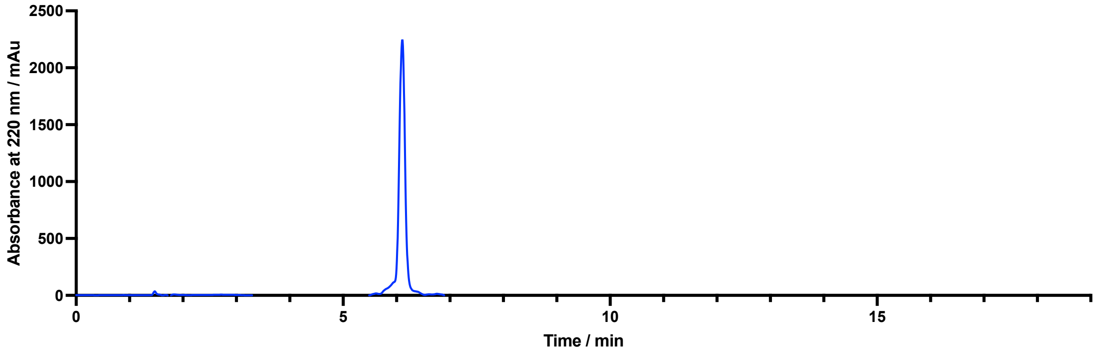

Peptide P5, gradient 5–95 %B

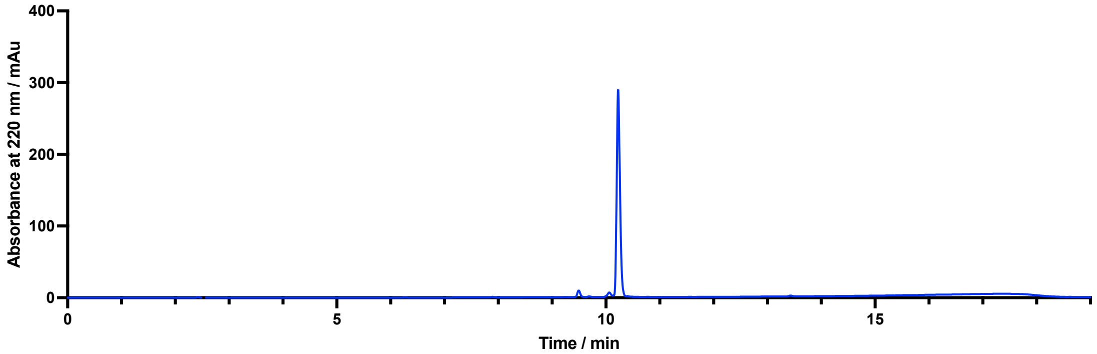

Peptide P6, gradient 5–95 %B

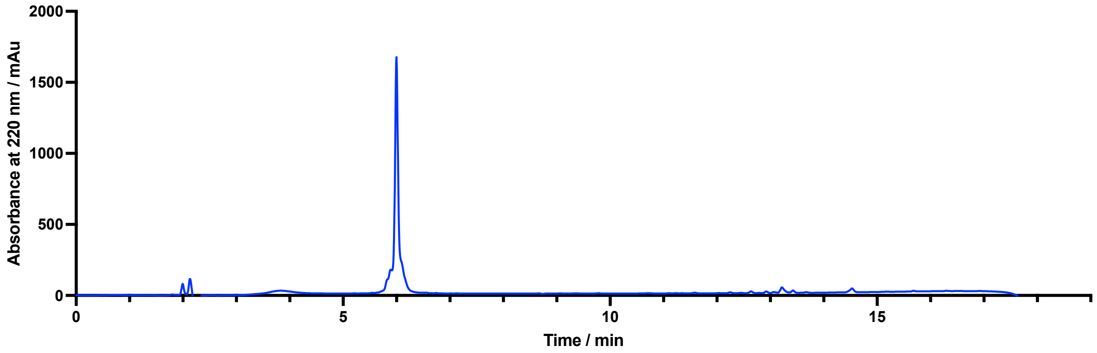

Peptide P6a, gradient 5–95 %B

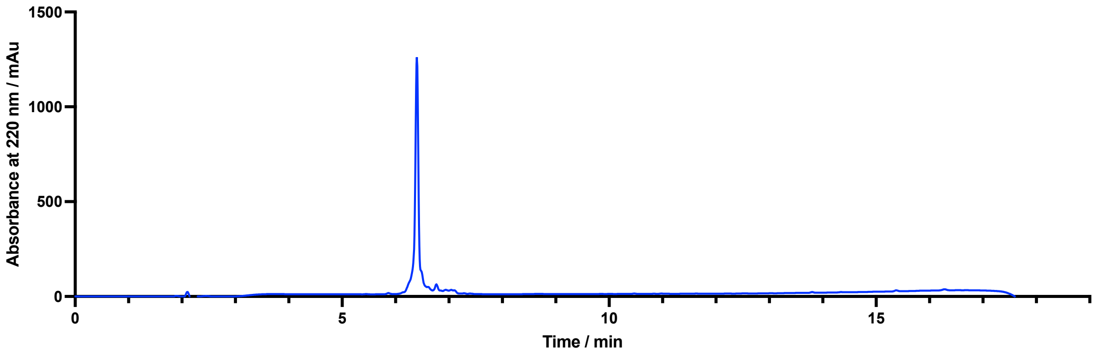

Peptide P6a, gradient 5–60 %B

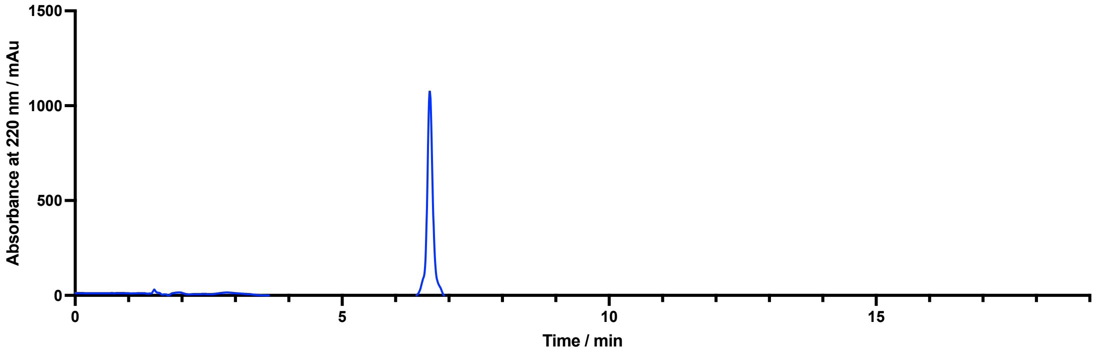

Peptide P7, gradient 5–95 %B

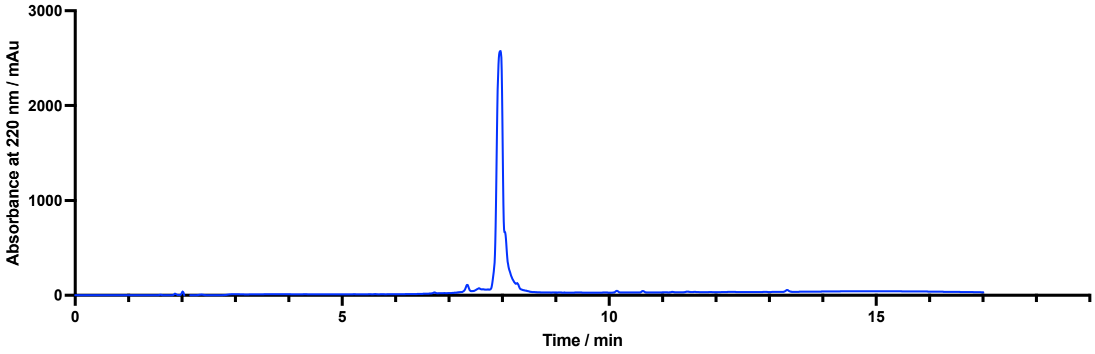

Peptide P8, gradient 5–95 %B

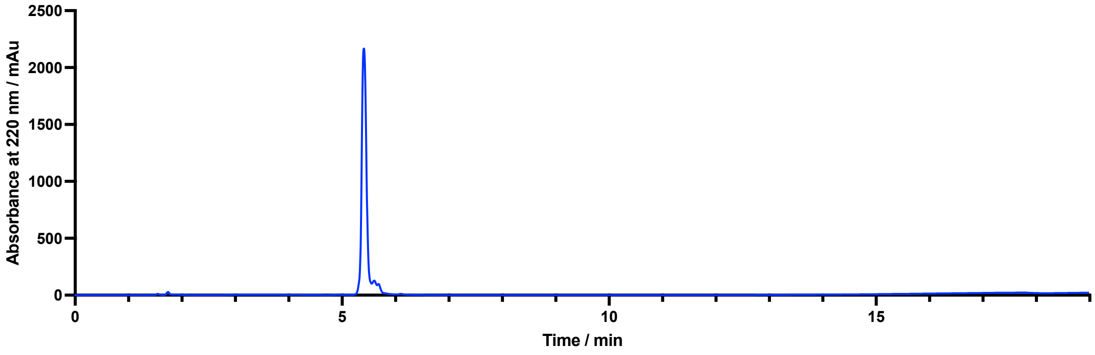

Peptide P8, gradient 10–60 %B

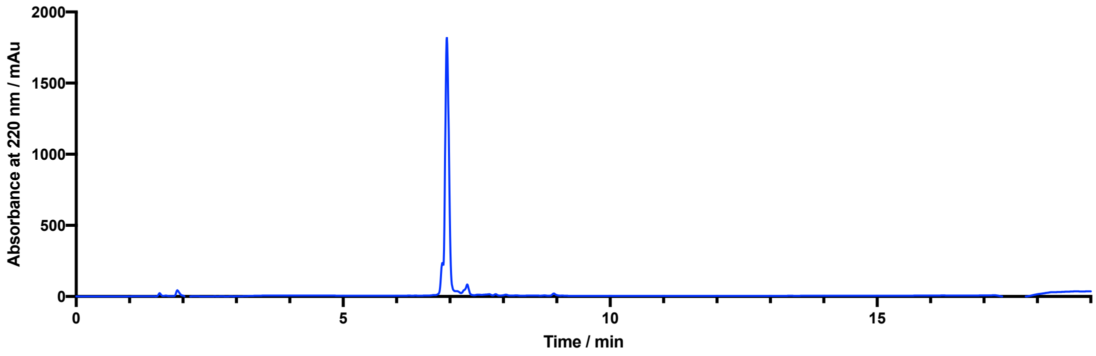

Peptide P9, gradient 5–95 %B

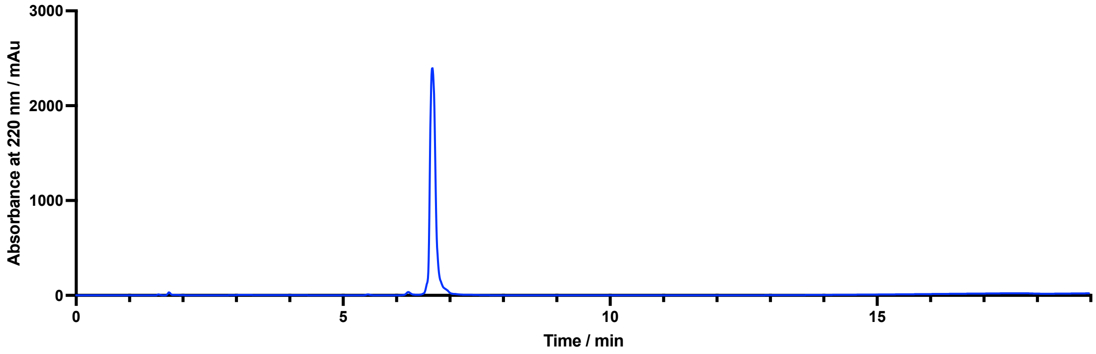

Peptide P9, gradient 10–60 %B

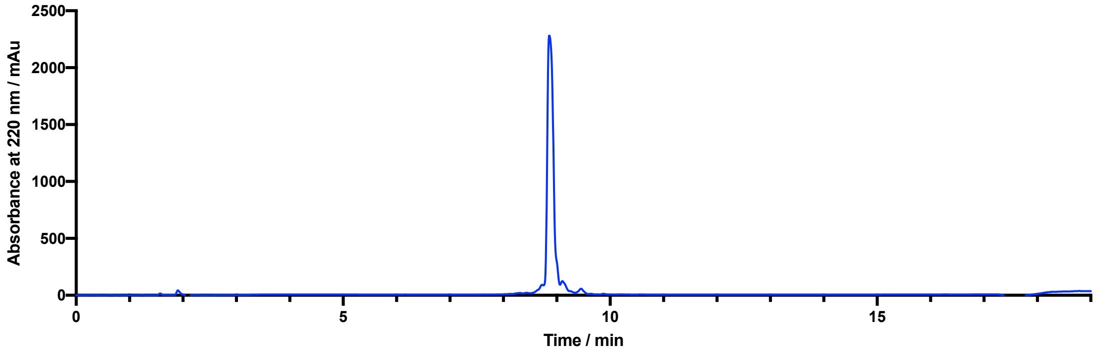

Peptide P10, gradient 5–95 %B

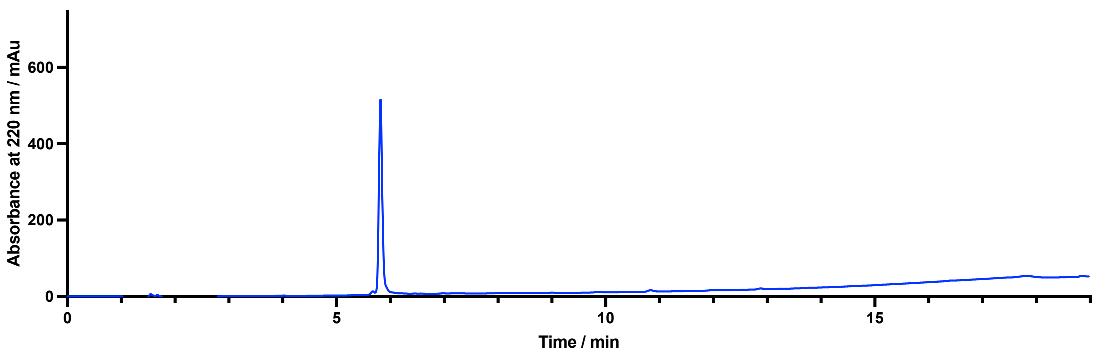

Peptide P10, gradient 10–60 %B

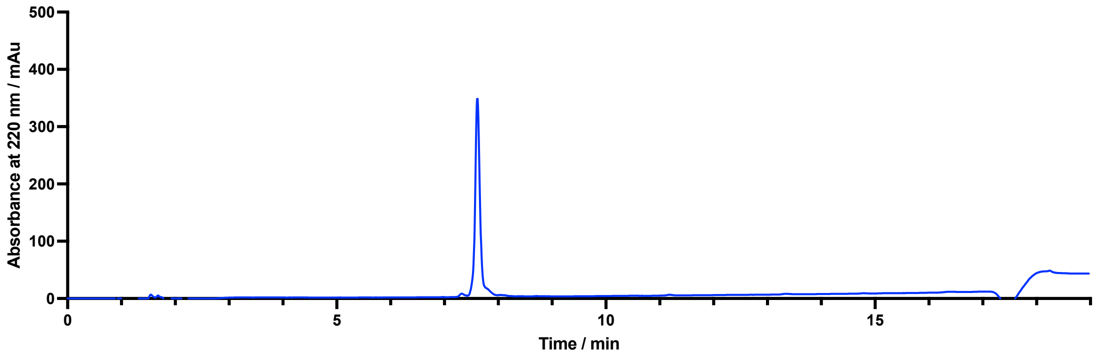

Peptide P11, gradient 5–95 %B

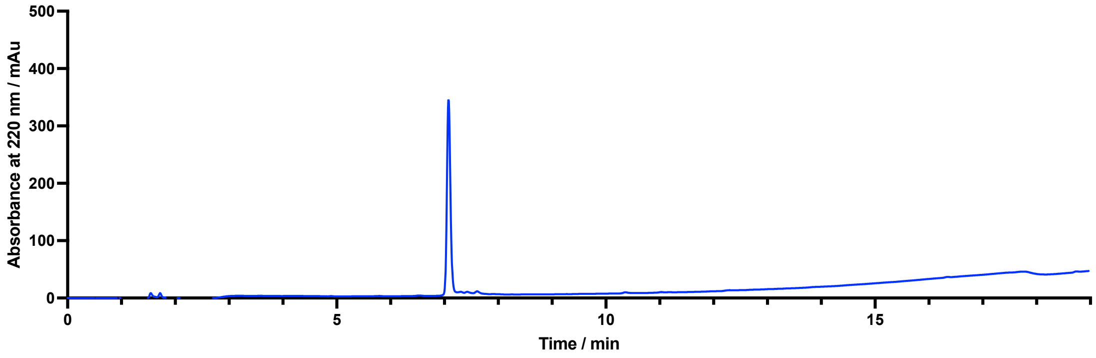

Peptide P11, gradient 10–60 %B

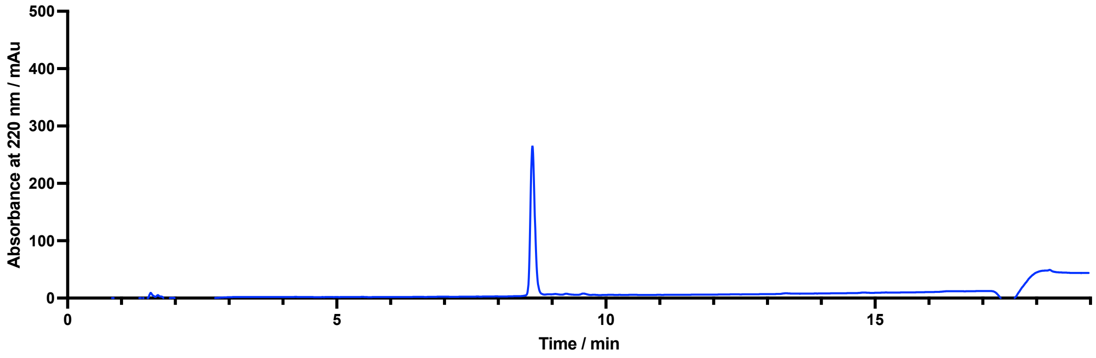

Peptide P12, gradient 5–95 %B

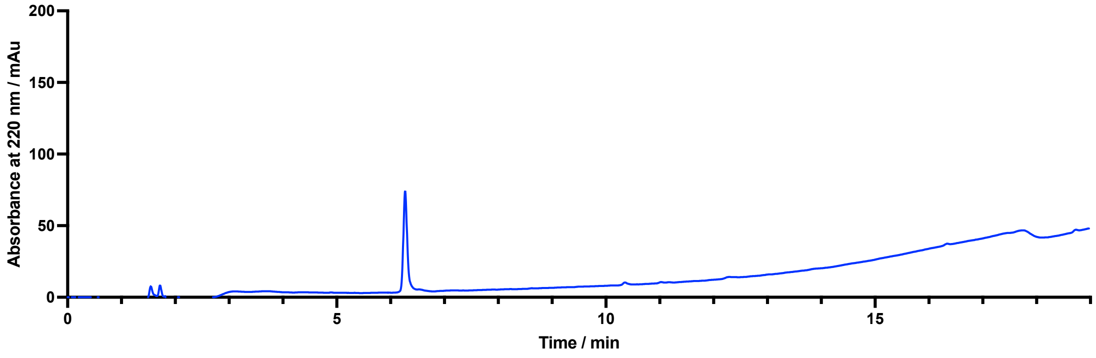

Peptide P12, gradient 10–60 %B

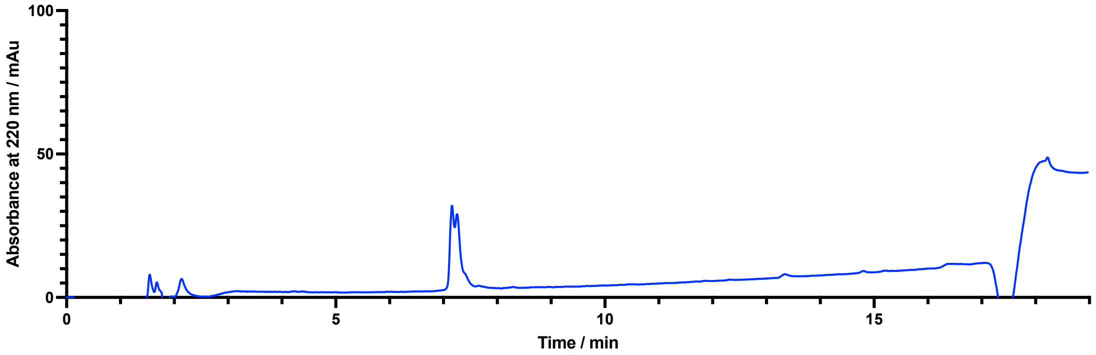

Stapled peptide P1-1, gradient 5–95 %B

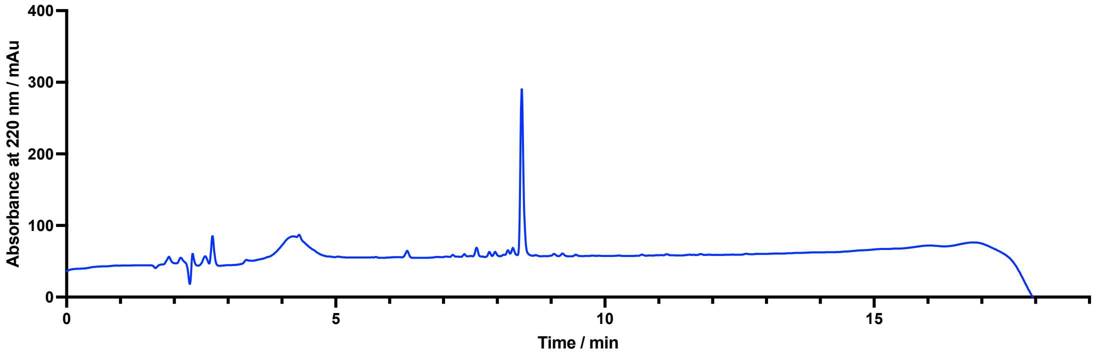

Stapled peptide P1-1, gradient 10–60 %B

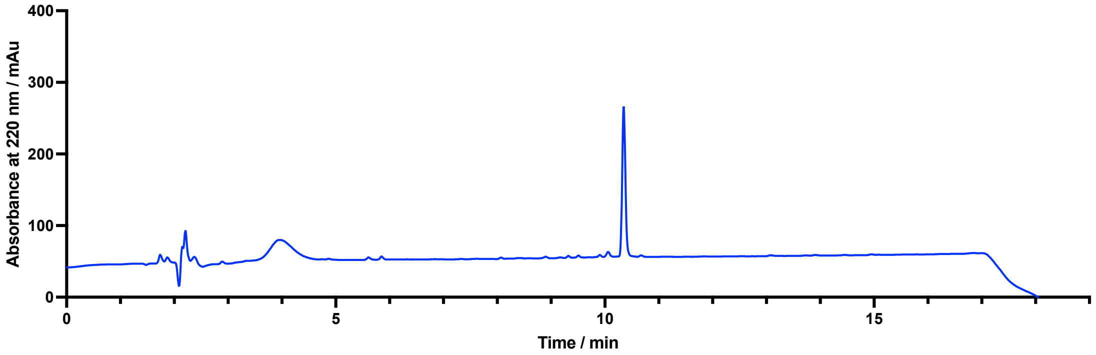

Stapled peptide P2-10, gradient 5–95 %B

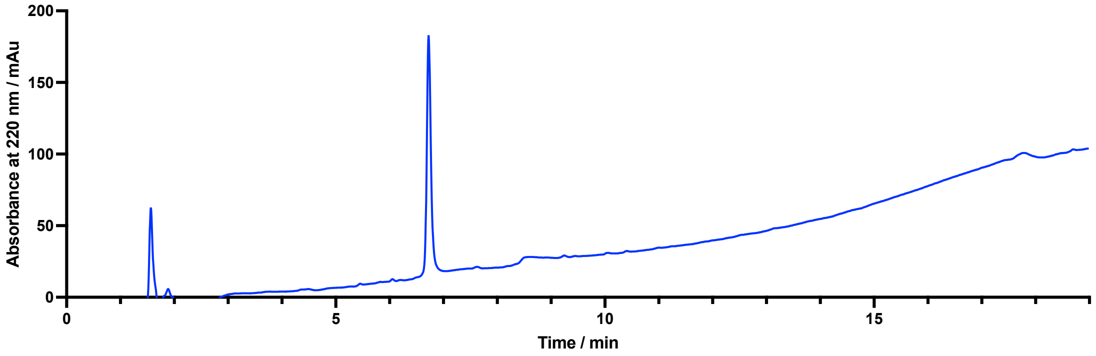

Stapled peptide P2-10, gradient 20–60 %B

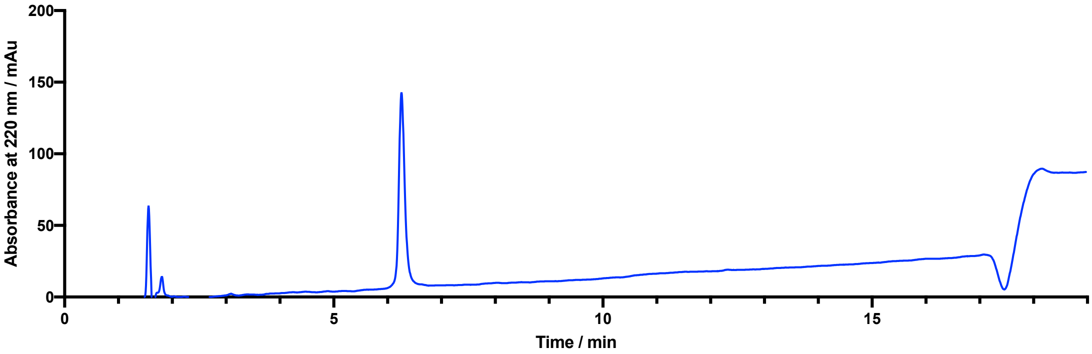

Stapled peptide P2-8, gradient 5–95 %B

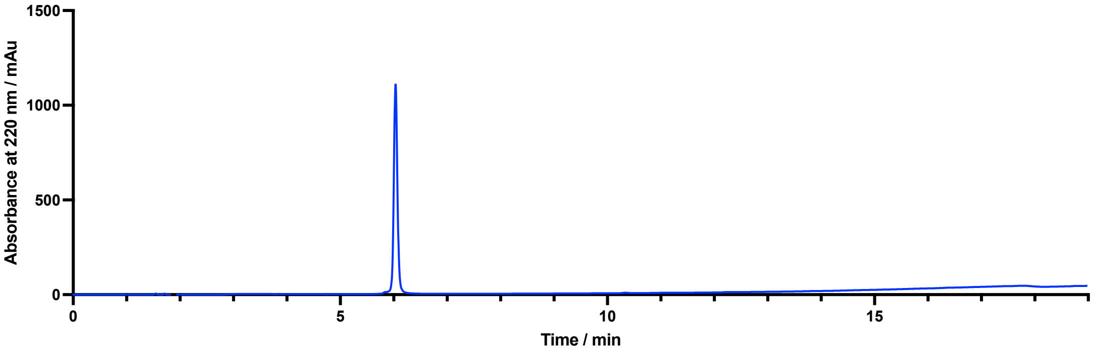

Stapled peptide P2-8, gradient 10–60 %B

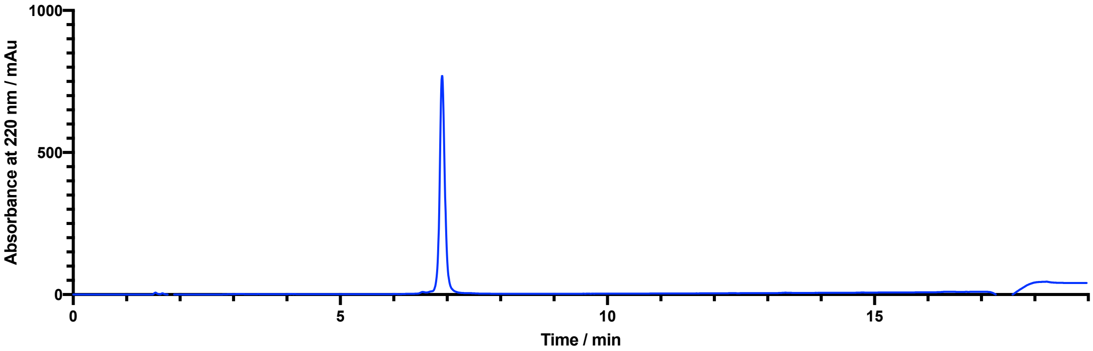

Stapled peptide P2-1, gradient 5–95 %B

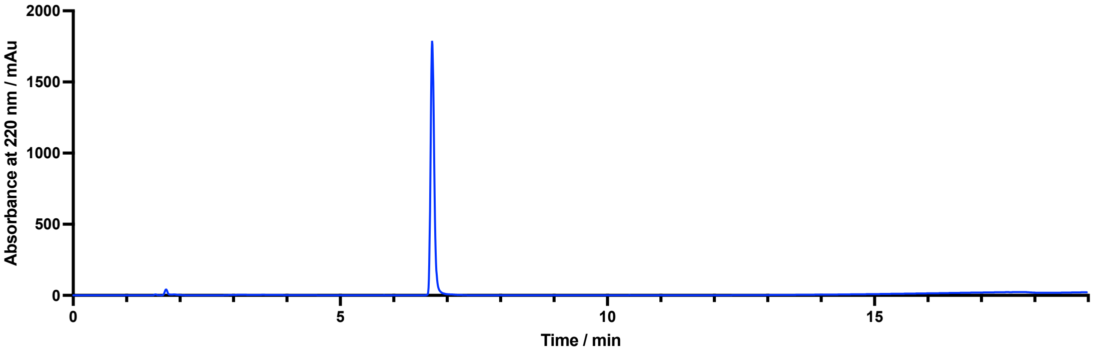

Stapled peptide P2-2, gradient 5–95 %B

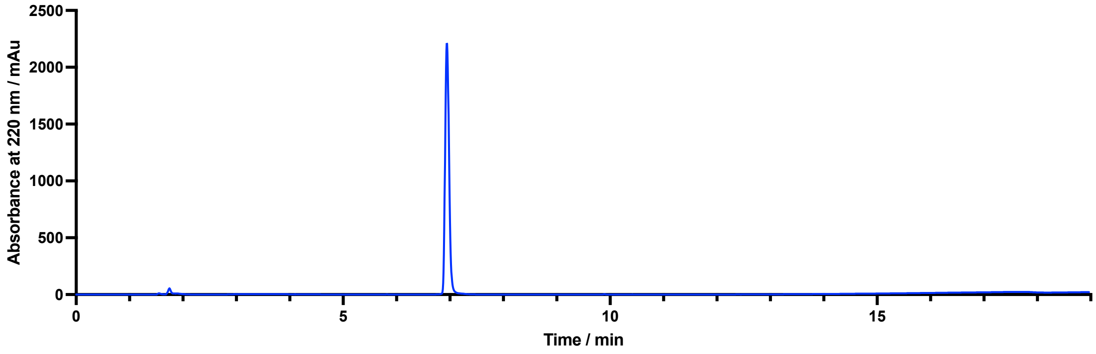

Stapled peptide P2-3, gradient 5–95 %B

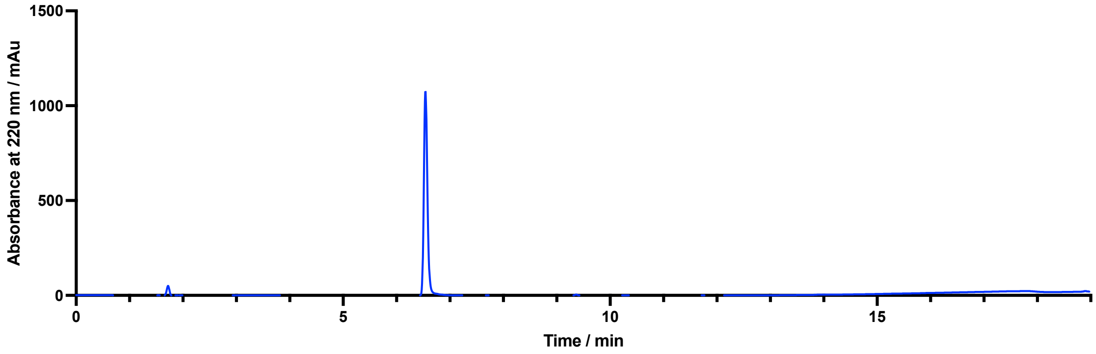

Stapled peptide P2-4, gradient 5–95 %B

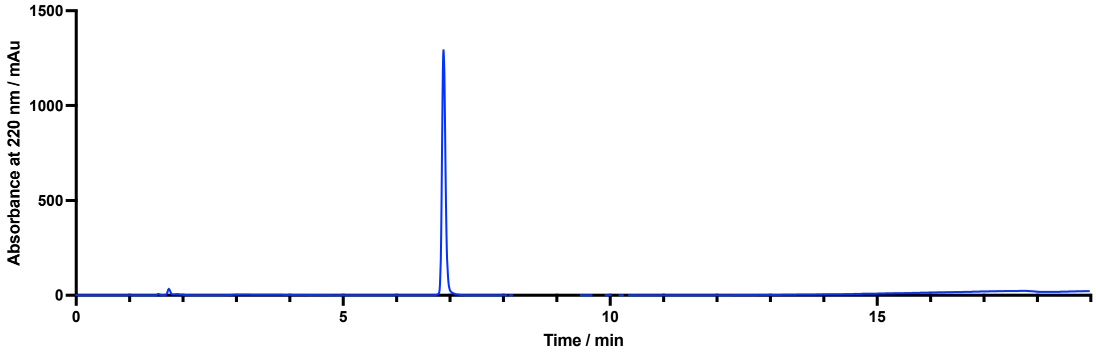

Stapled peptide P3-1, gradient 5–95 %B

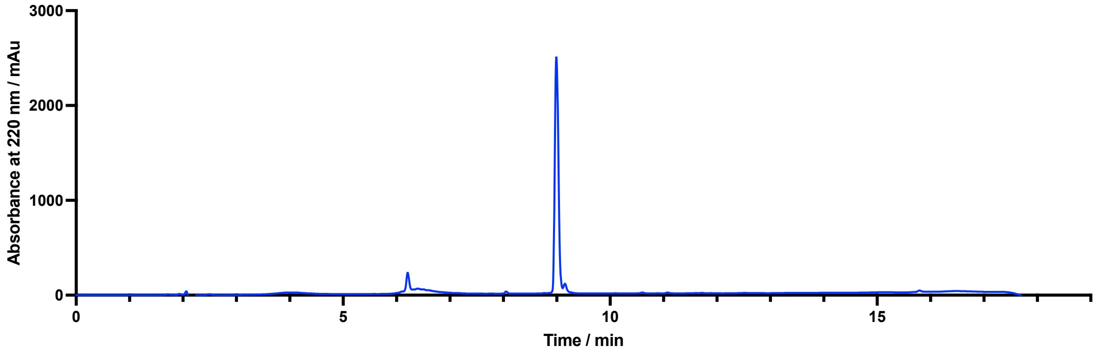

Stapled peptide P4-1, gradient 5–95 %B

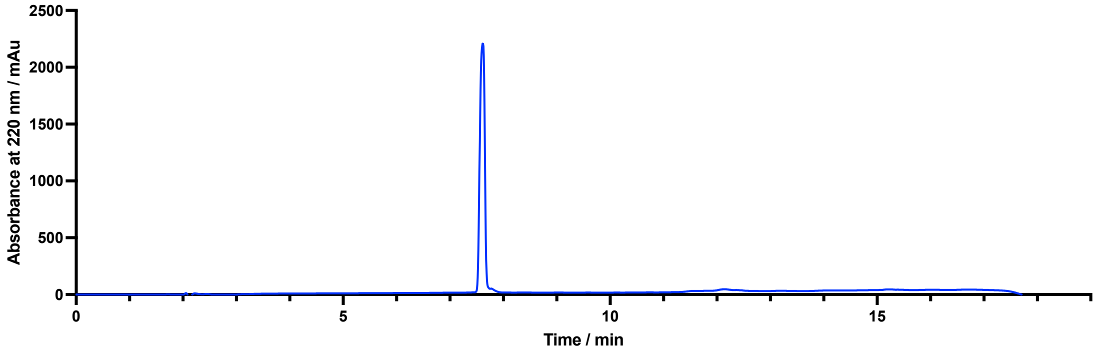

Stapled peptide P5-1, gradient 5–95 %B

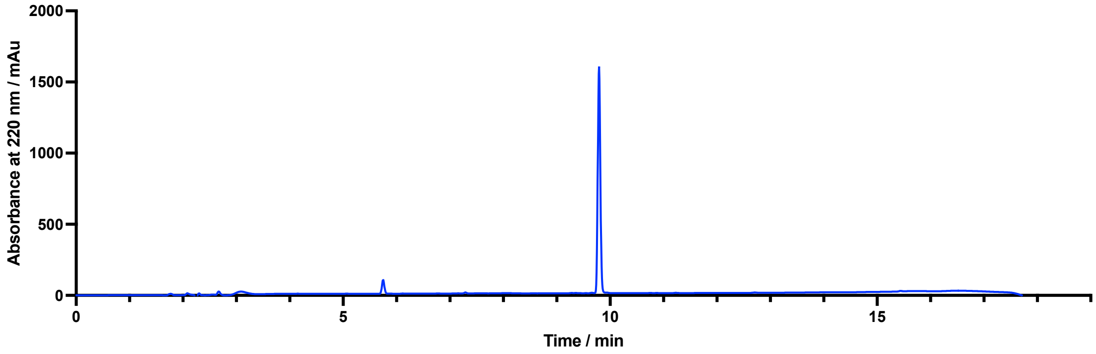

Stapled peptide P5-1, gradient 10–60 %B

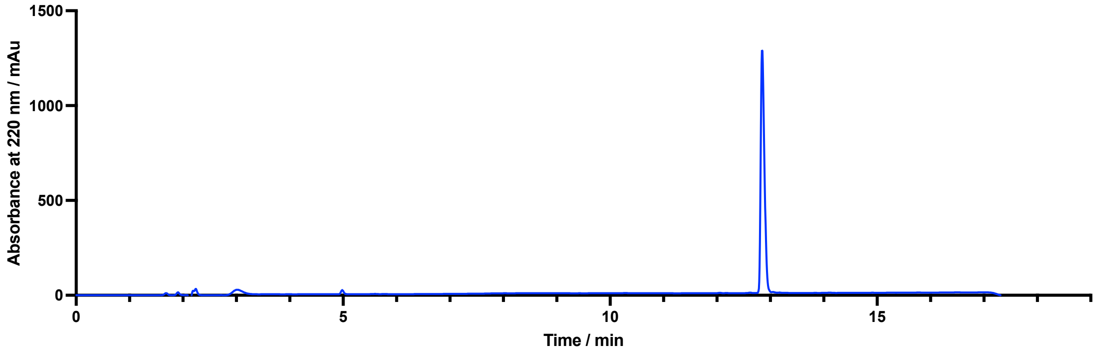

Stapled peptide P6-1, gradient 5–95 %B

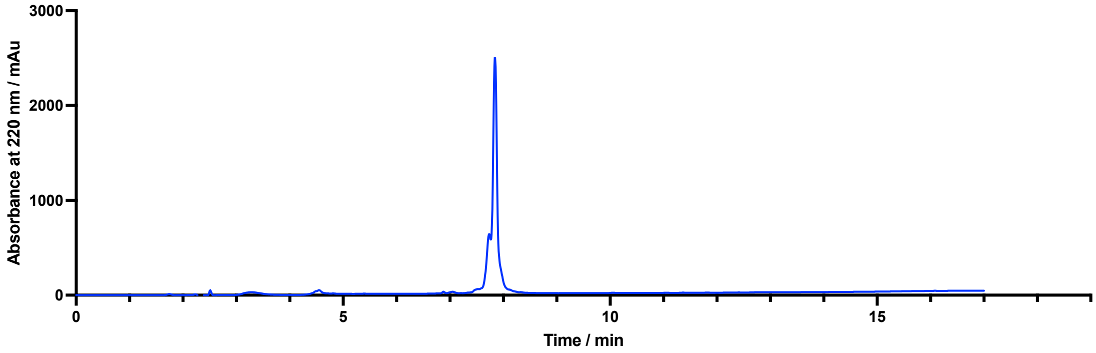

Stapled peptide P6-1, gradient 10–80 %B

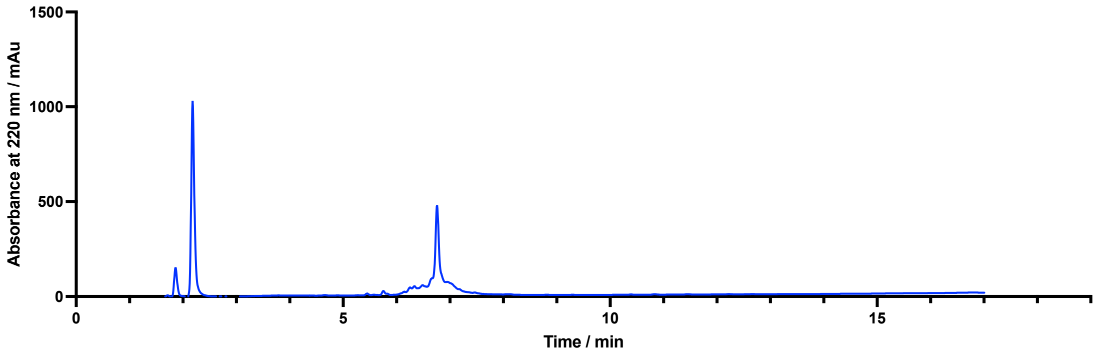

Stapled peptide P7-1, gradient 5–95 %B

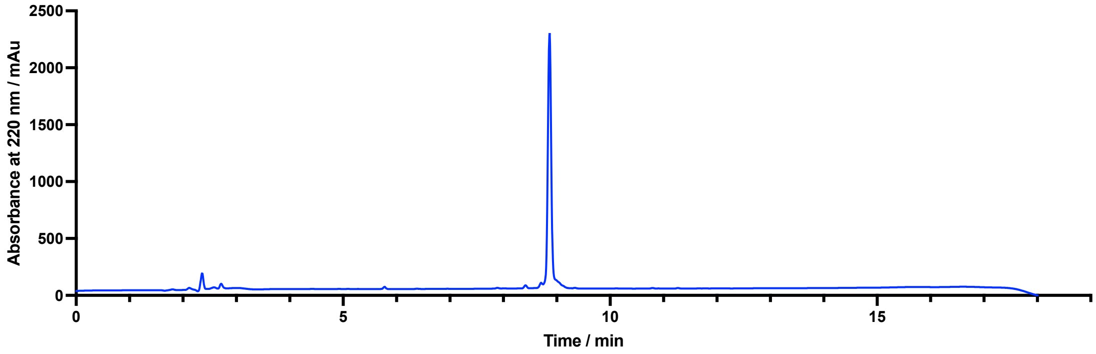

Stapled peptide P7-1, gradient 10–60 %B

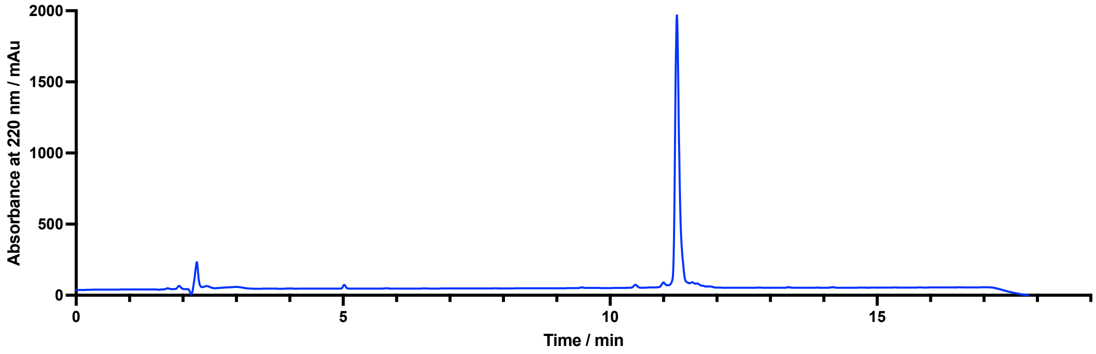

Stapled peptide P8-8, gradient 5–95 %B

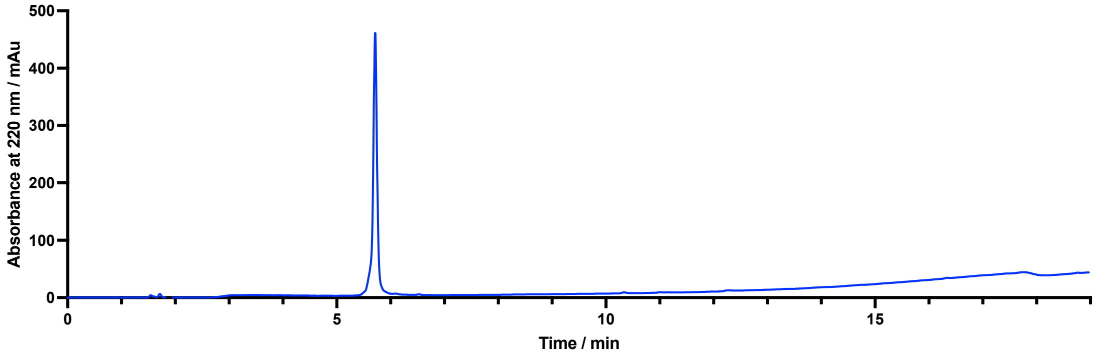

Stapled peptide P8-8, gradient 10–60 %B

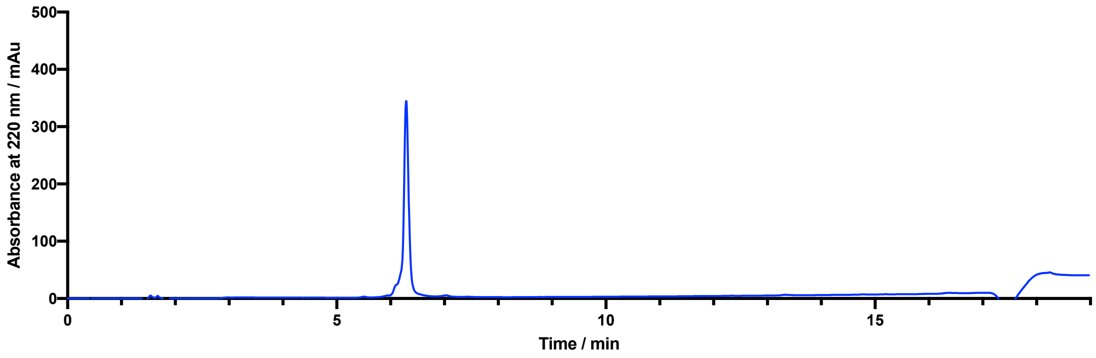

Stapled peptide P8-1, gradient 5–95 %B

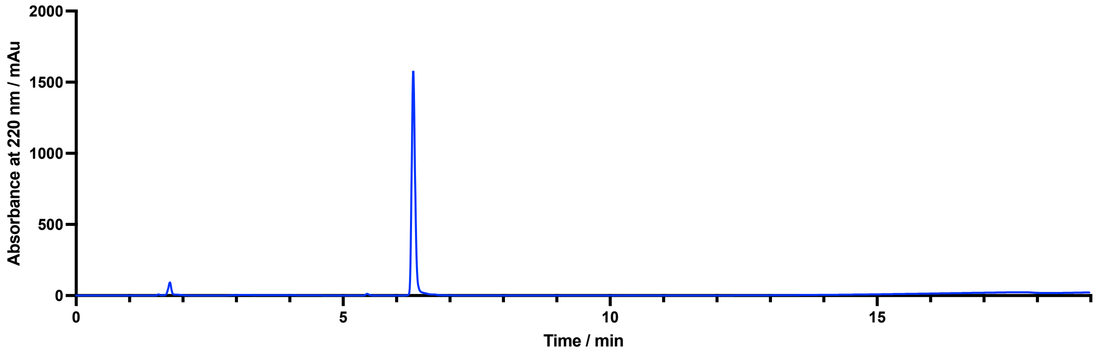

Stapled peptide P8-2, gradient 5–95 %B

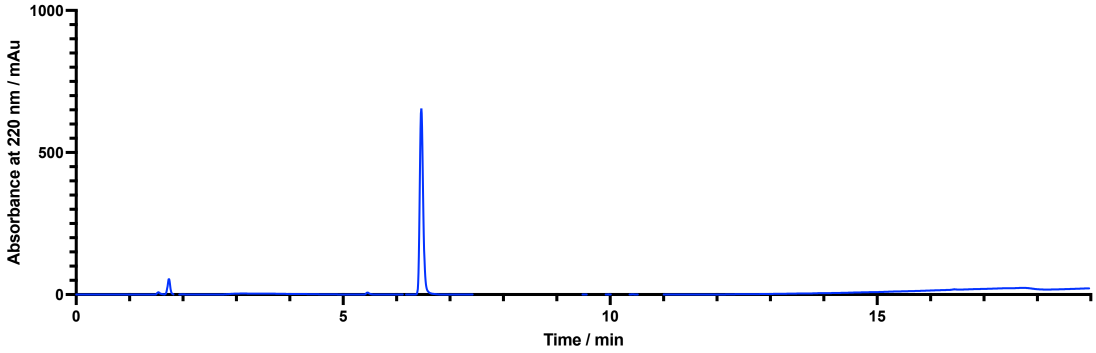

Stapled peptide P8-3, gradient 5–95 %B

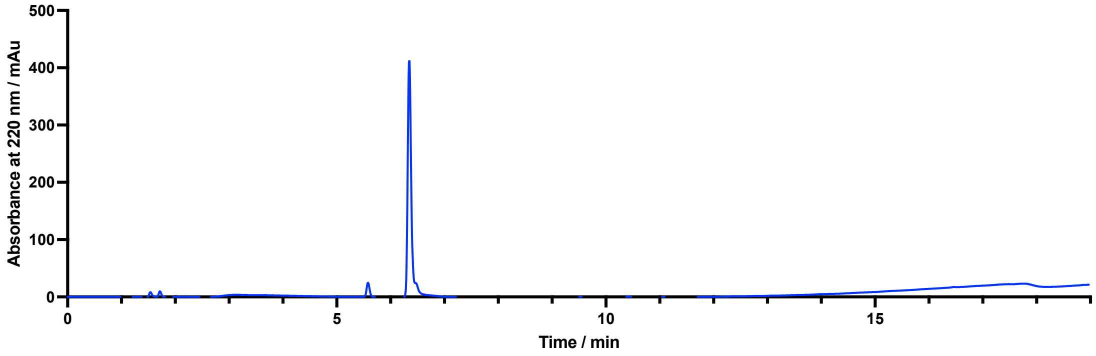

Stapled peptide P8-4, gradient 5–95 %B

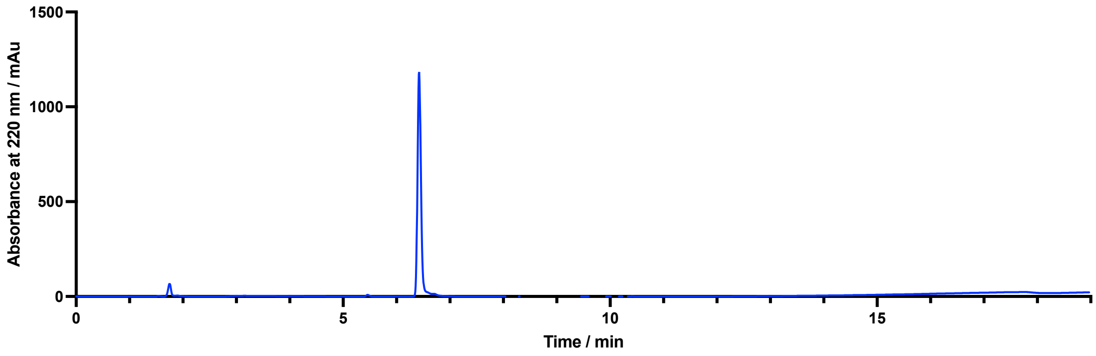

Stapled peptide P9-8, gradient 5–95 %B

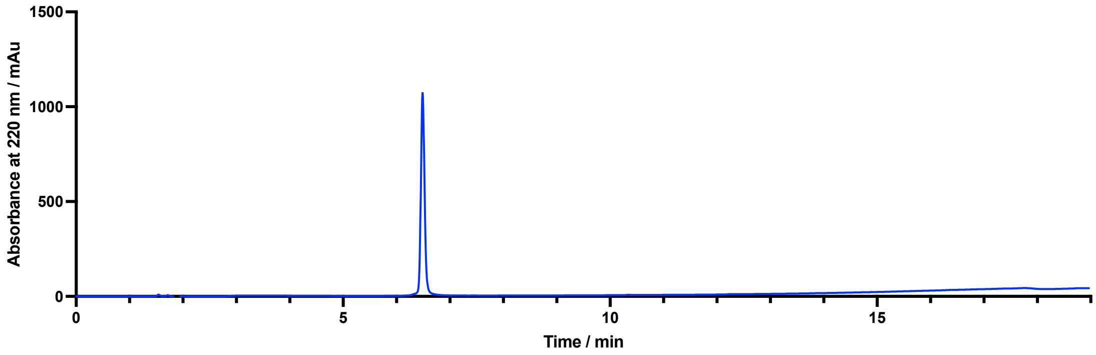

Stapled peptide P9-8, gradient 10–60 %B

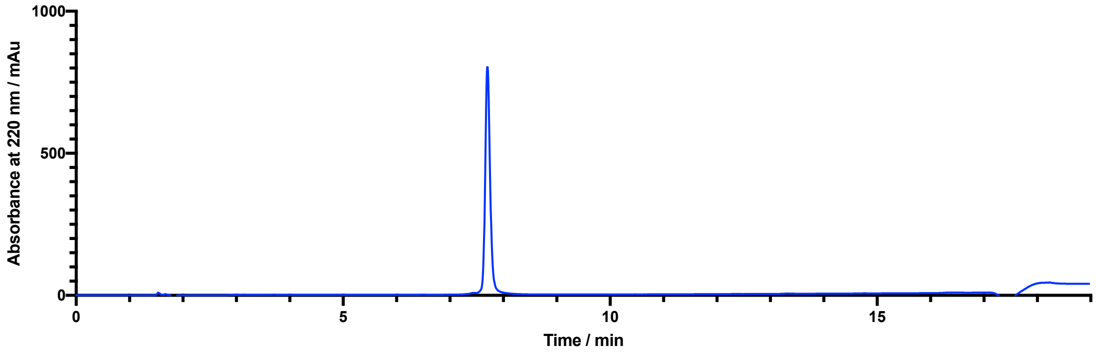

Stapled peptide P9-1, gradient 5–95 %B

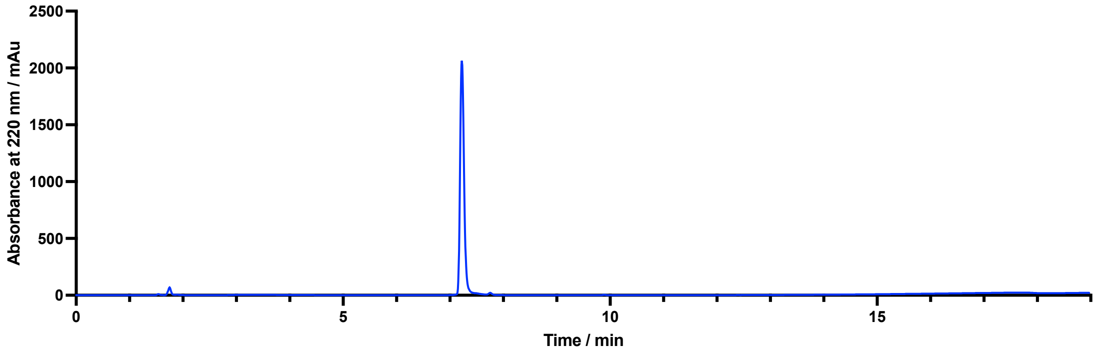

Stapled peptide P9-2, gradient 5–95 %B

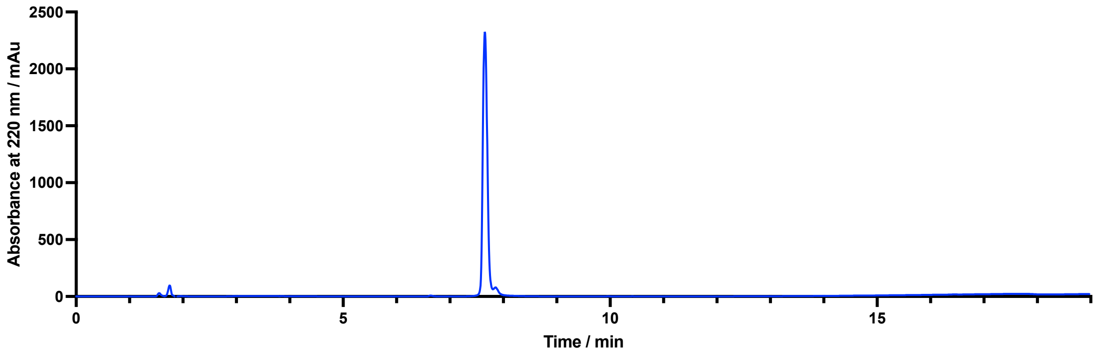

Stapled peptide P9-3, gradient 5–95 %B

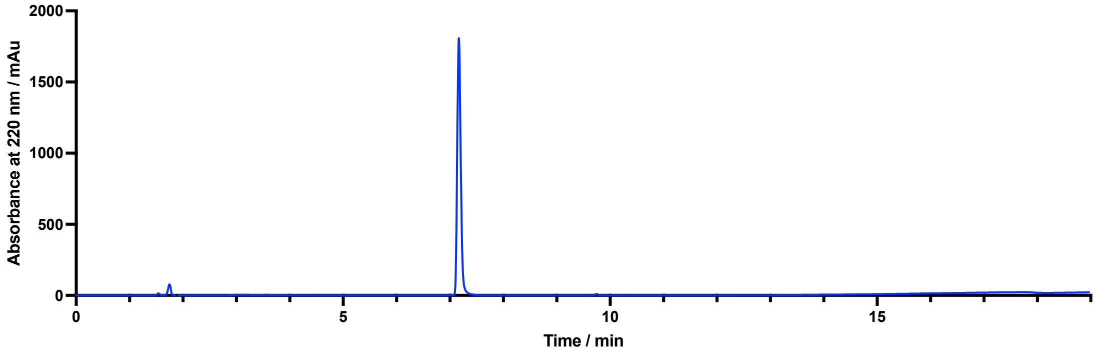

Stapled peptide P9-4, gradient 5–95 %B

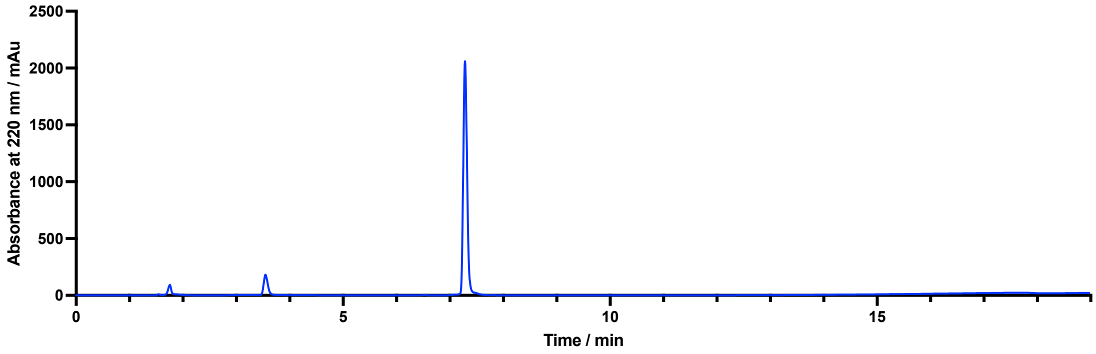

Stapled peptide P10-8, gradient 5–95 %B

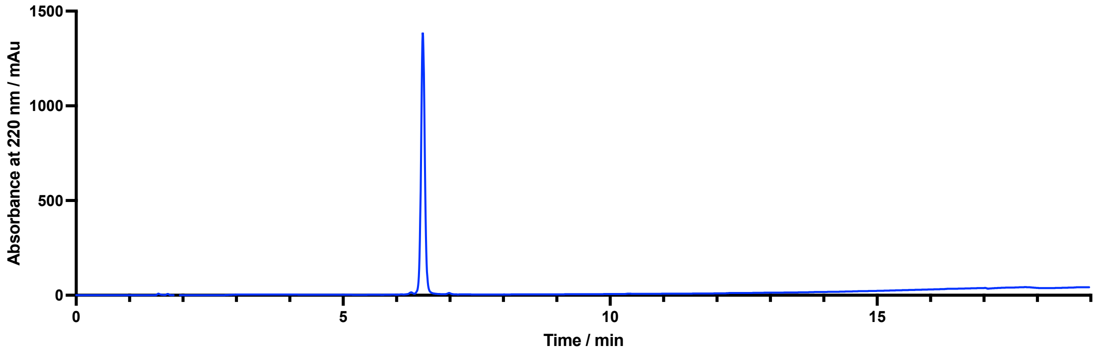

Stapled peptide P10-8, gradient 10–60 %B

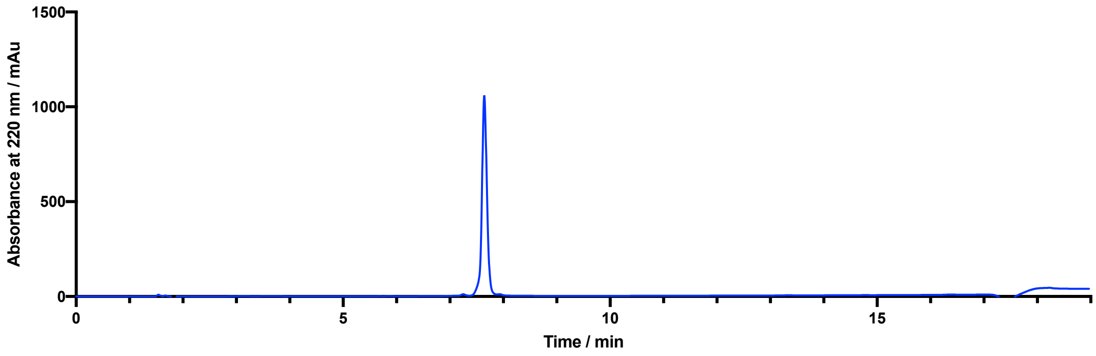

Stapled peptide P11-8, gradient 5–95 %B

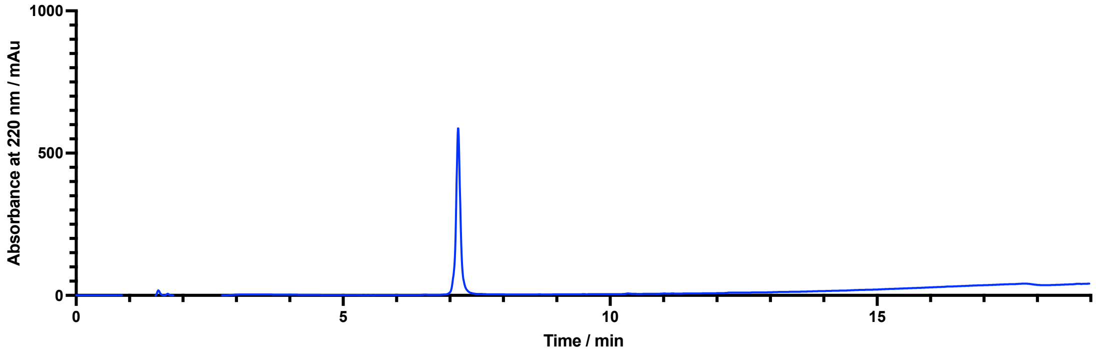

Stapled peptide P11-8, gradient 10–60 %B

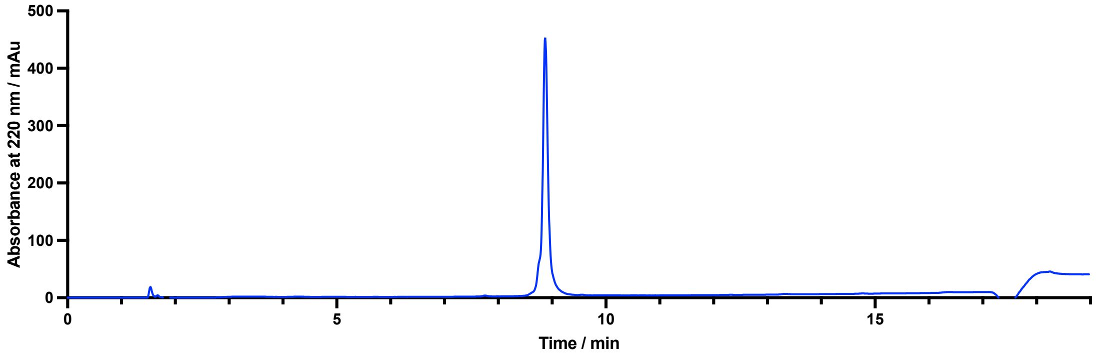

Stapled peptide P12-8, gradient 5–95 %B

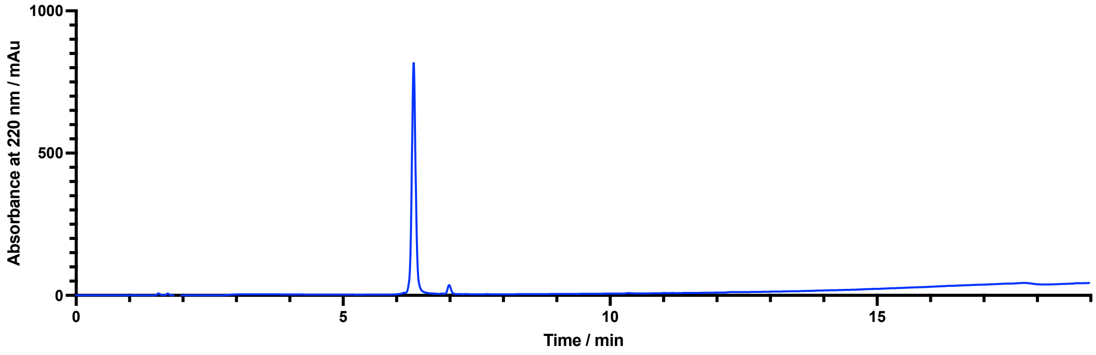

Stapled peptide P12-8, gradient 10–60 %B

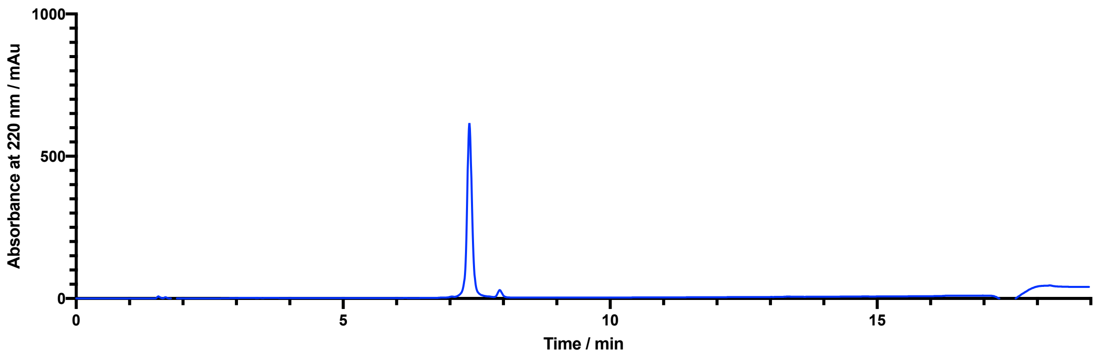

Stapled peptide P2-20, gradient 5–95 %B

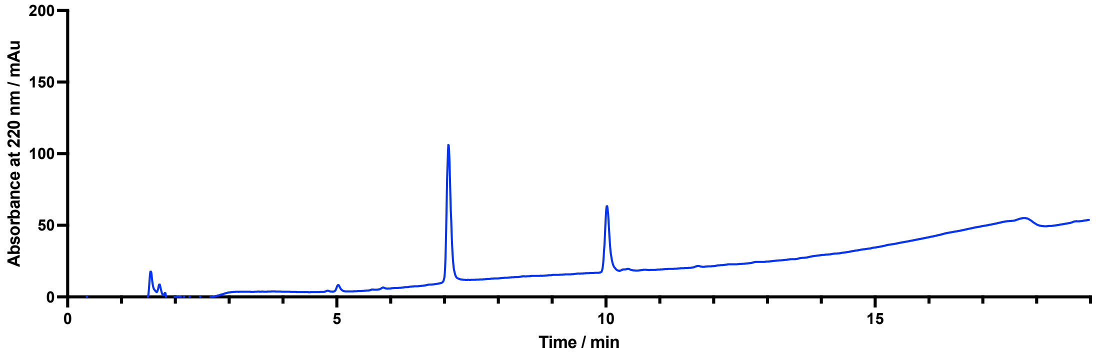

Stapled peptide P2-20, gradient 10–60 %B

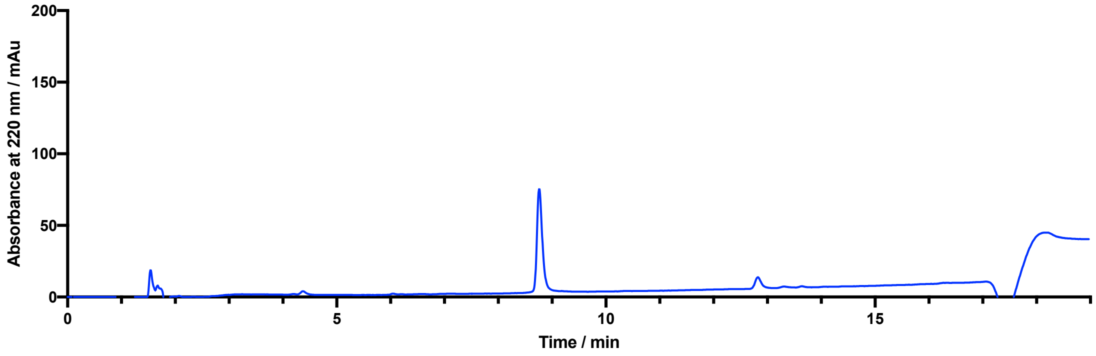

Stapled peptide P2-21, gradient 5–95 %B

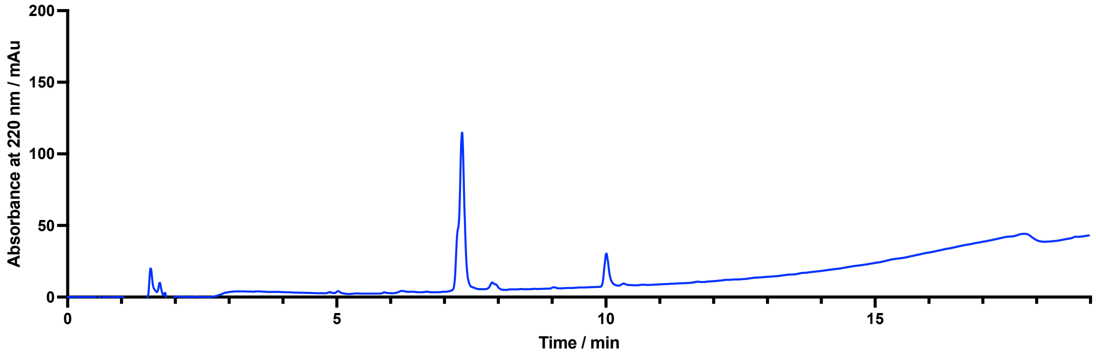

Stapled peptide P2-21, gradient 10–60 %B

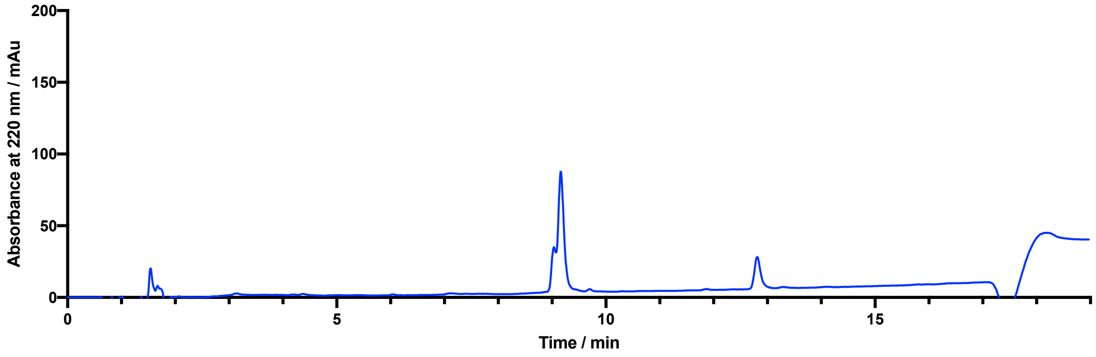

9 References

[1] A. V. Malkov, S. Stončius, K. N. MacDougall, A. Mariani, G. D. McGeoch and P. Kočovský, *Tetrahedron*, 2006, **62**, 264–284.

[2] M.-L. Pla, S. Oltra, M.-D. Esteban, S. Andreu and A. Palop, *BioMed Res. Int.*, 2015, **2015**, 1–14.

[3] L. D. Patterson and M. J. Miller, *J. Org. Chem.*, 2010, **75**, 1289–1292.

[4] R. Liu, P. A. Miller, S. B. Vakulenko, N. K. Stewart, W. C. Boggess and M. J. Miller, *J. Med. Chem.*, 2018, **61**, 3845–3854.

[5] S. J. Walsh, J. Iegre, H. Seki, J. D. Bargh, H. F. Sore, J. S. Parker, J. S. Carroll and D. R. Spring, *Org. Biomol. Chem.*, 2020, **18**, 4224–4230.

[6] M. J. Austin, S. J. Hearnshaw, L. A. Mitchenall, P. J. McDermott, L. A. Howell, A. Maxwell and M. Searcey, *Med. Chem. Commun.*, 2016, **7**, 1387–1391.

[7] L. Bauer, R. Manganaro, B. Zonsics, J. R. P. M. Strating, P. El Kazzi, M. Lorenzo Lopez, R. Ulferts, C. Van Hoey, M. J. Maté, T. Langer, B. Coutard, A. Brancale and F. J. M. Van Kuppeveld, *ACS Infect. Dis.*, 2019, **5**, 1609–1623.

[8] B. H. Gan, J. Gaynord, S. M. Rowe, T. Deingruber and D. R. Spring, *Chem. Soc. Rev.*, 2021, **50**, 7820–7880.

[9] V. Raussens, J.-M. Ruysschaert and E. Goormaghtigh, *Anal. Biochem.*, 2003, **319**, 114–121.

[10] V. Raussens, J.-M. Ruysschaert and E. Goormaghtigh, *Anal. Biochem.*, 2006, **359**, 150.

[11] V. Dinda, A. N. Kimang'a, D. Kariuki, A. W. Sifuna, T. J. O'Brien, M. Welch and O. N. Reva, *Access Microbiol.*, 2024, **6**, 000667.v4.

[12] J. Baranyi and T. A. Roberts, *Int. J. Food Microbiol.*, 1994, **23**, 277–294.

[13] R. Buchanan, R. Whiting and W. Damert, *Food Microbiol.*, 1997, **14**, 313–326.
